# A trailing ribosome speeds up RNA polymerase at the expense of transcript fidelity via force and allostery

**DOI:** 10.1016/j.cell.2023.02.008

**Published:** 2023-03-16

**Authors:** Liang Meng Wee, Alexander B. Tong, Alfredo Jose Florez Ariza, Cristhian Cañari-Chumpitaz, Patricia Grob, Eva Nogales, Carlos Bustamante

**Affiliations:** 1 QB3-Berkeley, University of California, Berkeley, CA, USA; 2 Biophysics Graduate Group, University of California, Berkeley, CA, USA; 3 Department of Chemistry, University of California, Berkeley, CA, USA; 4 Department of Physics, University of California, Berkeley, CA, USA; 5 Department of Molecular and Cell Biology, University of California, Berkeley, CA, USA; 6 Kavli Energy Nanoscience Institute, University of California, Berkeley, CA, USA; 7 Howard Hughes Medical Institute, University of California, Berkeley, CA, USA; 8 Molecular Biophysics and Integrated Bioimaging Division, Lawrence Berkeley National Laboratory, Berkeley, CA, USA; 9 These authors contributed equally; 10 Lead Contact

**Keywords:** Ribosome, RNA polymerase, translation, transcription, coupling, fidelity, allostery, force

## Abstract

In prokaryotes, translation can occur on mRNA that is being transcribed in a process called coupling. How the ribosome affects the RNA polymerase (RNAP) during coupling is not well understood. Here, we reconstituted the *E. coli* coupling system and demonstrated that the ribosome can prevent pausing and termination of RNAP and double the overall transcription rate at the expense of fidelity. Moreover, we monitored single RNAPs coupled to ribosomes and show that coupling increases the pause-free velocity of the polymerase and that a mechanical assisting force is sufficient to explain the majority of the effects of coupling. Also, by cryo-EM, we observed that RNAPs with a terminal mismatch adopt a backtracked conformation, while a coupled ribosome allosterically induces these polymerases toward a catalytically active anti-swiveled state. Finally, we demonstrate that prolonged RNAP pausing is detrimental to cell viability, which could be prevented by polymerase reactivation through a coupled ribosome.

## In brief

A combination of bulk, single molecule, high-throughput sequencing, and cryo-EM demonstrates that the ribosome, through the action of mechanical force and allostery, enhances the activity and reduces the fidelity of RNA polymerase during translation-transcription coupling.

## Graphical Abstract

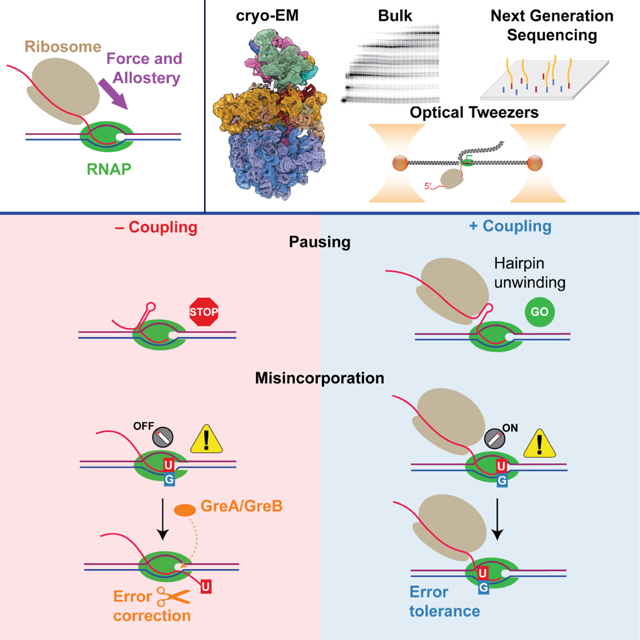

## INTRODUCTION

Transcription and translation are central gene expression processes subjected to extensive regulation. Transcription elongation by RNA polymerase (RNAP) in prokaryotes and eukaryotes are punctuated by pauses, which can guide proper folding of the nascent RNA,^1–14^ coordinate RNA synthesis with capping, splicing, methylation and polyadenylation,^15–18^ facilitate the correction of misincorporated ribonucleotides (rNTPs),^19–21^ serve as precursors for transcription arrest and termination^22,23^ and couple transcription with translation in bacteria.^24–26^ Interactions between RNAP and sequences of the DNA and RNA can trigger initial transient pauses that last for a few seconds;^27,28^ these *elemental* pauses can be precursors for longer-lived ones.^29–31^ For example, an RNA hairpin can stabilize an elemental pause through an allosteric interaction with the β-flap tip helix of RNAP.^32–34,34^ In this state, a *swivel module* in RNAP rotates and inhibits folding of the trigger loop (TL), thus keeping RNAP in an inactive “swiveled” conformation.^34,35^

A backtracking pause is another instance of a longer-lived pause, whereby RNAP moves backward and the 3′ end of the RNA moves beyond the catalytic site and extrudes into the secondary channel.^36–39^ Backtracking can occur when RNAP incorporates a wrong rNTP, when it encounters a physical barrier, or when it transcribes against a hindering load as applied in optical tweezers experiments.^38,40–43^ To recover from the backtracked state, RNAP must either translocate forward to return to its initial position on the DNA or cleave the extruding RNA, which can be assisted by transcription factors such as GreA and GreB. ^37,38,42,44–49^

In bacteria, the absence of a nuclear-cytoplasmic barrier means that ribosomes can translate RNAs as they emerge from RNAPs in a process known as *coupling*.^50–52^ A closely coupled ribosome prevents premature transcription termination by Rho or attenuation.^53–56^ Coupling also serves to coordinate translation and transcription.^57^ For example, it ensures protein expression in bacteria, where RNAs are uncapped and most have short half-lives;^58–60^ ribosomes translating on these RNAs can protect them from the decay machinery.^61^ A ribosome can also sequester nascent RNA and prevent its invasion and annealing to template DNA forming deleterious R-loops.^62^

While translation and transcription have been extensively studied in isolation, investigations into their mechanisms during coupling are still lacking, despite the recent progress made in elucidating transcription-translation coupling (TTC) structures.^63–70^ To address this shortcoming, we reconstituted an experimental system to study transcription during coupling *in vitro*.^71,72^ Employing bulk biochemical and single-molecule optical tweezers assays, next-generation sequencing, and single-particle cryo-EM, we show that translation reduces transcription pause entry and transcription termination by an RNA hairpin, and increases the inherent transcription activity (pausefree velocity). Significantly, we find that the ribosome increases the speed of transcription at the expense of decreased fidelity by exerting mechanical force on the RNAP and by allosterically inducing an active anti-swiveled conformation of the enzyme. We show that *in vivo*, a paused RNAP can cause ribosome-ribosome collisions which are costly for the cell to resolve and posit that the pause reduction caused by a coupled ribosome could prevent these collisions.

## RESULTS

### Development of a cell-free *in vitro* transcription-translation coupling system

**Figure 1.**
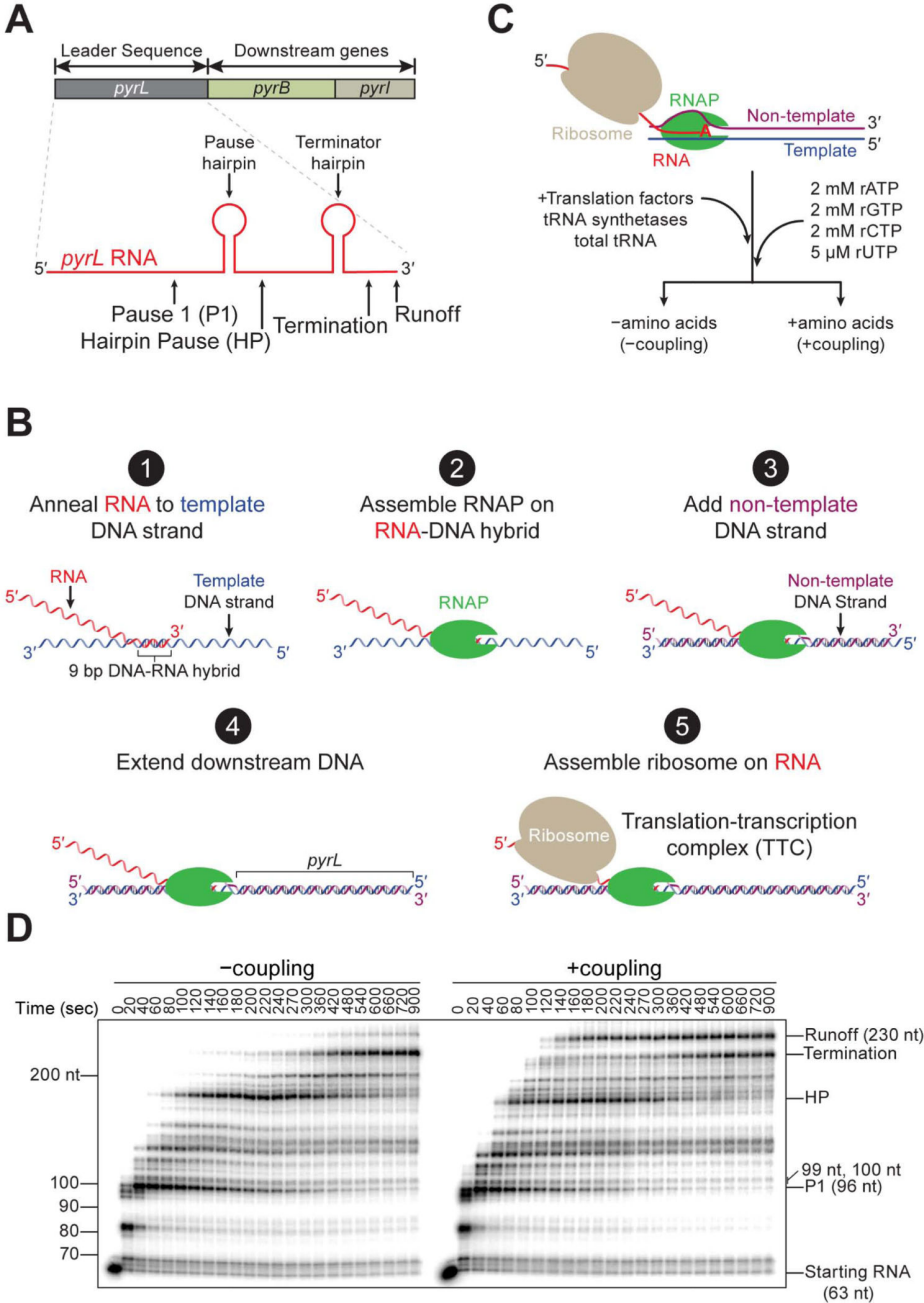
*In vitro* reconstitution of transcription-translation coupling (A) Schematic depicting the *pyr* operon. (B) Stepwise assembly of the translation-transcription complex (TTC). (C) Experimental scheme for −coupling and +coupling reactions. (D) Transcription under −coupling and +coupling reactions monitored with ^32^P-α-ATP-labeled transcripts in a denaturing urea gel. See also Figure S1.

The leader sequence (*pyrL*) regulates expression of the pyrimidine biosynthetic operon (*pyrBI*) in response to uridine triphosphate (rUTP) concentration. When rUTP is abundant, RNAP quickly transcribes *pyrL* and prematurely terminates when the emerging RNA folds into a terminator hairpin^73–75^ (Figure 1A). Under limiting rUTP concentration, however, RNAP frequently pauses on the mostly U-requiring template (49% of the sequence), which gives time for a coupled ribosome to prevent the folding of the terminator hairpin leading to the expression of the downstream *pyrB* gene that will replenish rUTP^76,77^ (Figure 1A). Therefore, we use the *pyrL* sequence to characterize transcription either in the absence (−coupling) or in the presence (+coupling) of active translation.

To assemble the TTC, we performed stepwise assembly of an elongating RNAP followed by the loading of a ribosome on the RNA that emerges from the polymerase (Figure 1B steps 1–5; see STAR Methods). We radiolabel the RNA to follow transcription over time in a denaturing urea gel^78–80^ (Figures 1C and 1D; see STAR Methods). Translation was enabled in the +coupling reaction by including all 20 amino acids, 20 tRNA synthetases, total tRNA, and the translation elongation factors Tu, G, and Ts^81–84^ (Tables S1A–S1F). In the −coupling reaction, we only omitted the 20 amino acids so that translation will not occur (Figure 1C; Tables S1A–S1F). Hence, any differences between the –coupling and +coupling conditions are due to the activity of the coupled ribosome. To mimic the condition of rUTP deprivation that facilitates coupling between the ribosome and RNAP on *pyrL*, rUTP was provided at 5 μM, whereas the rest of the rNTPs were present at 2 mM (Figure 1C; Tables S1A–S1F).

Using our reconstituted coupling assay we observed distinct gel bands that include those that correspond to RNAP at the previously described hairpin pause (HP) and the termination sites^76^ (Figure 1D). We noticed also the presence of an uncharacterized pause which we labeled as Pause 1 (P1) (Figures 1A and 1D). To determine the cause for pausing at P1, we performed exonuclease III digestion and found that RNAP is in a hyper-translocated register, wherein the polymerase has advanced by not one but two steps, leaving the 3’ end of the RNA inaccessible for ribonucleotide addition^85^ (Figure S1; see STAR Methods). We also observed an earlier appearance of longer RNAs in the +coupling condition, indicating an overall faster transcription rate than in the −coupling condition (Figure 1D). Additionally, the band at the termination site is less intense in the +coupling condition than in the −coupling condition, which points to fewer transcription termination events when RNAP is coupled to the ribosome (Figure 1D). This decrease in transcription termination is accompanied by significantly more runoff transcripts in the +coupling condition.

### Coupling reduces pause efficiency and the apparent pause duration at P1

**Figure 2.**
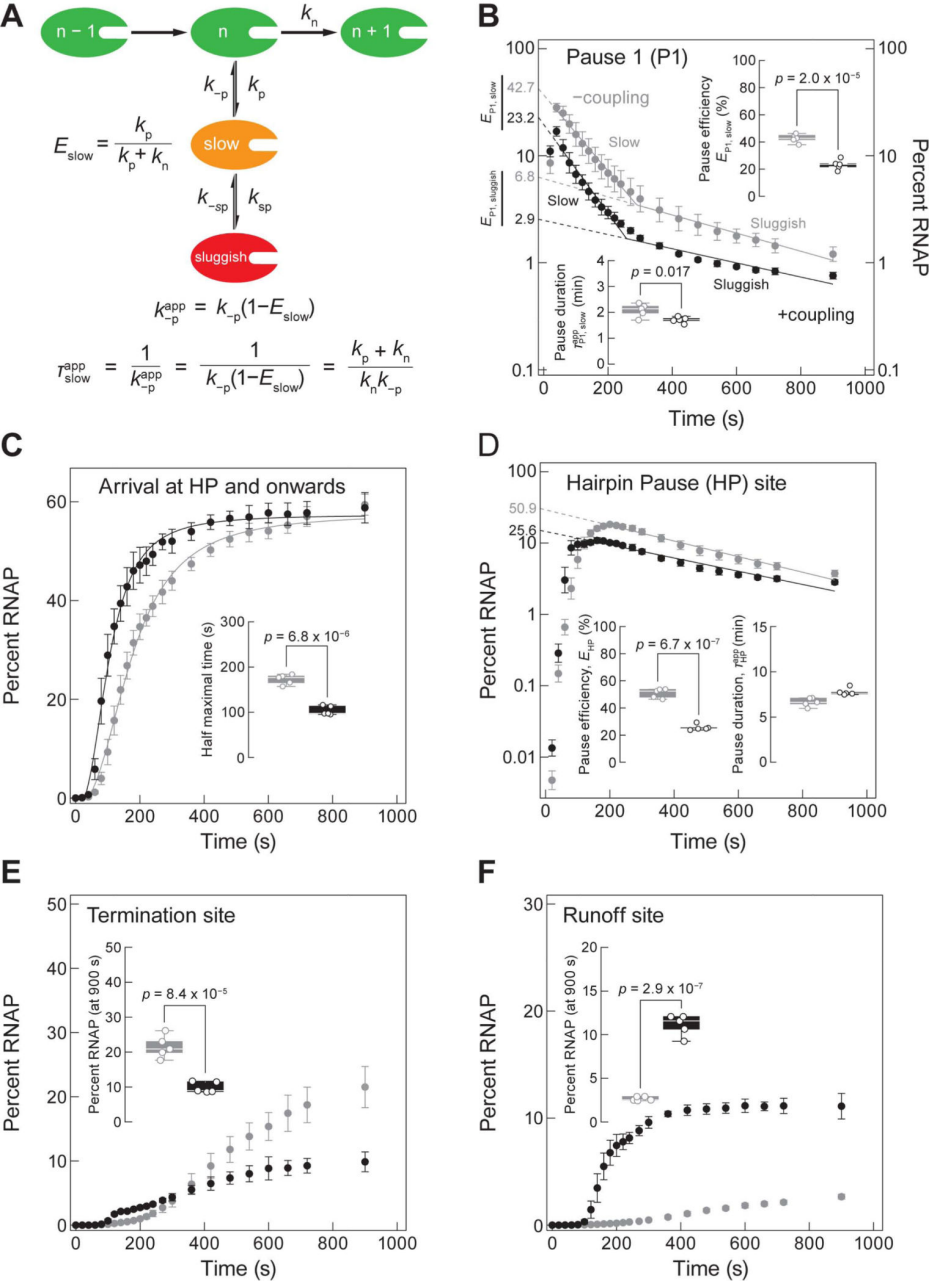
Translation speeds up transcription along *pyrL* during coupling (A) A simplified kinetic scheme for transcription. An active RNAP (green) transcribes with an on-pathway transcription elongation rate ($k_{\text{n}}$) that competes with an off-pathway pause entry rate ($k_{\text{p}}$). A paused RNAP (orange, ‘slow’ in P1) can return to the on-pathway state at an intrinsic rate given by *k*_−p_, or it can transition to an even slower state (red, ‘sluggish’ in P1). (B and D) Pause kinetics of RNAP at P1 (B) and at HP sites (D) in the −coupling (grey) and in the +coupling reactions (black). The decays in the percent of RNAP across time are fit to the sum of one or two exponentials: the values extrapolated to the y-axis correspond to their respective pause efficiencies, *E* (inset, right) while the slopes of the fits reflect the apparent pause duration, *T* (inset, left). (C) Arrival rate of RNAP at HP site and onwards in the −coupling and in the +coupling reactions. Inset: the half-maximal arrival time at the HP site. (E) Percent of RNAP that terminate in the −coupling and in the +coupling reactions. Inset: Percent RNAP present at the termination site for the final timepoint. (F) Percent of RNAP that bypass termination in the −coupling and in the +coupling reactions. Inset: Percent RNAP present at the runoff site for the final timepoint. For all graphs, data are mean ± SD for five independent experiments. See also Figure S2.

To interpret the kinetic information from our assay, we use a model where pauses manifest from the entry of RNAP to off-pathway states that compete kinetically with on-pathway elongation^29,86,87^ (Figure 2A). As such, the pause efficiency E is given by the branching ratio $E=k_{\text{p}}/\left(k_{\text{p}}+k_{\text{n}}\right)$, where $k_{\text{p}}$ is the pause entry rate, and $k_{\text{n}}$ represents the elongation rate (Figure 2A). To obtain the kinetic parameters associated with P1, we quantified the percent of RNAP at P1 for every timepoint. In a semi-log plot of the normalized percent RNAP present at P1 across time, we observed two populations of RNAP with different kinetic behaviors (Figure 2B). A two-segment linear fit reveals a slow and a sluggish RNAP, whose apparent pause durations ($T_{\text{P}1}^{\text{app}}=1/k_{-\text{p},\text{P}1}^{\text{app}}$) can be acquired from the slopes of the lines (Figure 2B). Extrapolating each linear fit to the y-axis allows us to calculate the percent of paused RNAPs that were slow ($E_{\text{P}1,\text{slow}}$), a portion of which then became sluggish ($E_{\text{P}1,\text{sluggish}}$). The remaining corresponds to the population of RNAP that bypassed pausing ($100\text{\%}-E_{\text{P}1,\text{slow}}$; Figure 2B).

In the −coupling condition, 42.7% of RNAP paused at P1. Among those that paused, 35.9 ± 3.6% (RNAP that was slow but not sluggish, ($E_{\text{P}1,\text{slow}}-E_{\text{P}1,\text{sluggish}}$)) added rUTP slowly with an average addition time ($T_{\text{P}1,\text{slow}}^{\text{app}}$) of 2.1 ± 0.3 min, while 6.8 ± 1.6% of RNAP was sluggish with a $T_{\text{P}1,\text{sluggish}}^{\text{app}}$ of 9.1 ± 0.8 min (Figures 2A and 2B; Table S2A). In the +coupling condition, we noticed a significant decrease in pausing efficiency from 42.7% to 23.2%, with proportional reductions in $E_{\text{P}1,\text{slow}}$ and $E_{\text{P}1,\text{sluggish}}$ (20.3 ± 3.7%, ~1.8-fold reduction and 2.9 ± 0.4%, ~2.3-fold reduction) (Figure 2B; Table S2A). We propose that the slow state is due to hyper-translocation. Given that the measured loading efficiency of the ribosome on RNA is 72 ± 3%, these effects are conservative lower bounds (Figure S2A). If every RNA-loaded ribosome could prevent pausing of RNAP at P1, we would expect 72% of the paused RNAP in the −coupling condition to bypass pausing. However, when comparing $E_{\text{P}1}$ between −coupling (42.7%) and +coupling (23.2%) conditions, our calculations revealed that only 46% ((42.7−23.2)/42.7; Figure 2B) fewer RNAP bypass pausing. Thus, not all loaded ribosomes rescued the RNAP at P1.

Upon exiting P1 with a rate of $k_{-\text{p},\text{P}1}$, RNAP can either proceed with on-pathway elongation or re-enter the pause (Figure 2A). The apparent pause escape rate constant, $k_{-\text{p},\text{P}1}^{\text{app}}$, is then the product of the intrinsic escape rate $k_{-\text{p},\text{P}1}$ and the probability that it will not re-enter the pause state: $k_{-\text{p},\text{P}1}^{\text{app}}=k_{-\text{p},\text{P}1}\left(1-E_{\text{P}1,\text{slow}}\right)$, which is equal to the inverse of the apparent pause duration $T_{\text{P}1,\text{slow}}^{\text{app}}$ (Figure 2A). If we assume that $k_{-\text{p},\text{P}1}$ is identical under −coupling and +coupling conditions, then the ~1.8-fold decrease in $E_{\text{P}1,\text{slow}}$ in the +coupling condition should induce a ~1.3-fold reduction in $T_{\text{P}1,\text{slow}}^{\text{app}}$. Indeed, our results showed that the presence of a ribosome resulted in a significant 1.2fold decrease in $T_{\text{P}1,\text{slow}}^{\text{app}}$ at P1 from 2.1 ± 0.3 min to 1.7 ± 0.1 min (*p* = 0.017; Figure 2B). Hence, the effect of the ribosome on RNAP at P1 can be rationalized simply by the reduction in $E_{\text{P}1,\text{slow}}$ without invoking a change in the pause exit rate $k_{-\text{p},\text{P}1}$ (Figure 2A).

The ribosome has two avenues by which it can affect the coupled RNAP: either by force—tugging on the RNA to push the RNAP forwards—or by allostery—eliciting an alternate enzymatic state of RNAP upon physical contact. The force exerted by the ribosome on RNAP is akin to an applied assisting force pushing the enzyme towards the direction of transcription, biasing a post-translocated state of the polymerase over a pre-translocated one and preventing its backtracking.^37,88^ Given that the ribosome can exert a significant forward-bearing force on RNAP^89^ and that the pause at P1 has a measurable component due to hyper-translocation, one could expect that upon encountering RNAP, the ribosome will likely exacerbate transcriptional pausing at P1 by shifting the equilibrium of RNAP further towards the hyper-translocated state over the post-translocated state. Accordingly, we would expect pausing to worsen under +coupling condition, but this is the opposite of what is observed (Figure 2B). Therefore, we propose that an allosteric effect, rather than a mechanical one, is responsible for reducing pausing at P1.

### Coupling increases overall transcription rate and eliminates hairpin–induced transcriptional pause and termination

We found that the half-maximal arrival time of RNAP at the HP site and onwards for the +coupling reaction of 104 ± 10 s, is ~1.7-fold shorter than for the −coupling reaction of 173 ± 11 s, demonstrating that the ribosome speeds up the overall transcription rate (*p* = 6.8 × 10^−6^; Figure 2C). At the HP site, in the −coupling reaction, the pause escape rate fits well to a single exponential with a pause efficiency ($E_{\text{HP}}$) of 50.9 ± 3.4%, which drops to 25.6 ± 2.2% in the +coupling reaction (Figure 2D), indicating that 50% ((50.9−25.6)/50.9) of paused RNAPs were rescued by the ribosome. We posit that the mechanism for pause suppression by the ribosome is either the unraveling of the hairpin directly^90^ or the sequestering of the hairpin sequence before it folds.^91,92^ By contrast, the apparent pause durations at the HP site ($T_{\text{HP}}^{\text{app}}$) are comparable: 6.7 ± 0.5 min and 7.8 ± 0.4 min in –coupling and +coupling reactions, respectively (Figure 2D), suggesting that the ribosome cannot rescue an RNAP that has already entered a hairpin pause, or this population arises from RNAPs without coupled ribosomes.

Just as a trailing ribosome reduces the pausing efficiency at the HP site, it also reduces the termination efficiency from 21.5 ± 3.2% (−coupling) to 9.9 ± 1.5% (+coupling) (Figure 2E). This fall in the percentage of transcription termination was accompanied by a complementary rise in the percentage of termination bypass (runoff) from 2.7 ± 0.2% (−coupling) to 11.1 ± 1.2% (+coupling) (Figure 2F). Similarly to the HP site, we propose that the ribosome unravels the terminator hairpin or prevents its formation. To support this model, we mimicked the unfolding of the putative terminator hairpin by force. We tethered RNAP and its nascent RNA in a custom-built optical tweezers instrument and applied a force of 15 pN on the RNA, which is sufficient to unfold most secondary structures, and scored for transcription termination as tether breakage caused by the release of RNAP from the DNA template (Figures S2B and S2C). Under 15 pN of force, 100% of RNAP (n = 50) bypassed termination (Figure S2B). When we instead dropped the force to 5 pN, which is too weak to unfold most RNA secondary structures, only 8% (N=37) bypassed (Figure S2C), similar to what has been previously reported.^93^ Overall, these findings show that a coupled ribosome decreases the efficiency of multiple types of RNAP pauses. As a further validation to show the effects observed in our transcription-translation coupling assay are ribosomespecific, we reassessed these effects in the presence of translation elongation inhibitors and see that the effect of coupling is effectively eliminated (Figures S2D–S2K; Tables S2A and S2B; see STAR Methods).

### Coupling increases the pause-free velocity and traveling distance of RNAP

**Figure 3.**
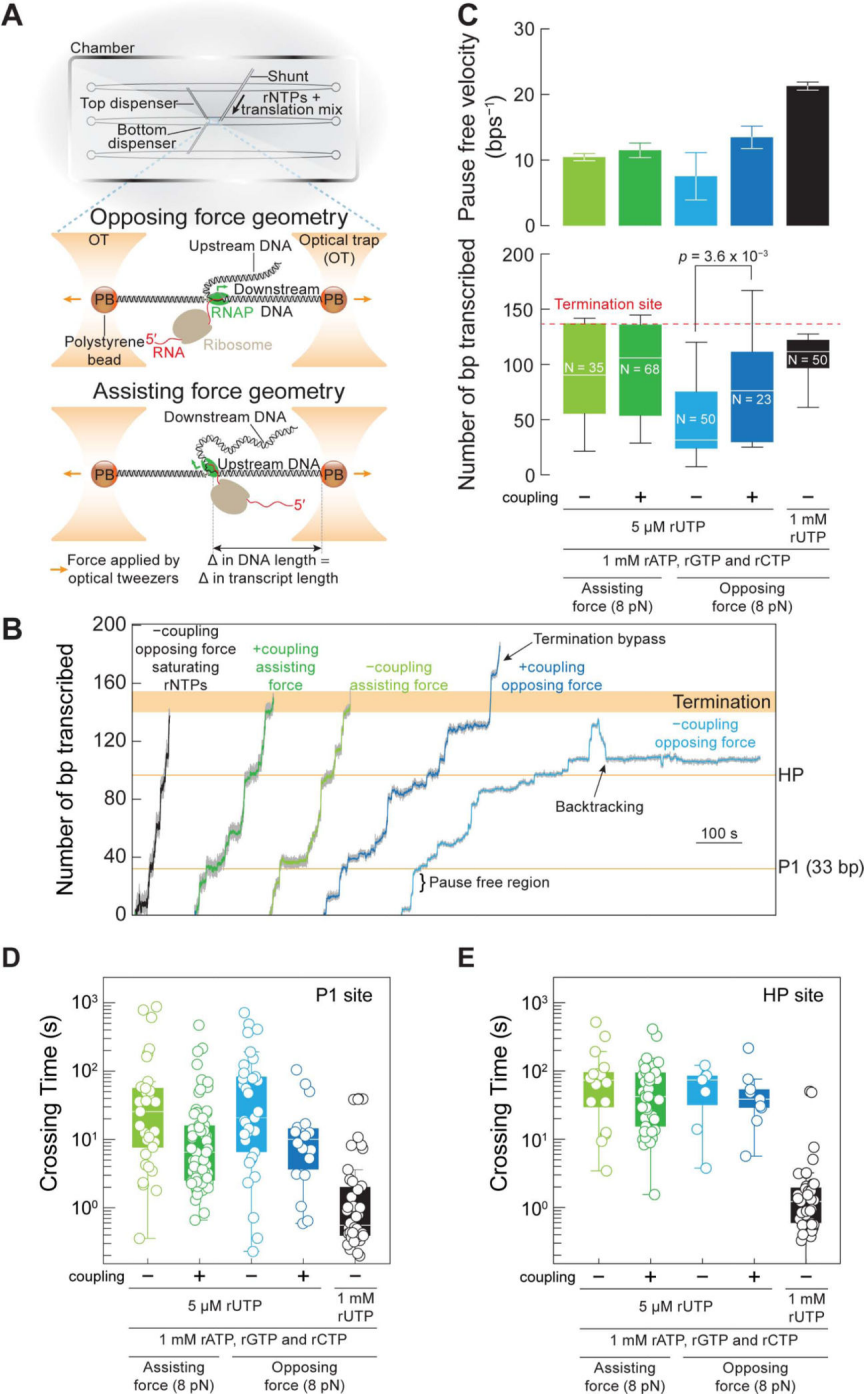
Coupling increases the pause free velocity and traveling distance of RNAP (A) Optical tweezers setup to monitor transcription. Top, the microfluidics chamber in which the optical tweezers experiments are performed. Middle, RNAP was tethered via the C-terminus end of its ${\text{β}}^{\prime }$ subunit and the downstream DNA template. In this geometry, applied force hinders transcription and hence is also known as an opposing force experiment. By contrast, tethering via the upstream DNA will assist transcription under applied force (bottom). The direction of transcription by RNAP is indicated by a green arrow. (B) Representative transcription traces by RNAP under opposing force in saturating rNTPs and under opposing or assisting force with and without the ribosome under limiting rUTP (5 μM). (C) The average pause-free velocities of RNAP (top panel) and distances traversed (bottom panel) for the five conditions in (B) are shown. Data are mean ± SEM (top panel) and the box-and-whisker plots denote quartiles (bottom panel). (D and E) The crossing times at pause P1 (D) and HP (E) for the five conditions in (B) are shown as a beeswarm. The box-and-whisker plots denote quartiles. See also Figure S3.

To see whether coupling affects the pause-free velocity (PFV, which is essentially $k_{\text{n}}$) of the enzyme, we turned to optical tweezers and monitored single RNAPs transcribing under similar –coupling or +coupling conditions used in the bulk studies (Figure 1B; Tables S1G–S1K). Using a high-resolution dual-trap optical tweezers instrument equipped with single molecule fluorescence detection capability, we can track the position of an RNAP on its DNA template in the optical tweezers channel and confirm the presence of a ribosome through the fluorescence signal of the initiator fMet-tRNA^fMet^-JF549 (Figure 3A; see STAR Methods). Additionally, this experimental setup allows us to determine if the ribosome is active by the subsequent loss of the fluorescence signal (Figure S3A). The RNAP was held between two optically-trapped beads linked by DNA handles, and its activity was monitored either in an assisting force geometry, where applied force *assisted* the movement of the enzyme or in an opposing force geometry where the applied force *opposed* its motion (Figure 3A). The act of transcription will lead to an increase (assisting force) or a decrease (opposing force) in the inter-bead distance with time (Figure 3A; see STAR Methods).

From individual single molecule transcription trajectories, we identified regions where RNAP transcribed without pausing to calculate the PFV (Figure 3B; see STAR Methods). Under opposing force, we observed an increase in the PFV in the presence of the ribosome that is on par with the speed attained by RNAP under assisting force (Figure 3C; Table S2C). In contrast, the speeds in the assisting force mode are not significantly affected by the ribosome (Figure 3C; Table S2C). These velocities are much slower than that when rUTP is saturating, indicating that the PFVs include the longer dwells (on-pathway incorporation times, that are distinct from off-pathway pausing) that must result from the low concentration of rUTP (Figure 3C; Table S2C). We consider these rUTP incorporation events distinct from pausing since pausing by definition is an off-pathway state and typically lasts for seconds, not the 0.11 s that would be expected for rUTP incorporation given a $V_{\max}$ of 40 nts^−1^ and $K_{\text{M}}$ of 16 μM ^94^. As mentioned above, a kinetic competition exists between the rate of pause entry ($k_{\text{p}}$) and the forward transcription rate ($k_{\text{n}}$), so an increased PFV should result in a decreased efficiency of pausing (Figure 2A): indeed, this trend is experimentally observed (Figures 3B and S3B). Similar trends are observed for the final transcript lengths. The RNAP alone under opposing force and limiting rUTP only makes it 41 bp into the template, a coupled ribosome doubles this distance to 76 bp (Figure 3C). Under assisting forces, the RNAPs transcribe further than under opposing forces and are not greatly affected by the ribosome (Figure 3C).

The times spent by RNAP at the major pauses at P1 and the HP site were calculated for the optical tweezers data as crossing times, the time it takes for the trace to cross a small window around the pause location (see STAR Methods). For P1, the distributions of these crossing times are largely insensitive to the direction of the applied force, having similar distributions in both assisting and opposing force conditions (Figure 3D). The ribosome, however, is able to greatly shorten the crossing time distributions at P1 in both cases (Figure 3D). A similar trend is seen for the pause durations at HP site (Figure 3E). If we fit the pause duration distribution at P1 to a sum of two exponentials, one representing the RNAPs that pause and one that represents those that didn’t, we can extract from the fit parameters the pause efficiency and the pause durations for these pauses (Figure S3C; see STAR Methods). The pause efficiencies and durations of pausing at P1 are similar to those found in the bulk experiment (Figures 2B and S3D; Table S2C). Interestingly, we see that the pause durations at P1 are slightly longer in the assisting force condition compared to the opposing force condition (Figure S3D), supporting our hypotheses that pausing at P1 is due to a hyper-translocated RNAP and the existence of an allosteric effect of the ribosome.

### Coupling increases ribonucleotide misincorporation

**Figure 4.**
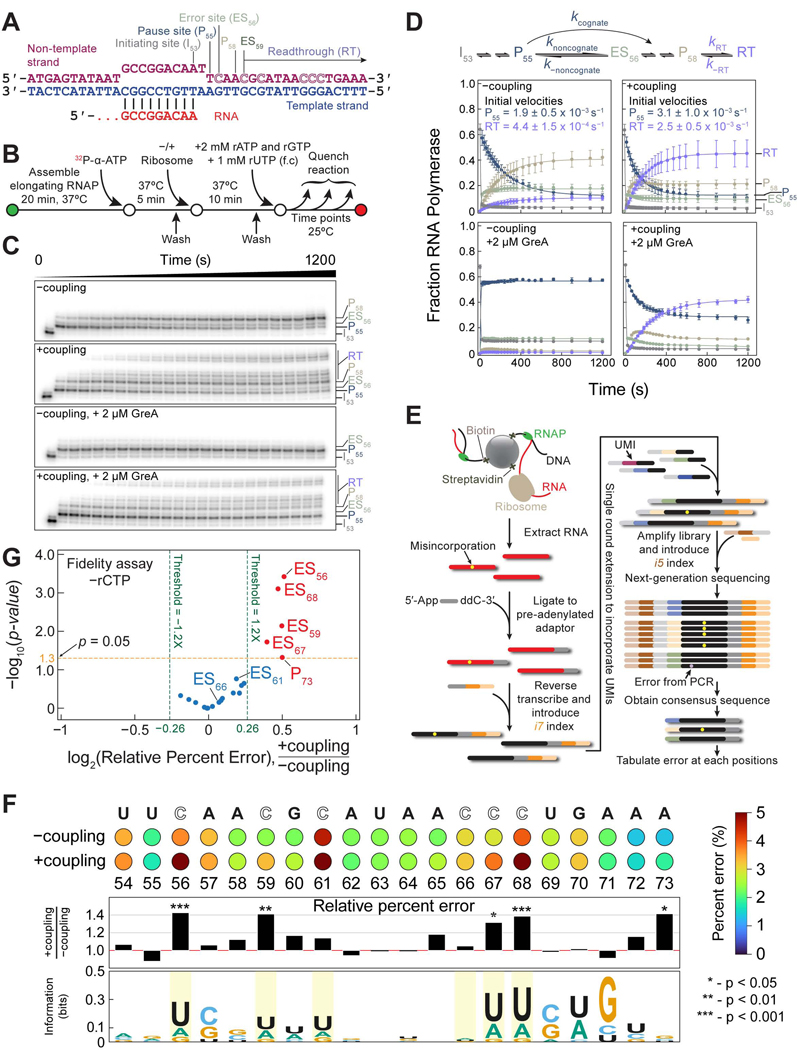
Coupling reduces transcription fidelity (A) Schematic showing the RNA (red) annealed to the template DNA (blue) and non-template DNA (purple) of the assembled RNAP. (B) Experimental scheme of the fidelity assay. (C) Denaturing urea gel showing the progress of transcription with time for the −coupling and the +coupling reactions +/− 2 μM GreA. (D) Quantification of the fidelity assay depicted in (B) and in (C). Data are mean ± SD for three independent experiments. (E) Workflow to prepare sequencing libraries to identify transcription errors. (F) Top panel: Percent transcription error in the −coupling and the +coupling reactions. Middle panel: Relative percent error of the +coupling to the −coupling reactions. Bottom panel: Sequence logos showing misincorporated ribonucleotides identified at each position. (G) Volcano plot highlights an increased tendency for RNAP to misincorporate during coupling. See also Figure S4.

Because mutations in the TL of RNAP that boost transcription rate also compromise transcription fidelity,^95–97^ we wondered whether coupling also increases transcription misincorporation rate. We adapted the fidelity assay of Erie and coworkers to identify and quantify misincorporation events by RNAP on a template encoding partial ${\mathrm{\lambda P}}_{\text{R}}$ promoter sequences when rCTP is absent while rATP, rGTP, and rUTP are present in excess^19^ (Figures 4A and 4B; Tables S1L–S1O). As expected, RNAP paused at positions 55 and 58, immediately upstream of template sites specifying for rCTP (${\text{P}}_{55}$ and at ${\text{P}}_{58}$; Figures 4A and 4C). RNAP escapes pausing at ${\text{P}}_{55}$ with a faster initial rate in the +coupling reaction (3.1 ± 1.0 × 10^−3^ s^−1^) than in the −coupling reaction (1.9 ± 0.5 × 10^−3^ s^−1^, *p* = 5.1 × 10^−3^; Figure 4D). We also observed a 6-fold increase in the initial rate of accumulation of readthrough (RT, bands beyond ${\text{P}}_{58}$) RNA for the +coupling reaction (2.5 ± 0.5 × 10^−3^ s^−1^) compared to the −coupling reaction (4.4 ± 1.5 × 10^−4^ s^−1^, *p* = 2.5 × 10^−7^; Figure 4D), and is accompanied by a 4.5-fold more RT product in the +coupling case than in the −coupling case. Our results reveal that coupling promotes transcription beyond ${\text{P}}_{55}$, and across multiple sites that require rCTP addition, into RT.

Position 56 specifies for rCTP and has a noticeable pause, which suggests that misincorporation has occurred at this position and interfered with the subsequent addition of rATP, which is in abundance (Figures 4B and 4C). Therefore, we designated positions that specify for rCTP as error sites (ES) since we speculate that they are more likely to contain errors in the absence of the cognate rCTP. Additionally, the abundance of non-cognate rNTPs in the reaction can drive a high rate of misincorporation and explain the rapid achievement of steady state by RNAP at ${\text{ES}}_{56}$ (Figures 4C and 4D). A steady state at ${\text{ES}}_{56}$ implies that the rate of exit from this error site, either by returning to ${\text{P}}_{55}$ to enable error rectification by endonucleolytic cleavage or by progressing forward, must be comparable to the misincorporation rate. The former scenario of the exit strategy requires RNAP to backtrack, which, in the +coupling case, will be hindered by a closely linked ribosome. In support of this interpretation, we added GreA *in trans* and find that, in the –coupling case, the majority of RNAP return to ${\text{P}}_{55}$, while in the +coupling case, GreA addition has only minor effects and importantly does not prevent RNAP from progressing past ${\text{ES}}_{56}$ (Figures 4C and 4D). Therefore, in the presence of the ribosome, the steady state observed at ${\text{ES}}_{56}$ implies that the rate of entry into the error site is mostly balanced by the rate of continued elongation with tolerance for the mistake. Accordingly, RNA should contain more mistakes at ${\text{ES}}_{56}$ and at other ES sites in the +coupling reaction than in the −coupling reaction.

To validate this prediction, we isolated close to full-length RNAs and prepared libraries for high-throughput sequencing (Figure 4E; see STAR Methods). We quantified the nucleotide identities from ${\text{P}}_{54}$ to ${\text{P}}_{73}$ of the RNA and found that, by tabulating the relative percent error between the +coupling and the −coupling reactions at each position, we were able to reveal substantial increases in misincorporation at four out of the six ES sites (67%; Figure 4F). Fittingly, these four ES sites, which include ${\text{ES}}_{56}$, are found clustered in the upper right region of a volcano plot, representing high relative percent error (+coupling/−coupling) that are statistically significant (*p* < 0.05; Figure 4G). Uridine is preferentially misincorporated at these sites (Figure 4F). Apart from ES sites, only ${\text{P}}_{73}$ shows significant relative percent error (Figures 4F and 4G). We also found that RNA containing one or more misincorporations increases from 16% for the −coupling reaction to 23% for the +coupling reaction (Figure S4A).

We similarly examined misincorporation in our earlier bulk transcription of *pyrL* under the condition of limiting rUTP (Figures 1C and 1D). Strikingly, we noticed a propensity for transcription misincorporation to occur at template sites specifying for rUTP (U sites) that are immediately downstream of sites specifying for rCTP regardless of coupling (Figure S4B). Indeed, it was previously reported that rCTP at the 3′ end of the RNA increased misincorporation rate by RNAP.^98^ Using the relative percent error metric, we identified 20 positions that had significant misincorporation during coupling and resided in the upper right region of the volcano plot (Figures S4B and S4C). For these 20 positions, an overwhelming majority of the misincorporated rNTPs are pyrimidines. Of these 20 positions, 15 (~19% of non-U sites) do not code for rUTP, highlighting the fact that mistakes can also occur at non-U sites where their cognate rNTPs are in abundance. Conversely, misincorporations do not preferentially occur at U sites despite the 400-fold lower concentration of the cognate rUTP (5 μM) compared to the rest of the rNTPs (2 mM of each). In fact, only 5 out of the 37 U sites (~14%) registered significant misincorporation events during coupling (Figures S4B and S4C). Finally, like the fidelity experiment, we observed a modest increase in RNA having one or more misincorporations from 37% for the −coupling reaction to 39% for the +coupling reaction (Figure S4A).

A higher tendency to misincorporate at ES sites (67%) than at U sites (14%) may be explained by the longer transcriptional pauses at ES sites than at U sites because rCTP was absent in the fidelity experiment as opposed to rUTP, which was present at 5 μM in the *pyrL* experiment. The extended pause duration at ES sites will provide more time for the ribosome to act on RNAP and increase the probability of misincorporation by the polymerase. Collectively, our sequencing data clearly demonstrate that RNAP is generally more prone to misincorporate when coupled to the ribosome.

### Mechanical force aids RNAP in overcoming mismatch-induced pausing

**Figure 5.**
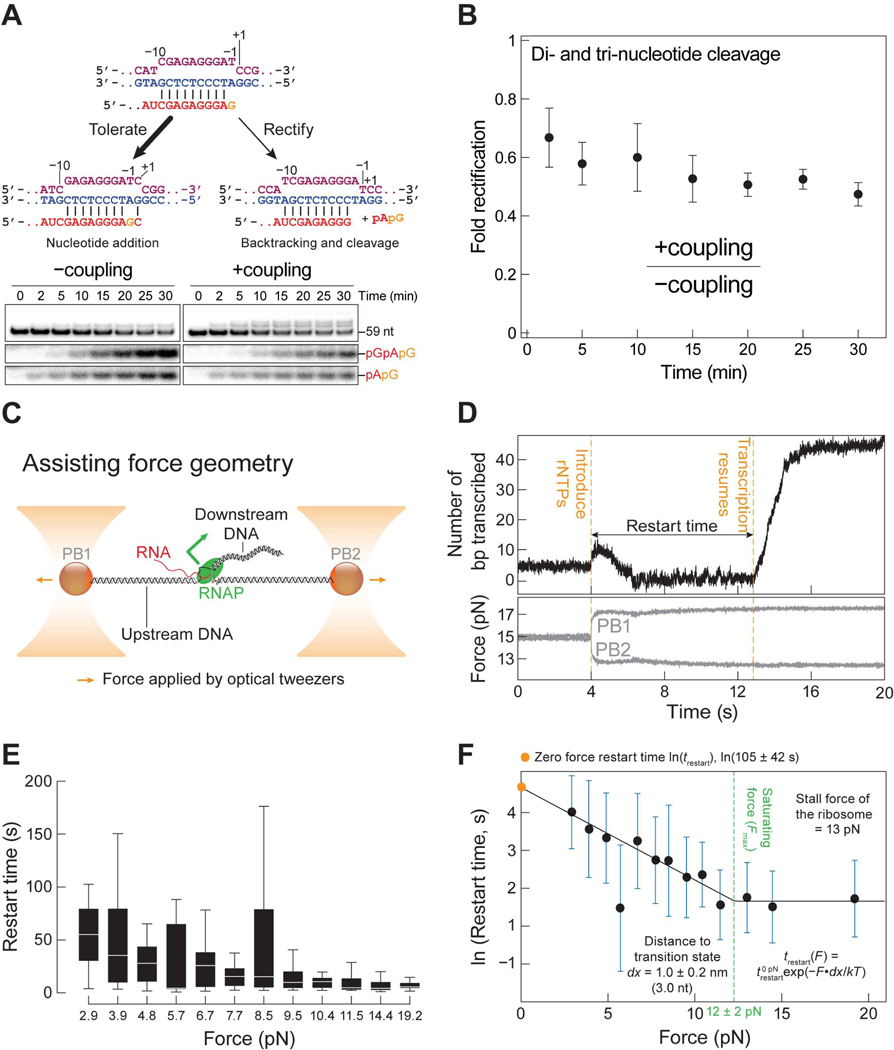
RNAP overcomes mismatch-induced pausing under assisting force (A) RNAP was assembled at the pictured site with a 59 nt RNA and was forced to misincorporate with 1 mM rGTP. The +coupling reaction produces less di- (or tri-) nucleotide products (reduced error rectification by RNAP) and more extended RNA (increased error tolerance) than in the −coupling reaction. (B) Quantification of the gel in (A), as the ratio of the di- and tri-nucleotides band intensities in +coupling/–coupling. Data are mean ± SD for three independent experiments (C) RNAP with a terminal rU-dG mismatch tethered in an assisting force geometry. The direction of transcription is indicated by the green arrow. (D) A representative trace for transcription restart of RNAP held at an average constant force of ~15 pN. When the shunt is opened to introduce rNTPs, the force channel (bottom panel) registers a slight change in force due to fluid flow. (E) Restart time decreases with increases in force. Each force range is from at least N=8 tethers. The restart time at each force range is depicted by the box-and-whiskers plot, which denotes quartiles. (F) The restart kinetics can be modeled by an Arrhenius equation, which suggests an exponential dependence of restart time (*t*) on force (*F*). The blue error bars denote the standard deviation for the restart time at each force. Fit uncertainties are 95% CIs. See also Figure S5.

When burdened with a terminal mismatch, RNAP can either tolerate or rectify the mistake (Figure 5A). To fix the error, RNAP backtracks by one base pair and removes the wrongly incorporated rNTP as a dinucleotide.^19,99–101^ Again, we reason that a tightly coupled ribosome can prevent backtracking of RNAP and obstruct error correction by RNAP. Using internal labeling with ^32^P-α-ATP, we can identify and quantify the cleaved dinucleotide as a measure of the extent of error correction by the polymerase (Figures 5A, S5A and S5B). We also took advantage of the unexpected observation that the reaction performed at 37°C as opposed to at 25°C drives more misincorporation by RNAP under saturating concentration of the non-canonical rGTP (Figure S5C). Under these conditions, we observe that RNAP can backtrack by one or two base pair steps and remove the offending rNTP as a di- or tri-nucleotide fragment (Figure 5B). The abundance of these cleaved fragments decreases in the presence of the ribosome, strongly suggesting a reduced ability of RNAP to correct the error and is one mechanism to explain the observed increase in misincorporation rate caused by coupling (Figures 5A and 5B).

Apart from being a passive backstop, the ribosome can, in principle, exert a forward directing force on RNAP to impede backtracking, inhibit mistake rectification, and impell continuous rNTP addition.^89^ To test this hypothesis, we characterized the resumption of transcription by RNAP harboring an RNA with a terminal rU-dG mismatch at ${\text{ES}}_{56}$ when an assisting force is applied on the RNAP with optical tweezers, mimicking that exerted by the ribosome on the polymerase (Figure 5C). We withheld magnesium during the assembly of the mismatch-bearing RNAP to protect the offending ribonucleotide against intrinsic cleavage by the polymerase, except when required during the ligation reaction to attach the polymerase to polystyrene beads (Figures S5D and S5E). To quantify the relationship between force and the ability to resume transcription, we conducted experiments from 3 to 15 pN (Figures 5D–5F). At each force, we measured the restart time, which spans from the introduction of rNTPs to the restart of transcription (Figure 5D; see STAR Methods). We observed that as force increases, the median restart time decreases from 55 s at 3 pN to 4 s at 15 pN (Figure 5E). The force dependence fits well to an Arrhenius equation, which suggests an exponential dependence of force on restart time. The fitting allows us to propagate the pause duration to a value of 105 ± 42 s (95% CI) at zero force (Figure 5F). In addition, the distance to the transition state derived from the Arrhenius plot reveals that the RNAP is backtracked by 3.0 ± 0.6 nt (95% CI) at zero force, consistent with our observation that the mismatch-bearing RNA is cleaved by 2–3 nt in the presence of GreA or GreB (Figures S5F–S5H). Note that the restart time plateaus above 12 ± 2 pN (95% CI), a force attainable by the ribosome, whose stall force has been shown to be ~13 pN^89^ (Figure 5F). Thus, by mechanically pushing on RNAP, the ribosome can elicit tolerance to misincorporated rNTPs and reduce the pause duration by as much as 96%, enabling much faster resumption of transcription.

### Cryo-EM structure of RNAP harboring a terminal mismatch

**Figure 6.**
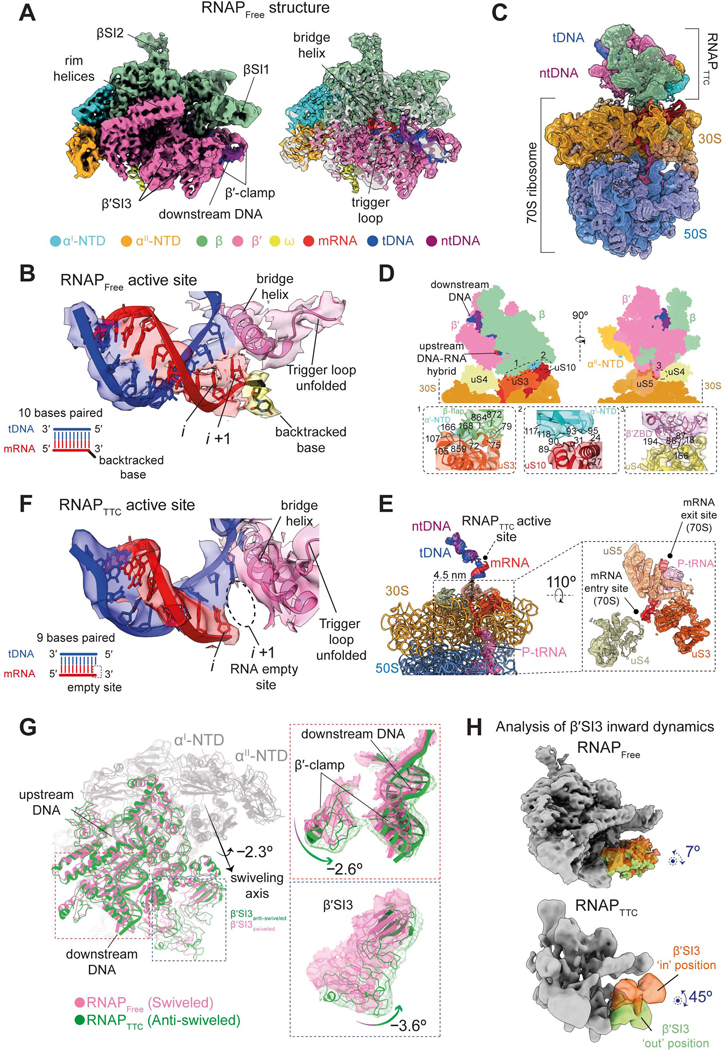
Cryo-EM structures of ${\text{RNAP}}_{\text{Free}}$, TTC and ${\text{RNAP}}_{\text{TTC}}$ (A) Left, cryo-EM structure of RNAP harboring a terminal mismatch (${\text{RNAP}}_{\text{Free}}$). Right, a cross-section of the EM reconstruction is shown fitted with the coordinate model. (B) Close-up view of the ${\text{RNAP}}_{\text{Free}}$ active site. ${\text{RNAP}}_{\text{Free}}$ is in a backtracked conformation, in which ten RNA bases appear hybridized with the tDNA up to the *i+1* position (threshold values at δ = 0.1–0.07) and the backtracked ribonucleotide is observed flipped out of the hybrid helix path (threshold values at δ = 0.05–0.04). (C) Combined cryo-EM structure of the TTC. (D) The RNAP-ribosome interaction surface. Top: cartoon views of RNAP and the ribosome 30S subunit in the TTC structure, in which potential intermolecular interactions are outlined by dashed boxes, with close-up views at the bottom. (E) Left, inner view of the TTC model showing the mRNA path from the ${\text{RNAP}}_{\text{TTC}}$ active site to the P-site tRNA.. (F) Close-up view of the ${\text{RNAP}}_{\text{TTC}}$ active site. ${\text{RNAP}}_{\text{TTC}}$ is in a post-translocated state, in which nine RNA bases appear hybridized with the DNA up to the *i* position. (G) Swiveling of ${\text{RNAP}}_{\text{Free}}$ versus ${\text{RNAP}}_{\text{TTC}}$. Coordinates of both structures were aligned relative to the core module of RNAP (gray ribbon) and it was observed that the swivel module of ${\text{RNAP}}_{\text{TTC}}$ was rotated −2.3° towards an anti-swiveled direction relative to that of ${\text{RNAP}}_{\text{Free}}$. Right, close-up views of the ${\text{β}}^{\prime }$-clamp, downstream DNA duplex and, ${\text{β}}^{\prime }$-SI3 regions. (H) Multibody analysis of the ${\text{β}}^{\prime }\text{SI3}$ domain ‘inward’ dynamics, in both the ${\text{RNAP}}_{\text{Free}}$ (top) and ${\text{RNAP}}_{\text{TTC}}$ (bottom). See also Figure S6, Data S1, Movies S1 and S2.

To establish whether the ribosome allosterically affects RNAP, we used cryo-EM to determine the structure of the RNAP harboring a terminal rU-dG mismatch at ${\text{ES}}_{56}$ in the absence (${\text{RNAP}}_{\text{Free}}$) or presence (${\text{RNAP}}_{\text{TTC}}$) of a coupled ribosome. We replaced the last three phosphodiester bonds of the RNA with phosphorothioate linkages to prevent hydrolysis of the mismatched ribonucleotides by RNAP.^102^ We obtained the 3D cryo-EM map of ${\text{RNAP}}_{\text{Free}}$ at 3.9 Å overall resolution (FSC = 0.143), and could confidently identify the subunits and nucleic acids (Figure 6A; Data S1).

The ${\text{RNAP}}_{\text{Free}}$, active site showed ten base pairs of the RNA-template DNA hybrid up to the *i+1* site. The mismatched rUTP was flipped out of the hybrid helix path (Figure 6B), indicating that ${\text{RNAP}}_{\text{Free}}$ is in a backtracked state, consistent with our GreA and GreB cleavage experiments and previous studies^42,100,103^ (Figures S5F–S5H). Moreover, the TL appears unfolded (Figure 6B), in agreement with related backtracked structures in which the extruded ribonucleotides hinder TL folding.^42^ Therefore, ${\text{RNAP}}_{\text{Free}}$ represents an off-pathway backtracked state unable to add rNTPs.

### RNAP adopts an ‘anti-swiveled’ post-translocated conformation in the presence of a ribosome

Initially, the consensus TTC reconstruction revealed an ill-defined density for RNAP, indicative of a heterogeneous orientation relative to the well-defined ribosomal density, suggesting that the ribosome might have translated to different positions on the RNA (see Data S1 and Movie S1). After discarding the set of TTC particles with the RNAP distant from the ribosome via multi-body refinement, we were left with a sizable population of particles (around 20%), from which we obtained a TTC reconstruction with highly reduced variability in the position of the RNAP. In this TTC, the 70S ribosome and RNAP regions were individually focus-refined to 3.8 Å and 7.3 Å overall resolutions (FSC = 0.143), respectively. Finally, the maps were combined, resulting in the full TTC (Figure 6C and Data S1).

The geometry of the RNAP in this map represents the most probable state, which occupies a ‘central’ position on top of the ribosome 30S subunit (Figure 6C). In this structure, the RNAP β-flap, ${\text{α}}_{\text{I}}$-NTD and ${\text{β}}^{\prime }$-zinc binding domain (${\text{β}}^{\prime }$-ZBD) are located close to the ribosomal proteins uS3, uS10, and uS4, respectively (Figure 6D). We observed clear density for RNA along the entry channel of the ribosome, lined by uS3, uS4, and uS5 ribosomal proteins (Figure 6E). In this present TTC structure, the position and interactions of the RNAP with the ribosome resembles the ‘expressomes’ or ‘collided TTCs’ previously described.^63,69,70^

In the ${\text{RNAP}}_{\text{TTC}}$ active site, 9 RNA bases are paired with the template DNA up to the *i* site, leaving the *i+1* site empty (Figure 6F). While it is not possible to assign the ribonucleotide identities in this structure, but given that we had incorporated three terminal phosphorothioate modifications in the RNA to prevent their hydrolysis, we conclude that ${\text{RNAP}}_{\text{TTC}}$ is in a post-translocated state in which the rU-dG mismatch is tolerated in comparison with the ${\text{RNAP}}_{\text{Free}}$, in which the polymerase is backtracked (Figures 6B and 6F).

It is known that the RNAP can adopt an inactive ‘swiveled’ conformation, as is the case when the RNAP pauses through the action of an RNA hairpin, or when it backtracks extensively. In this conformation, the swivel module (which includes the clamp, shelf, and ${\text{β}}^{\prime }\text{SI3}$ domain) can rotate relative to the core module in a plane approximately parallel to the one defined by the upstream and downstream DNA ends^34,42,104^. Also, in *E. coli*, in which the ${\text{β}}^{\prime }\text{SI3}$ domain is connected to the TL, the pronounced swiveling in inactive RNAP conformations (~ +4.5° to +6°) is incompatible with the ${\text{β}}^{\prime }\text{SI3}$ movements required for TL folding.^34,105^ On the other hand, in ‘active’ RNAP states, TL folding takes place alongside the inward rotation of the ${\text{β}}^{\prime }\text{SI3}$ domain towards the catalytic site.^42,105^

We compared ${\text{RNAP}}_{\text{Free}}$ and ${\text{RNAP}}_{\text{TTC}}$ by the alignment of their corresponding core modules and observed that the ${\text{RNAP}}_{\text{TTC}}$ swivel module is rotated by –2.3° relative to its position in the ${\text{RNAP}}_{\text{Free}}$, a conformation that we denote as the ‘anti-swiveled’ state (Figure 6G; Table S3). Moreover, when looking at specific regions within the swivel module, the ${\text{β}}^{\prime }$-clamp and ${\text{β}}^{\prime }\text{SI3}$ regions appear rotated –2.6° and –3.6°, respectively(Figure 6G; Table S3). Additionally, we compared the ${\text{RNAP}}_{\text{Free}}$ and ${\text{RNAP}}_{\text{TTC}}$ structures to a ‘non-swiveled’ RNAP containing DNA-RNA pairing (PDB:6ALH, ‘RNAP_6ALH_’).^106^ We see that the ${\text{RNAP}}_{\text{Free}}$ is swiveled by +1.5° compared to ${\text{RNAP}}_{\text{6ALH}}$, and that ${\text{RNAP}}_{\text{TTC}}$ is anti-swiveled, rotated by –1.7° compared to this reference (Figure S6A). We propose that the anti-swiveled ${\text{RNAP}}_{\text{TTC}}$ corresponds to an active polymerase since it can overcome and progress through mismatch-induced pausing (Figures 4C and 4D).

### RNAP harbors a large ${\text{β}}^{\prime }\text{SI3}$ mobility in the presence of the ribosome

Our active anti-swiveled ${\text{RNAP}}_{\text{TTC}}$ state should allow for TL folding, which is hindered in the swiveled, backtracked ${\text{RNAP}}_{\text{Free}}$. The folded state of the TL is very short-lived,^107^ so detecting it directly in our current experimental conditions is not possible. Rather, we looked for inward motions of the ${\text{β}}^{\prime }\text{SI3}$ domain since these motions are concurrent with TL folding.^42,105^ Thus, we performed multibody analysis in both the ${\text{RNAP}}_{\text{Free}}$ and ${\text{RNAP}}_{\text{TTC}}$ structures to analyze if ${\text{β}}^{\prime }\text{SI3}$ dynamics are different (Movie S2; see STAR Methods). We observed that in ${\text{RNAP}}_{\text{Free}}$, ${\text{β}}^{\prime }\text{SI3}$ motions are quite limited along different directions, with a maximum amplitude of ~7° for the inward rotation (Figures 6H top, S6B, and S6C; Movie S2). This observation agrees with the fact the backtracked ribonucleotide precludes TL folding in ${\text{RNAP}}_{\text{Free}}$.^42^ Strikingly, for ${\text{RNAP}}_{\text{TTC}}$, ${\text{β}}^{\prime }\text{SI3}$ exhibits an extensive range of motions along different directions, displaying up to ~45° of inward rotation to adopt the ‘in’ position that is conducive for TL folding (Figures 6H bottom, S6B, and S6D; Movie S2).

## DISCUSSION

### A trailing ribosome speeds up transcription by mechanical and allosteric means

Transcription by RNAP involves continuous ribonucleotide addition punctuated by pauses. Ribonucleotide addition involves rNTP binding, phosphodiester bond formation, and translocation by RNAP to restart the cycle. For simplicity, we have collapsed these three steps into one rate $k_{\text{n}}$ (Figure 2A). This active transcription rate $k_{\text{n}}$ competes kinetically with the pause entry rate $k_{\text{p}}$, and we see that a trailing ribosome skews this competition^29,86,108^ (Figure 2A). By exerting a forward-directing force on RNAP, the ribosome can bias RNAP into its post-translocated register and thus increase $k_{\text{n}}$. Additionally, this force prevents backtracking and can unfold hairpins, reducing $k_{\text{p}}$^109–111^(Figures 5E and 5F). In its capacity to allosterically induce RNAP to adopt an anti-swiveled conformation, the ribosome presumably discourages off-pathway transcriptional pausing (reduces $k_{\text{p}}$) and may even affect $k_{\text{n}}$ (Figure 6G). Taken together, our results indicate that, through mechanical and allosteric effects, the ribosome modulates $k_{\text{n}}$ and $k_{\text{p}}$ to improve overall transcription rate during coupling.

### Ribosome reduces the fidelity of RNAP

Ironically, the same mechanical and allosteric effects of the ribosome that increase transcription rate can also decrease transcription fidelity. Mechanical force promotes forward translocation of the mismatch-bearing RNAP to the post-translocated register, as seen in our structure, thus permitting continuous transcription and increasing the tolerance to error, as seen in our optical tweezers experiments(Figures 5E, 5F, 6B and 6F). In addition, the abutting ribosome obstructs RNAP backtracking and prevents editing of the offending ribonucleotide (Figures 4D and S5F–S5H). The allosteric activation of the RNAP by the ribosome counteracts the innate response to pause at a misincorporation and inhibits swiveling by the polymerase.^34,42,112^ Moreover, our results show that the ribosome keeps RNAP in an anti-swiveled state, allowing ${\text{β}}^{\prime }\text{SI3}$ domain to be placed closed to the catalytic site that is conducive for TL folding into TH for catalysis (Figures (Figures 6G, 6H bottom, S6A and S6B; Movie S2). Notably, the ${\text{β}}^{\prime }$ F1199 mutant that likewise biases ${\text{β}}^{\prime }\text{SI3}$ to assume a position close to the catalytic site (favors TH formation) also increases transcription misincorporation.^111^ While misincorporations are detrimental under normal conditions, it is theorized that during conditions of stress, they can cause phenotypic variability, which can lead to increased chances of survival for the bacterial population.^113–115^

### Transcription-translation coupling safeguards RNA and DNA integrity by preventing macromolecular collisions

A ribosome unable to rescue a stationary RNAP can also lead to a translational ‘traffic jam’ with ribosome-ribosome collisions in the nascent transcript. Recently, a ribosomal rescue pathway by the SmrB protein has been found to recognize the unique interface between collided ribosomes. SmrB cleaves this underlying mRNA in collided ribosomes leading to protein degradation and ribosome recycling by the SsrA system.^116^ We wondered if prolonged RNAP stalling can produce ribosome collisions and influence cell fitness. To do this, we performed a series of cell viability assays in which we stalled the RNAP via the addition of the antibiotic pseudouridimycin (PUM)^117^ to cells bearing various deletions of genes important for ribosomal rescue (*ssrA*, *smrB*, and the *smrB* paralog *smrA*). We observed that the ΔssrA strain showed severe growth defects compared to the WT strain, suggesting that *ssrA* is a key player for ribosomal rescue induced by a stalled RNAP (Figure S7A; see STAR Methods). By contrast, ΔsmrA and ΔsmrB strains grew better than the WT and ΔssrA strains, because less mRNA is cleaved and degraded in cells that have few mRNAs due to the PUM treatment (Figure S7A; see STAR Methods). These experiments suggest that the long pauses of an RNAP can be very costly to the cell because of the RNA degradation mechanism by which ribosomal collisions are resolved. We hypothesize that coupling can confer a fitness advantage by preventing ribosome-ribosome collisions by reducing the duration of pausing in RNAPs, even at the expense of fidelity (Figure S7B; see STAR Methods).

It has also been shown that a DNA polymerase that collides co-directionally with a stalled or backtracked RNAP triggers DNA damage.^118^ In the cell, error surveillance transcription factors such as GreA and GreB are tasked with assisting RNAP to promptly remove the offending ribonucleotide.^119,120^ Alternatively, Mfd and Rho ATPases can displace stalled RNAPs.^121–126^ We propose that by predisposing RNAP to overlook the mistake and continue to elongate, the ribosome acts as an additional line of defense to prevent RNAP roadblocks that hinder the movement of DNA polymerase that can lead to serious DNA damage. On the face of it, mistakes in RNA, which are transient and most likely inconsequential, as opposed to an alteration in the genomic DNA that is permanent and non-trivial, appear to be the lesser of two evils.

### Limitations of the study

In the *in vitro* experiments, we do not have complete loading of the ribosome (in actuality the loading rate is about 72% in the bulk experiments), nor can we ensure that the ribosome stays active for the entirety of the experiment. However, we still do see strong effects of a coupled ribosome, and as such these effects should be taken as a lower bound, and that there may be an observed weakening of the coupling effect on sites deeper into the RNA due to ribosomes becoming inactive mid-experiment.

Our work shows that the coupling of translation to transcription prevents prolonged stalling of RNAP at the expense of transcription fidelity through a combination of force and allostery *in vitro*. The next step is to demonstrate that this phenomenon also occurs *in vivo* by, e.g., conducting native elongating transcript sequencing experiments^127^ to determine if RNAP pausing increases upon the inhibition of ribosomes in bacteria, or by performing RNA-Seq to see if the rates of misincorporation decreases if translation is suppressed.

Our current cryo-EM reconstructions reveal that ${\text{RNAP}}_{\text{Free}}$ is in a backtracked state while ${\text{RNAP}}_{\text{TTC}}$ assumes a post-translocated register. The map, however, lacks the resolution to assign base identities of the RNA-DNA hybrid within the ${\text{RNAP}}_{\text{TTC}}$ to confirm that the terminal mismatch was indeed tolerated and retained as opposed to being removed.

## STAR METHODS

### RESOURCE AVAILBILITY

#### Lead Contact

Further information and requests for reagents and resources should be directed to and will be fulfilled by the Lead Contact, Carlos J. Bustamante (carlosb@berkeley.edu).

#### Materials availability

All unique/stable reagents generated in this study are available from the Lead Contact upon request and with a completed Materials Transfer Agreement.

#### Structural data availability

Cryo-EM density maps and fitted models have been deposited in the Electron Microscopy Data Bank (EMDB) and the Protein Data Bank (PDB). The cryo-EM maps for ${\text{RNAP}}_{\text{Free}}$, focused refinement ${\text{RNAP}}_{\text{TTC}}$ and the Ribosome_TTC_, have been deposited as EMD-29212, EMD-29213 and EMD-29214, respectively. The refined coordinate models have been deposited with PDB accession codes 8FIX, 8FIY, and 8FIZ, respectively.

#### Data and code availability

- The raw sequencing data generated in this paper are available for download on Mendeley data (dx.doi.org/10.17632/ysc6r3dz2m.1)
- All original codes have been deposited at Zenodo (dx.doi.org/10.5281/zenodo.6534021) and are publicly available as of the date of publication.
- Any additional information required to reanalyze the data reported in this paper is available from the lead contact upon request.

### EXPERIMENTAL MODEL AND SUBJECT DETAILS

#### Cell lines

Bacterial strains used in this work were obtained from sources described in Key Resources Table. MG1655 and MRE600 strain were maintained in LB media without antibiotics. Rosetta (DE3) pLysS cells was maintained on LB media but was cultured in 2YT media for protein expression at 16°C under ampicillin and chloramphenicol selection. BL21 strains were grown on LB media and inoculated into ZY media for protein expression by auto-induction at 37°C in the presence of ampicillin, kanamycin and chloramphenicol (for strain carrying pLysS). HB101 strain was maintained in LB media at 37°C and was transferred to terrific broth for tRNA expression under ampicillin selection. For ΔssrA, ΔsmrB, ΔsmrA and ΔsmrAΔsmrB strains, cells were grown at 37°C in LB media and then plated into Mueller-Hinton Agar plates for cell viability experiments.

### METHOD DETAILS

#### Oligonucleotides and RNA Preparation

DNA and RNA oligonucleotides (Table S4) were purchased from Integrated DNA Technologies (IDT). All oligonucleotides except primers for PCR were purified in house using denaturing urea polyacrylamide gels (PAGE) prepared from SequaGel UreaGel 29:1 Concentrate (National Diagnostics). RNA for bulk and single molecule optical trapping experiments were transcribed from synthetic DNA templates annealed to a generic oligonucleotide (CBD27) harboring the T7 promoter consensus sequence using the T7 MEGAscript^®^ kit (Life Technologies, Ambion). A typical transcription reaction was allowed to proceed for 4 hr at 37°C, DNase-treated and subjected to PAGE purification. Bands corresponding to the desired synthetic oligonucleotides and RNA were cut out as gel slices, eluted overnight at room temperature in 2X PK buffer (200 mM Tris-HCl, pH 7.5, 25 mM EDTA, pH 8.0, 300 mM NaCl and 2% SDS (w/v)), phenol chloroform extracted and precipitated with 3X volume of 200-proof 100% ethanol (Koptec). Then, samples were air dried and suspended in UltraPure^®^ DNase/RNase-free distilled water (Invitrogen, Thermo Fisher Scientific).

#### Ribosome preparation

We purified 70S ribosome from *E. coli* with slight modifications of previous protocols.^128,129^ First, log phase (${\text{A}}_{600}$ = 0.5) MRE600 cells were harvested (~2.4 g) and suspended in 15 ml lysis buffer (20 mM Tris-HCl, pH 7.5, 100 mM NH_4_Cl, 10 mM MgCl_2_, 0.5 mM EDTA and 6 mM β-mercaptoethanol) supplemented with cOmplete^™^, EDTA-free protease inhibitor cocktail (Roche) and 1500 units RNaseOUT^™^ (Invitrogen, Thermo Fisher Scientific). Cells were lyzed using S3000 Ultrasonic Liquid Processor with a microtip (Misonix) at power output setting of 8 in 50 ml glass beaker set in ice, 3 × 30 sec pulse with 30 sec cooling intermission. Lyzed cells were transferred to 50 ml Nalgene^™^ Oak Ridge Tubes (Thermo Fisher Scientific) and spun using JA-20 rotor in Avanti JXN-26 floor centrifuge (Beckman Coulter Life Sciences) at 16,000 rpm for 15 min at 4°C. Supernatant was collected and combined with 5 ml of lysis buffer rinse of the pellet (combined volume ~27 ml). The supernatant was layered over 35 ml of sucrose cushion (20 mM Tris-HCl, pH 7.5, 500 mM NH_4_Cl, 10 mM MgCl_2_, 0.5 mM EDTA, 6 mM β-mercaptoethanol and 37.7% (w/v) sucrose) in 70 ml polycarbonate tube with cap assembly (Beckman Coulter Life Sciences) and spun to pellet the ribosome using pre-cooled Type 45 Ti rotor (Beckman Coulter Life Sciences) at 33,000 rpm for 22 hr at 4°C. Next, the supernatant was discarded, and the ribosome-containing pellet was air dried for 10 min at 4°C. The semi-dried pellet was gently suspended in 2 ml of gradient buffer (10 mM Tris-OAc, pH 7.5, 60 mM NH_4_Cl, 7.5 mM Mg(OAc)_2_, 0.5 mM EDTA, 6 mM β-mercaptoethanol) for 2 hr at 4°C. Meanwhile, a gradient mixer was used to prepare six continuous 10–40% sucrose gradients starting from 18.5 ml of gradient buffer with 10% sucrose overlaid on top of the same buffer with 40% sucrose in opentop thick wall polycarbonate tubes (Beckman Coulter Life Sciences). The suspended ribosome (~85 mg/ml, estimated from absorbance at ${\text{A}}_{260}$) was split and layered across the six sucrose gradients (10–40% (w/v) sucrose), which were then subjected to high-speed spin using SW 32 Ti rotor (Beckman Coulter Life Sciences) at 22,000 rpm for 17 hr at 4°C. Following which, fractions of the samples were collected starting from the bottom of the sucrose gradient at a flow rate of 1.5 ml/min and monitored using the absorbance at ${\text{A}}_{260}$. Fractions that contain 70S ribosomes were harvested and pooled for the final round of centrifugation using Type 45 Ti rotor at a speed of 45,000 rpm for 20 hr at 4°C. The supernatant was discarded and the 70S ribosome pellet air dried for 10 min at 4°C. Finally, the ribosome pellet was gently suspended in gradient buffer at 4°C, aliquoted into 25 μl fractions, flash-frozen and stored at −80°C.

#### Protein expression by auto-induction in *E. coli*

Auto-induction allows for protein expression in bacteria (BL21 λDE3 or BL21 λDE3 pLysS strains) without the need to constantly monitor their growth and obviates the use of an inducing chemical such as Isopropyl β-D-1-thiogalactopyranoside.^130^ To begin, freshly-streaked bacteria were inoculated into rich media as starter cultures and grown with continuous shaking for 6–8 hr at 37°C. A 5 ml starter culture consisted of 4.65 ml of autoclaved ZY media (1% tryptone (w/v) and 0.5% yeast extract (w/v)), 2.5 μl 2 M MgSO_4_ solution, 100 μl of filter sterilized 40% glucose (w/v) and 250 μl of filter-sterilized 20X NPS (0.5 M (NH_4_)_2_SO_4_, 1 M KH_2_PO_4_ and Na_2_HPO_4_) supplemented with the appropriate antibiotic. After the initial growth, the starter culture was diluted 2000-fold into auto-inducing media, whereby cells were allowed to grow and express proteins for a total duration of ~16 hr at 37°C. The auto-inducing medium contained the same components as the starter culture except that filter-sterilized 50X 5052 solution (25% glycerol (w/v), 2.5% glucose (w/v) and 10% α-lactose (w/v)) was added (1X, f.c.) in place of the 40% glucose stock. For strains equipped with plasmids that need to be maintained by kanamycin, the concentration of the antibiotic was bumped up to 100 μg/μl to ensure adequate selection in rich media. To harvest the bacteria, the culture was cooled on ice, transferred to 1 L polypropylene bottle (Beckman Coulter Life Sciences) and then spun using JLA-8.1 rotor in Avanti JXN-26 floor centrifuge (Beckman Coulter Life Sciences) at 5000 rpm for 8 min at 4°C to pellet the cells. The cell pellet was then suspended in 1X phosphate-buffered saline (10 mM Na_2_HPO_4_, 1.8 mM KH_2_PO_4_, 137 mM NaCl and 2.7 mM KCl), re-pelleted and re-suspended for immediate cell lysis. Alternatively, the re-pelleted cells were weighed, flash-frozen and stored at −80°C.

#### Translation factors purification

Ribosome initiation factor 1 (IF1), 2 (IF2), 3 (IF3), elongation factor G (EF-G), elongation factor Tu (EF-Tu), elongation factor Ts (EF-Ts) were purified based on previous published protocols.^84^ All purification steps were conducted at 4°C. For all proteins, the peak fractions in the final step of the purification were harvested, pooled, concentrated in the final buffer and supplemented with glycerol (20% f.c., v/v). Finally, purified proteins were aliquoted, flash frozen and stored at −80°C.

##### Initiation Factor 1 (IF-1)

*E. coli* IF1 with a C-terminal (His)_6_-tag was expressed from pET24b-IF1 in BL21 strain by auto-induction, harvested, suspended in lysis buffer (10 mM Tris-HCl, pH 7.5, 60 mM NH_4_Cl, 10 mM MgCl_2_, 10 mM imidazole, 6 mM β-mercaptoethanol, ~3.75 U/μl benzonase (Novagen) and one tablet of cOmplete^™^, EDTA-free protease inhibitor cocktail (Roche)) and lysed by EmulsiFlex-C5 French Press (Avestin) at an internal cell pressure of 80–100 psi. The lysate was clarified twice by centrifugation at 20,000 rpm with JA-20 rotor for 30 min at 4°C. In the first step of the purification using immobilized metal ion affinity chromatography (IMAC), the supernatant was loaded on a 5 ml HisTrap^™^ HP column (GE Healthcare) equilibrated with IMAC buffer A (10 mM Tris-HCl, pH 7.5, 10 mM MgCl_2_, 30 mM imidazole and 5 mM β-mercaptoethanol). The loaded column was washed with 30 column volumes of IMAC buffer A (150 ml) before eluting IF1 using a linear gradient (0–100%) of IMAC buffer B (10 mM Tris-HCl, pH 7.5, 10 mM MgCl_2_, 500 mM imidazole and 5 mM β-mercaptoethanol). Fractions containing IF1 were pooled and loaded onto a 5 ml HiTrap^®^ SP HP column (GE Healthcare) pre-equilibrated with cation exchange binding buffer (25 mM Tris-acetate, pH 7.5, 50 mM KCl and 1 mM dithiothreitol). The loaded column was washed with 10 column volumes (50 ml) of binding buffer following which IF1 was eluted using a linear gradient (0–100%) of cation exchange elution buffer (25 mM Tris-acetate, pH 7.5, 1 M KCl and 1 mM dithiothreitol). IF1-containing fractions were pooled and concentrated with a 3K molecular weight cutoff (MWCO) Amicon^®^ Ultra centrifugal filter unit (Millipore) prior to its injection into HiPrep^™^ Sephacryl S100 16/60 column (GE Healthcare) for its final purification. The size exclusion column was equilibrated and ran at a flow rate of 0.5 ml/min with 20 mM Tris-acetate, pH 7.5, 95 mM KCl and 1 mM dithiothreitol.

##### Initiation Factor 2 (IF2) and 3 (IF3)

*E. coli* IF2 and IF3 with C-terminal (His)_6_-tags were expressed from pET24b-IF2 and pET24b-IF3 respectively. Similar purification procedures for IF1 were employed for IF2 and IF3 except that the buffer recipes were changed, cells were lysed by sonication (power setting of 8, 20 × 10 sec pulse with 1 min cooling intermission) and proteins were purified with cationic exchange 5 ml HiTrap^®^ Q HP columns (GE Healthcare). Lysis buffer and IMAC buffer A (50 mM HEPES, pH 7.5, 1 M NH_4_Cl, 10 mM MgCl_2_, 40 mM imidazole and 2 mM β-mercaptoethanol); IMAC buffer B (50 mM HEPES, pH 7.5, 100 mM KCl, 10 mM MgCl_2_, 1 M imidazole and 2 mM β-mercaptoethanol); anion-exchange binding buffer (50 mM HEPES, pH 7.5, 30 mM KCl, 5 mM MgCl_2_ and 1 mM dithiothreitol); anion-exchange elution buffer (50 mM HEPES, pH 7.5, 1 M KCl, 5 mM MgCl_2_ and 1 mM dithiothreitol). IF2 was purified using HiPrep^™^ Sephacryl S300 16/60 column (GE Healthcare) with 50 mM HEPES, pH 7.5, 100 mM KCl, 10 mM MgCl_2_ and 1 mM β-dithiothreitol.

##### Elongation Factor Tu (EF-Tu)

*E. coli* BL21 strain transfected with pCK-EF-Tu to express EF-Tu with an N-terminal (His)_6_-tag was lysed by sonication and subjected to IMAC, anion exchange and size exclusion chromatography. Lysis buffer (20 mM Tris-HCl, pH 7.5, 300 mM NaCl, 10 mM imidazole, 0.2 mM guanosine diphosphate (GDP), 0.5 mM MgCl_2_ and 2 mM β-mercaptoethanol); IMAC buffer A (20 mM Tris-HCl, pH 7.5, 300 mM NaCl, 30 mM imidazole, 0.2 mM GDP, 0.5 mM MgCl_2_ and 2 mM β-mercaptoethanol); IMAC buffer B (20 mM Tris-HCl, pH 7.5, 500 mM NaCl, 1 M imidazole, 0.2 mM GDP, 0.5 mM MgCl_2_ and 2 mM β-mercaptoethanol); anion exchange binding buffer (50 mM HEPES, pH 7.5, 30 mM KCl, 5 mM MgCl_2_ and 1 mM dithiothreitol); anion exchange elution buffer (50 mM HEPES, pH 7.5, 1 M KCl, 0.5 mM MgCl_2_, 0.2 mM GDP and 1 mM dithiothreitol); size exclusion buffer (50 mM HEPES, pH 7.5, 100 mM KCl, 0.5 mM MgCl_2_, 0.2 mM GDP and 1 mM dithiothreitol). After IMAC purification, the pooled and concentrated EF-Tu was diluted in 50 mM HEPES, pH 7.5, 0.5 mM MgCl_2_, 0.2 mM GDP and 1 mM dithiothreitol to lower the salt concentration prior to anion exchange purification. For the size exclusion chromatography, EF-Tu was purified using HiPrep^™^ Sephacryl S100 16/60 column (GE Healthcare).

##### Elongation Factor G (EF-G) and Elongation Factor Ts (EF-Ts)

*E. coli* BL21 strains expressing EF-G and EF-Ts with N-terminal (His)_6_-tags from pCK-EF-G and pCK-EF-Ts respectively were lysed by French Press and subjected to IMAC and anion exchange purification. Lysis buffer and IMAC buffer A (10 mM Na_2_HPO_4_, 1.8 mM KH2PO4, 137 mM NaCl, 2.7 mM KCl, 25 mM imidazole, and 2 mM β-mercaptoethanol, pH 7.5); IMAC buffer B (10 mM Na_2_HPO_4_, 1.8 mM KH_2_PO_4_, 137 mM NaCl, 2.7 mM KCl, 500 mM imidazole, and 2 mM β-mercaptoethanol, pH 7.5); anion exchange binding buffer (25 mM Tris-acetate, pH 7.5, 50 mM NaCl and 1 mM dithiothreitol); anion exchange elution buffer (25 mM Tris-acetate, pH 7.5, 1 M NaCl and 1 mM dithiothreitol); dialysis buffer (20 mM Tris-acetate, pH 7.5, 95 mM KCl and 1 mM dithiothreitol). After IMAC purification, pooled proteins were diluted 3-fold in 25 mM Tris-acetate, pH 7.5 and 2 mM β-mercaptoethanol to reduce salt concentrations prior to their loading onto 5 ml HiTrap^®^ Q HP columns (GE Healthcare). Following anion exchange purification, peak fractions of EF-G and EF-Ts were pooled and dialyzed twice, each time in 2 L of dialysis buffer using 6–8 kDa MWCO SpectraPor^®^ 1 dialysis membrane (Repligen).

#### tRNA synthetases purification

Expression plasmids for 20 tRNA synthetases carrying either N- or C-terminal histidine tags were cloned as described^71^ (Key Resources Table). They were kind gifts from Dr. Susan Marqusee. All synthetases were auto-induced in BL21 strains and extracted by sonication. Lysates were likewise clarified twice under high-speed centrifugation before column purifications. The enzymes were then captured on HisTrap^™^ HP columns (GE Heathcare) in binding buffer (50 mM HEPES-KOH, pH 7.5, 1 M NH_4_Cl, 10 mM MgCl_2_, 25 mM imidazole and 7 mM β-mercaptoethanol) and isolated from the column using a linear gradient (0–100%) of elution buffer (50 mM HEPES-KOH, pH 7.5, 100 mM KCl, 10 mM MgCl_2_, 1 M imidazole and 7 mM β-mercaptoethanol). Fractions that contained the enzymes were pooled, dialyzed into storage buffer (50 mM HEPES, pH 7.5, 100 mM KCl, 10 mM MgCl_2_ and 7 mM β-mercaptoethanol), concentrated with Amicon Ultra-15 Centrifugal Filter Units (Millipore Sigma) and supplemented with glycerol to 30% (v/v) before storage at −80°C. Aspartyl-tRNA synthetase and glutaminyl-tRNA synthetase were further cleaned up by HiTrap^®^ Q HP columns (GE Healthcare) prior to dialysis in storage buffer. The buffers for ion-exchange chromatography consisted of 50 mM HEPES-KOH, pH 7.5, 10 mM MgCl_2_ and 7 mM β-mercaptoethanol with either 50 mM (binding buffer) or 1 M (elution buffer) KCl.

#### Nucleotide diphosphate kinase purification

The coding sequence for nucleotide diphosphate kinase (NDK) was amplified from bacteria strain MG1655 (The Coli Genetic Stock Center, Yale) and cloned into pET His6 TEV LIC cloning vector, 2B-T (Addgene) to introduce an N-terminal (His)_6_ tag (pET2B-T-ndk). NDK was induced, extracted from cells and purified using the same procedures for tRNA synthetases. Similar buffer recipes were employed except that the starting final imidazole concentration in the binding buffer for IMAC purification was increased to 40 mM. NDK was concentrated (10,000 MWCO Amicon Ultra 15 concentrator) and further purified by size exclusion using HiPrep^™^ 16/60 Sephacryl S-100 column size exclusion chromatography (GE Healthcare) with buffer that consists of 50 mM HEPES-KOH, pH 7.5, 100 mM KCl, 10 mM MgCl_2_ and 1 mM dithiothreitol. NDK containing fractions were pooled, concentrated, and adjusted to 30% glycerol (v/v) for storage at −80°C.

#### Methionyl-tRNA formyltransferase purification

The coding sequence for methionyl-tRNA formyltransferase (FMT) was amplified from bacteria strain MG1655 (The Coli Genetic Stock Center, Yale) and cloned into pET2Bc-T vector (Addgene) to give pET2Bc-T-fmt. Similar purification procedures and buffers to those of NDK were employed to purify FMT except that all buffers contain 1 mM (f.c) DTT as reducing agent.

#### Evolved sortase A purification

An evolved P94R/D160N/D165A/K190E/K196T mutant version of *Staphylococcus aureus* sortase A (esrtA) that exhibits 140-fold increase in $k_{\text{cat}}$/$K_{\text{M}}$ compared with the wild type enzyme was used to introduce biotin at the C-terminus of the ${\text{β}}^{\prime }$ subunit of *E. coli* RNA polymerase (RNAP).^131^ The plasmid expressing esrtA (pET29-eSrtA) was obtained from Dr. David Liu. We auto-induced the C-terminal (His)_6_-tagged esrtA in BL21 strain, which was lysed by sonication and spun twice with JA-20 rotor at 20,000 rpm for 30 min to clarify the lysate. The enzyme was purified using a HisTrap^™^ HP column (GE Healthcare) and eluted in a linear gradient of 40 mM–1 M imidazole in 50 mM Tris-HCl, pH 7.5, 300 mM KCl, 0.1 mM EDTA and 1 mM β-mercaptoethanol. The protein was further cleaned up using HiPrep^™^ 16/60 Sephacryl S-100 size exclusion chromatography (GE Healthcare) with 25 mM Tris-HCl, pH 7.5, 150 mM KCl, 5 mM CaCl_2_ and 0.1 mM dithiothreitol. Finally, esrtA was concentrated and stored in the presence of 30% glycerol (v/v).

#### RelE Purification

We purified RelE based on previous described protocols but with slight modifications.^132,133^ Here, we auto-induced expression of RelE with pET22b-Δ9-His6x-RelB:RelE in BL21 λDE3 pLysS strain under ampicillin and chloramphenicol selection. pET22b-Δ9-His6x-RelB:RelE was received from Dr. Scott Strobel. Bacteria were lyzed by sonication and the lysate clarified under high-speed spin before adding the clarified lysate (~25 ml) to 5 ml Ni-NTA agarose resins (Qiagen) that was pre-washed with lysis buffer (50 mM Tris-HCl, pH 8.0, 300 mM KCl, 5 mM MgCl_2_, 10 mM imidazole and 5 mM β-mercaptoethanol). The lysate-resin mix was incubated for 4 hr at 4°C before batch washing with 90 ml of lysis buffer with 20 mM imidazole. The wash was repeated before the resin was transferred into a column to elute RelE by gravity flow using elution buffer that consist of 50 mM Tris-HCl, pH 8.0, 300 mM KCl, 9 M Urea, 20 mM imidazole and 5 mM β-mercaptoethanol. Denatured RelE will elute from the column and was collected as 1 ml fractions. Fractions that contained RelE was pooled (~5 ml) and diluted 10-fold with refolding buffer (50 mM Tris-HCl, pH 8.0, 10% glycerol (v/v) and 5 mM βmercaptoethanol), which allows RelE to refold. At this point, any precipitated proteins were removed by centrifugation and the refolded RelE further purified through a 5 ml HiTrap^®^ SP HP column (GE Healthcare) pre-equilibrated with cation exchange binding buffer (50 mM Tris-HCl, pH 7.5, 50 mM KCl and 1 mM dithiothreitol). Bound RelE was washed with 10 CV (50 ml) of the binding buffer before step elution with the binding buffer that contained 1 M KCl. 5 ml fractions were collected, and the RelE-containing fractions (~10 ml) were pooled and concentrated (3000 MWCO Amicon Ultra concentrator) with buffer exchange. The final enzyme was suspended in 50 mM Tris-HCl, pH 7.5, 70 mM NH_4_Cl, 30 mM KCl, 7 mM MgCl_2_, 1 mM dithiothreitol and 20% (v/v) glycerol, aliquoted as 30 μl fractions and stored storage at −80°C.

#### Pyranose oxidase purification

Pyranose oxidase engineered to carry C-terminal (His)_6_ tag (expressed from plasmid p-PO) was expressed in Rosetta (DE3) pLysS cells under ampicillin (50 μg/ml, f.c.) and chloramphenicol (34 μg/ml, f.c.) selection.^134^ Cells were grown for 2–3 hr in fresh starter culture before inoculating into 1 L 2YT media wherein cells were permitted to grow at 37°C until the ${\text{A}}_{600}$ absorbance reached 0.8. Following which, the culture was shifted to 16°C and allowed to adapt and equilibrate to the new temperature before adding 1 mM (f.c.) IPTG (Thermo Fisher Scientific) to induce expression of pyranose oxidase for 16 hr. Cells were then harvested, washed, lyzed by sonication and clarified prior to affinity purification using HisTrap^™^ HP column (GE Healthcare). Clarified lysate containing pyranose oxidase was introduced into the column and washed for 10 column volumes (50 ml) using binding buffer (Potassium phosphate buffer (50 mM K_2_HPO_4_ and 50 mM KH_2_PO_4_), pH 6.5, 500 mM KCl and 40 mM imidazole) before it is eluted using a linear 40 mM–1 M gradient of imidazole. Fractions that harbor pyranose oxidase were pooled and concentrated using 30 kDa MWCO Amicon Ultra-15 Centrifugal Filter Units (Millipore Sigma) to 4.5 ml before diluting the concentrated sample by 10-fold with potassium phosphate buffer reducing KCl concentration to 50 mM. Next, the sample was loaded into HiTrap^®^ Q column, washed with 10 CV (50 ml) of the anion exchange buffer (50 mM Potassium phosphate buffer, pH 6.5, 50 mM KCl) and then eluted using a linear gradient from 50 mM–1 M KCl in the same buffer. Finally, fractions that contain pyranose oxidase were pooled, concentrated, aliquoted into 50 μl fractions, flash-frozen with liquid nitrogen and stored at −80°C.

#### Quantify pyranose oxidase activity

Pyranose oxidase converts molecular O_2_ into H_2_O_2_, which can be coupled to the peroxidase-mediated oxidation of Azino-bis(3-Ethylbenzthiazoline-6-Sulfonic Acid) (ABTS) as a proxy to quantify the activity of the enzyme. Oxidized ABTS absorbed strongly at ${\text{A}}_{405}$, which we monitored with time to obtain the rate of H_2_O_2_ production and hence the activity of our purified pyranose oxidase. In 100 μl volume, we added 1 μl of 10-fold diluted pyranose oxidase to 100 μmol ABTS (Pierce), 6 U horseradish peroxidase (Pierce), 5 μmol glucose (Sigma) in 1X polymix buffer (20 mM HEPES, pH 7.5, 95 mM KCl, 5 mM Mg(OAc)_2_, 5 mM NH_4_Cl, 0.5 mM CaCl_2_, 1 mM spermidine, 8 mM putrescine and 1 mM DTT) to kickstart the reaction and recorded the absorbance at ${\text{A}}_{405}$ nm every 30 sec for 8 min at 25°C. The reaction consisted of a lag followed by a linear rate of increase in the absorbance at 0.01 s^−1^. By using the molar extinction coefficient of ABTS (36800 M^−1^s^−1^) and by correcting for the 10-fold dilution factor, the 1 mm pathlength and the reaction volume, we obtained an activity rate for pyranose oxidase in producing H_2_O_2_ required to oxidize ABTS of 0.18 μmolmin^−1^. Given the definition that 1 unit (U) is defined as the amount of enzyme needed to oxidize 2 μmol of ABTS, per min at 25°C, we determined that our purified *T. multicolor* pyranose oxidase stock has an enzymatic activity of 0.09 Uμl^−1^.

#### GreA and GreB purification

The coding sequences of GreA and GreB amplified from bacteria strain MG1655 were cloned into pET2Bc-T vectors to give plasmids pET2Bc-T-GreA and pET2Bc-T-GreB, which were transfected into BL21 λDE3 strains. Upon IPTG (Thermo Fisher Scientific) induction, the C-termini (His)_6_-tagged GreA and GreB proteins were harvested by sonication in lysis buffer (100 mM Tris-HCl, pH 8.0, 200 mM KCl, 40 mM imidazole, 5% (v/v) glycerol and 1 mM DTT). As before, the lysates were clarified under high-speed spin before the first purification with HisTrap^™^ HP columns (GE Healthcare). The captured Gre proteins were eluted from the column in a linear gradient of 40 mM–1 M imidazole in IMAC buffer (50 mM Tris-HCl, pH 8.0, 1 M KCl, 0.1 mM EDTA, 5% (v/v) glycerol and 1 mM DTT). Next, we pooled fractions that contained the Gre proteins and cleaved off their C-termini (His)_6_-tags with 2.6 μM (f.c) TEV protease overnight at 4°C. Gre proteins that had their (His)_6_-tags successfully removed were further separated from the uncleaved population and TEV enzymes by reverse His purification using IMAC buffer with 40 mM imidazole. The cleaved Gre proteins were concentrated and subjected to the final purification by size exclusion chromatography using HiPrep^™^ Sephacryl S100 16/60 column (GE Healthcare) in 10 mM Tris-HCl, pH 8.0, 1 M KCl, 1 mM EDTA, 5% (v/v) glycerol and 1 mM DTT. Finally, fractions that contain the Gre proteins were pooled, concentrated, aliquoted into 20 μl fractions, flash-frozen with liquid nitrogen and stored at −80°C.

#### Express and purify biotinylated *E. coli* RNAP

RNAP was expressed in BL21 transfected with plasmid pIA1234, which in turn was derived from pIA787 that codes for the α, β and ${\text{β}}^{\prime }$ subunits of *E. coli*.^135^ In pIA1234, the C-terminus of the ${\text{β}}^{\prime }$ subunit was modified to carry the esrtA recognition sequence (LPETG) followed by a hexahistidine tag. Plasmid pIA1234 was kindly gifted to us by Dr. Irina Artsimovitch. To purify RNAP, we modified the previous protocol slightly.^135^ First, we auto-induced the expression of RNAP in BL21, harvested and pelleted the cells, which were then resuspended in lysis buffer (50 mM Tris-HCl, pH 6.9, 500 mM NaCl, 5% glycerol (v/v)) supplemented with cOmplete^™^, EDTA-free protease inhibitor (Roche) and lysed by sonication (power setting of 8, 20 × 10 sec pulse with 1 min cooling intermission). The cell lysate was clarified by two rounds of centrifugation at 15,000 rpm for 30 min in JA-20 rotor pre-chilled to 4°C. Soluble RNAP in the supernatant was loaded onto HisTrap^™^ HP column (GE Healthcare) with IMAC binding buffer (lysis buffer + 20 mM imidazole), washed with 10 column volumes of lysis buffer (50 ml) and eluted as 1 ml fraction using a linear 20 mM–250 mM imidazole gradient over 4 column volumes. Fractions containing RNAP were pooled, concentrated and buffer-exchanged three times into esrtA reaction buffer (300 mM Tris-HCl, pH 7.5, 150 mM NaCl and 5 mM CaCl_2_). Following which, RNAP was biotinylated in a transpeptidase reaction that contained, as final concentrations, 193 μM RNAP, 36 μM esrtA, and 2.4 mM biotinylated synthetic peptide (GGGGDGDYK-Biotin, GenScript) for 30 min at 37°C.^131,136,137^ Upon successful transpeptidase reaction, the hexahistidine tag of RNAP will be exchanged for the biotin tag thereby allowing us to remove unbiotinylated RNAP by reverse His purification. Biotinylated RNAP that flowed right though the IMAC column was diluted with no salt Buffer A (50 mM Tris-HCl, pH 6.9, 0.5 mM EDTA, 5% glycerol (v/v) and 1 mM DTT) prior to further purification on a HiTrap^®^ Heparin HP column (GE Healthcare). The loaded heparin column was washed with 10 column volumes of Buffer A and the biotinylated RNAP was eluted from the column using a linear gradient of 0–100% high salt Buffer B (Buffer A + 1.5 M (f.c.) NaCl) as 5 ml fractions across 12 column volumes. Fractions containing biotinylated RNAP were pooled and dialyzed overnight at 4°C in low salt buffer (50 mM Tris-HCl, pH 6.9, 75 mM KCl, 0.5 mM EDTA, 5% glycerol (v/v) and 1 mM DTT) before loading on a Mono Q^®^ 5/50 column (GE Healthcare). Here, biotinylated RNAP enriched on the anion exchange column was washed with 10 column volumes of 5% Buffer B and eluted using a 5–100% linear Buffer B gradient. Peak fractions were pooled and dialyzed into storage buffer (10 mM Tris-HCl, pH 7.5, 100 mM KCl, 0.1 mM EDTA, 0.1 mM DTT, 50% glycerol (v/v)) overnight at 4°C using 3.5 kDa MWCO Spectra/Por 3^®^ reconstituted cellulose dialysis membrane (Repligen). Biotinylated RNAP was aliquoted, flash frozen and stored at −80°C.

#### S100 lysate purification

From 8 g of MRE600 cell pellet that was rinsed and suspended in 15 ml of Buffer 1 (10 mM Tris-HCl, pH 7.5, 30 mM NH_4_Cl, 10 mM MgCl_2_ and 6 mM β-mercaptoethanol), we lyzed the cells by sonication (power setting of 9, 15 × 10 sec pulse with 40 sec cooling intermission). The lysate was clarified twice at 30,000*g* (16000 rpm in JA-20 rotor) for 30 min at 4°C. Clarified lysate was transferred to a clean tube and spun with Ti70 rotor (Beckman Coulter Life Sciences) at 53,500 rpm for 2 hr at 4°C. Following the spin, we harvested the upper 2/3 of the supernatant (~17 ml) into a new tube, topped up the tube with Buffer 1 and re-spun the sample with the same settings. Likewise, after the spin, the upper 2/3 of the supernatant was carefully removed while avoiding the ribosome pellet. Next, we passed the supernatant through a 5 ml HiTrap^®^ DEAE column (GE Healthcare) pre-rinsed with 5 CV (25 ml) of Buffer 1. The column was then washed with 10 CV (50 ml) of Buffer 1 and eluted with Buffer 1 containing 250 mM NH_4_Cl. During the elution, 1.5 ml fractions were collected, and we pooled fractions 4–7 (6 ml; yellowish in color) for dialysis using 3.5 kDa MWCO Spectra/Por 3^®^ reconstituted cellulose dialysis membrane (Repligen) in Buffer 1 supplemented with 60 mM NH_4_Cl. A first 4 hr and a subsequent overnight dialysis were conducted at 4°C. After dialysis, the 6 ml S100 lysate was concentrated with 3 kDa MWCO Amicon Ultra-15 Centrifugal Filter Units (Millipore Sigma) into 1 ml, aliquoted as 25 μl fractions and stored at −80°C.

#### Express, purify and label initiator tRNA^fMet^

Two complementary DNA ultramers (CBD389 and CBD390) bearing the sequence for tRNA^fMet^ isoform 1 were annealed and inserted into tRNA^Met^ scaffold plasmid pBSM between XhoI and PstI sites.^138–140^ The ensuing plasmid pBSM-lpp-tRNA^fMet^ was transformed into HB101 strains for expression under the strong lipoprotein promoter (*lpp*).^141^ Briefly, 1 ml of HB101 overnight culture was inoculated into 4 L of terrific broth with 200 μg/ml ampicillin and grown at 37°C until ${\text{A}}_{600}$ = 0.5. Cells were harvested, washed once with 1X PBS (10 mM Na_2_HPO_4_, 1.8 mM KH_2_PO_4_, 137 mM NaCl and 2.7 mM KCl) and pelleted. Every 2 g of cell pellet was suspended in 15 ml of lysis buffer (10 mM Tris-HCl, pH 6.0, 10 mM MgCl_2_ and 50% phenol). The cell suspension was constantly mixed for 45 min at 4°C to ensure complete cell lysis prior to phenol-chloroform extraction (twice with phenol, once with phenol:chloroform:isoamyl alcohol (25:24:1) and once with chloroform). To the extracted aqueous phase, NaCl was added to a final concentration of 2 M to precipitate and then to remove ribosomal RNA by centrifugation at 20,000 rpm for 30 min at 4°C using JA-20 rotor. Next, we ethanolprecipitated tRNA, the majority being tRNA^fMet^, by adding to the supernatant 200-proof 100% ethanol that is three times its volume. The tRNA^fMet^ was pelleted, washed once with 70% ethanol, air-dried, and suspended in 1 ml distilled water. We further purified the initiator tRNA^fMet^ by pasing it through a 5 ml HiTrap^®^ DEAE Sepharose FF column (GE Healthcare) that has been pre-equilibrated with DEAE buffer (20 mM Tris-HCl, pH 7.5, 50 mM NaCl and 8 mM MgCl_2_). The tRNA^fMet^-bound column was washed with DEAE buffer for 10 column volumes (50 ml) before the initiator tRNA was eluted from the column using a linear gradient from 50 mM–1 M NaCl in DEAE buffer. Fractions that absorbed strongly at ${\text{A}}_{260}$ were pooled and supplemented with 2X volume of 100% ethanol to recover tRNA^fMet^ by precipitation. The tRNA^fMet^ pellet was washed once with 70% ethanol, air-dried, and suspended in 500 μl distilled water. At this point, we obtained ~300–400 μM of tRNA^fMet^ (1 ${\text{A}}_{260}$ unit = 1500 pmole of tRNA^fMet^ in 1 ml). For the final purification, tRNA^fMet^ was ran in and cut out from 8% denaturing urea gels. Gel slices was incubated in 2X PK buffer (200 mM Tris-HCl, pH 7.5, 25 mM EDTA, 300 mM NaCl and 2% w/v sodium dodecyl sulfate) with constant mixing at room temperature overnight to extract tRNA^fMet^. The tRNA^fMet^ in 2X PK buffer was phenol-chloroform extracted, ethanol precipitated, and suspended in distilled water. After PAGE purification, we recovered ~40–50% of the input tRNA^fMet^. To fluorescently label tRNA^fMet^, we exploited its natural 4-thiouridine modification to attach Janelia Fluor^®^ 549 (JF549) dye, a kind gift from Dr. Luke Lavis, by thiol-maleimide chemistry.^142^ The JF549 maleimide dye was reconstituted as 50 mM stock in anhydrous dimethyl sulfoxide (DMSO). With slight modifications of the previously described protocols, we had in 200 μl reaction, 170 μM of commercial tRNA^fMet^ (tRNAprobes) or purified tRNA^fMet^ and 4.5 mM JF549 maleimide dye in 50 mM Tris-HCl, pH 7.5 supplemented with 1 mM Tris(2-carboxyethyl)phosphine hydrochloride (TCEP).^143,144^ After 2 hr of labeling at 50°C, the reaction was diluted into 400 μl containing 300 mM (f.c.) KOAc, pH 5.3, extracted once with acid phenol and twice with phenol:chloroform:isoamyl alcohol (25:24:1) to remove excess free dye. Separately, with 400 μl of 300 mM KOAc, residual tRNA^fMet^-JF549 was back extracted from the organic phase of phenol and phenol/chloroform. We combined and recovered tRNA^fMet^-JF549 from all extractions by ethanol precipitation. To further separate tRNA^fMet^-JF549 from unlabeled tRNA^fMet^ and residual JF549 dye, we subjected tRNA^fMet^-JF549 to high-performance liquid chromatography using Xbridge^™^ BEH C18 column (Waters^™^) attached to 1260 Infinity HPLC system (Agilent). To avoid saturating the column, we injected in 5 μl volume 1500 pmol of tRNA^fMet^-JF549 into the C18 column for each round of purification. At a flow rate of 1 ml/min using a linear gradient from 5–15% ethanol in 20 mM ammonium acetate (pH 5.0), 10 mM MgCl_2_, 400 mM NaCl and over a duration of 25 min, we collected fifty 500 μl fractions. An initial run monitors fluorescence emission at 568 nm using an excitation wavelength of 554 nm along with the absorbance readings at ${\text{A}}_{260}$ to identify fractions that contain tRNA^fMet^JF549. Given that the elution profiles of tRNA^fMet^-JF549 were highly reproducible among different runs, we monitored only the ${\text{A}}_{260}$ absorbance to avoid the photo-bleaching of the dye. Fractions containing tRNA^fMet^-JF549 across all runs were collected, pooled, and precipitated. Finally, the recovered tRNA^fMet^-JF549 was reconstituted in distilled water as 140 μM stock, which was stored at −20°C.

#### Charging tRNA^fMet^ or tRNA^fMet^-JF549

A typical 100 μl charging reaction consisted of 30 μM tRNA^fMet^ or tRNA^fMet^-JF549, 200 μM methionine, 5 μM methionyl-tRNA synthetase, 5 μM methionyl-tRNA formyltransferase (FMT), 260 μM ^10^N-formyltetrahydrofolate, 1 mM rATP and 0.8 U/μl RNaseOut^™^ in 1X charging buffer (50 mM HEPES, pH 7.5, 50 mM KCl, 10 mM MgCl_2_ and 5 mM DTT). After 30 min incubation at 37°C, we added 80 μl of distilled water and 20 μl 3 M KOAc, pH 5.3 to the reaction, extracted the reaction three times with equal volume (200 μl) of acidic phenol:chloroform:isoamyl alcohol (25:24:1) and once with chloroform. Then 600 μl of 100% ethanol was added to 200 μl of the extracted sample, incubated on ice for 1 hr. Charged tRNA was pelleted with a bench-top centrifuge under top speed at 4°C, washed once with 70% ethanol, air-dried, and re-suspended in 30 μl of 1 mM KOAc, pH 5.3. The concentration of the charged tRNA was determined by its ${\text{A}}_{260}$ absorbance (~40 μM) and the approximation that 1 ${\text{A}}_{260}$ unit is equivalent to 1500 pmol of tRNA in 1 ml.

#### Charging total tRNA

We suspended 100 mg of total tRNA from MRE600 (Roche) with 1.2 ml of 1 mM KOAc, pH 5.3, which was then aliquoted and stored at −80°C. The concentration of the total tRNA stock is ~2 mM. In a 1 ml charging reaction, we incubated 1 μmol total tRNA with 400 nmol of each amino acid (Table S1A), 5 μmol rATP, 400 U RNaseOUT^™^ and 5 μl of the S100 DEAE-purified lysate for 30 min at 37°C. After which, we performed three rounds of acidic phenol:chloroform:isoamyl alcohol (25:24:1) extraction followed by a single round of chloroform extraction. We also carried out back extraction of the organic phase with 250 μl of 1 mM KOAc, pH 5.3. To the combined extracted total charged tRNA, we added 3750 μl of 100% ethanol—that is three times its volume—to precipitate the tRNA. After 10 min of spin at 4°C at the top speed of a bench-top centrifuge, we pelleted the charged total tRNA, rinsed the pellet once with ice-cold 70% ethanol, air dried the pellet and resuspended it in 1 ml of 1 mM KOAc, pH 5.3. Next, we split the charged total tRNA into three equal fractions and purified them separately (to avoid saturating the column) using a 5 ml HiTrap^®^ desalting column to remove free contaminating rNTPs. Briefly, the column was equilibrated with 1 mM KOAc, pH 5.3 prior to the manual loading of the charged total tRNA (~333 nmol) using a 1 ml syringe. Next, 1 ml of 1 mM KOAc, pH 5.3 was introduced into the column and the eluate was discarded. Finally, 1.5 ml of 1 mM KOAc, pH 5.3 was administered wherein three 500 μl fractions were collected. These fractions contain charged total tRNA, which was precipitated with 800 μl of 100% ethanol, washed once with 70% ethanol, air-dried, and suspended in 1 mM KOAc, pH 5.3 to achieve a stock concentration of ~3 mM. To minimize repeated freeze-thawing of the charged total tRNA, the stock was aliquoted into smaller fractions and stored at −80°C.

#### Bulk biochemical experiments

##### Transcription-translation coupling reaction

Broadly, we first assembled the elongating RNAP following which the ribosome was loaded on the RNA to obtain the transcription-translation complex (TTC). By the method of step-wise bubble assembly,^79^ 2 pmol of biotinylated core RNAP with equimolar RNA (CBR27/152) pre-annealed to DNA template strand (CBD362, 77 nt) were incubated for 10 min at 37°C in polymix assembly buffer (20 mM HEPES, pH 7.5, 95 mM KCl, 4 mM Mg(OAc)_2_, 5 mM NH_4_Cl, 0.5 mM CaCl_2_, 1 mM spermidine, 8 mM putrescine and 1 mM DTT). Next, 2 pmol of DNA non-template strand (CBD340, 68 nt) was added and incubated for an additional 10 min at 37°C to complete the construction of the elongating RNAP. The 1.5 μM stock of pre-annealed RNA-DNA template strand hybrid was formed in polymix annealing buffer (20 mM HEPES, pH 7.5, 95 mM KCl, 2 mM Mg(OAc)_2_, 5 mM NH_4_Cl, 0.5 mM CaCl_2_, 1 mM spermidine, 8 mM putrescine and 1 mM DTT) on a thermocycler with the following protocol: 5 min at 45°C, 2 min at each temperature from 43°C–27°C in 2°C step and 10 min at 25°C for a total duration of 33 min. The template and non-template strands were kept short—contain only partial *PyrBI* leader sequence—to discourage excessive intramolecular interaction that can reduce the efficiency of bubble assembly. Next, 3.3 pmol of ^32^P α-ATP (PerkinElmer) was added in a final volume of 10 μl and incubated for another 5 min at 37°C so that RNAP can incorporate the radioisotope into RNA. The extension of the radiolabeled RNA served as the readout for transcription monitored by resolving the RNA in 8% urea PAGE. Pierce^™^ streptavidin-coated magnetic beads (Thermo Fisher Scientific) was present (8 mg/ml) to capture the biotinylated RNAP during bubble assembly. We performed three sequential washes of RNAP immobilized on the beads with polymix assembly buffer to remove non-incorporated components. Then, the DNA duplex of the bubble complex was extended to reconstitute the full *PyrBI* leader sequence with 2 pmol of pre-annealed DNA duplex (CBD363 and CBD364 or CBD363 and CBD602), 5 nmol rATP and 40 U T4 DNA ligase (NEB) in a 20 μl ligation reaction that was allowed to proceed for 10 min at 25°C. The bubble complex was again washed with polymix assembly buffer to remove the non-ligated duplexes. In the case of non-biotinylated RNAP, we used anti-α antibody (BioLegend) to tether the transcription bubble to Dynabeads^™^ Protein G supramagnetic beads (Thermo Fisher Scientific). To assemble the ribosome on the RNA of the transcription bubble, 10 pmol of ribosome, 10 pmol of fMet-tRNA^fMet^, 20 pmol each of IF1, IF2 and IF3 and 100 pmol of rGTP were added to the elongating RNAP in a final volume of 10 μl in polymix assembly buffer and incubated at 37°C for 10 min. After which, excess ribosomes and translation initiation factors were washed off using polymix assembly buffer. The final transcription-translation reaction contained the assembled TTC in polymix buffer with rNTPs, factor mix and the rNTPs start mix (Tables S1A–S1F). The polymix buffer with rNTPs also contained creatine phosphate, amino acids, uncharged total tRNA from MRE600, rATP and rGTP (Table S1C). We adjusted the concentration of magnesium to account for its sequestration by rNTPs rNTPs thus ensuring that its free concentration was kept at 5 mM (Table S1C). To allow for translation, we added to the reaction 10X factor mix (diluted from a 100X stock with factor mix dilution buffer), which contained all 20 amino acid tRNA synthetases, EF-G, EF-Tu, EF-Ts, creatine phosphokinase, myokinase, pyrophosphatase and nucleotide diphosphate kinase (Tables S1D and S1E). To activate transcription, 2 mM (f.c.) rCTP and 5 μM (f.c.) rUTP were added to the reaction (Table S1F). For −coupling reaction, the amino acids were left out to deprive the ribosome of its substrates (amino-acylated tRNAs) for translation elongation. In reactions with ribosome inhibitors (chloramphenicol, fusidic acid, tetracycline and cycloheximide), 500 μM (f.c) of each drug was included.

###### Effects observed during coupling requires active translation

To test the function of the ribosomes in transcription-translation coupling, we added translation elongation inhibitors (tetracycline, chloramphenicol, fusidic acid, and cycloheximide) to +coupling reactions. Tetracycline competes with the binding of the aminoacyl-tRNA•EF-Tu•GTP ternary complex to the ribosome, chloramphenicol binds to the peptidyl transferase center at the ribosomal A-site and inhibits the peptidyl transferase reaction while fusidic acid prevents the release and turnover of EF-G•GDP complex on the ribosome and blocks the backward rotation of the head of the small ribosomal subunit.^90,145^ These prokaryotic-specific ribosome inhibitors effectively eliminates the transcription rate enhancing, as well as the HP site pausing- and termination-suppressing activities observed during coupling (Figures S2D–S2K; Tables S2A and S2B). By contrast, cycloheximide, a eukaryotic-specific ribosome inhibitor did not suppress the effects observed during coupling (Table S2B). We do notice, however, that tetracycline, chloramphenicol and fusidic acid were only partially effective at neutralizing the effect of coupling on reducing the efficiency and duration of pausing at P1 (Figures S2D–S2F; Tables S2A and S2B). We ascribe this observation to the reversible dissociation of these translation inhibitors,^146^ coupled with the proximity of P1 to the TTC assembly site whereby RNAP has less chance of traveling far enough for the ribosome to not influence its activity. Taken together, our results indicate that an actively translocating ribosome is responsible for the transcription promoting effects observed in the +coupling reaction.

#### Transcription fidelity assay

The elongating RNAP was assembled using the bubble method with equimolar quantities of CBR27/384 RNA (2.1 pmol) pre-annealed to template DNA (CBD394 or CBD451) complete with non-template DNA (CBD393 or CBD450) in the presence of 80 μg of streptavidin magnetic beads (Pierce) in 10 μl volume for a total duration of 20 min at 37°C. Internal labeling of RNA ensued for 5 min at 37°C with 3.3 pmol of ^32^P-α-ATP (PerkinElmer). The bead-bound bubble complex was washed three times with 1X polymix buffer (4 mM Mg(OAc)_2_) before we assembled the ribosome on the RNA to construct TTC for the +coupling reaction. To assemble TTC, we added 10 pmol ribosome, 10 pmol fMet-tRNA^fMet^, 20 pmol each of IF1, IF2 and IF3 and 200 pmol rGTP in 1X polymix buffer (5 mM Mg(OAc)_2_) to the elongating RNAP complex in a 10 μl reaction and incubated for 10 min at 37°C. For the −coupling reaction, the same TTC assembly was performed except that we replaced the ribosome with the gradient buffer. Following which, the assembled RNAP (−coupling) or TTC (+coupling) was washed three times with 1X polymix buffer (4 mM Mg(OAc)_2_) before constituting the enzymes in the final reaction mix containing pre-charged tRNA in polymix buffer, translation elongation mix and the rNTPs start mix (Tables S1L–S1O). We introduced the rNTP start mix (2 mM rATP, 2 mM rGTP and 1 mM rUTP, f.c.) last to initiate transcription (Table S1O). The DNA template strand harbors sequences from the λPR promoter to test misincorporations of rNTPs at six positions (56, 59, 61, 66, 67 and 68) specifying for cytosine where the numbers correspond to the length of the RNAs at those positions. Reactions were quenched in formamide loading dye at multiple time points and the RNAs analyzed in 12% denaturing urea polyacrylamide gel. In reactions that include GreA or GreB, 2 μM of the enzymes (f.c.) were used.

#### Exonuclease III mapping of RNAP boundaries

For downstream boundary mapping of RNAP that sits 1 nt upstream of P1 (RNAP-U9), we incubated 6 pmol of biotinylated core RNAP with 3 pmol of 5′ FAM-labeled RNA (CBR26) pre-annealed to 3 pmol of template strand (CBD627) in polymix buffer (4 mM Mg(OAc)_2_, f.c.) at 37°C for 10 min. Next, 3 pmol of 5′ Cy5-labeled non-template strand (CBD626) was added to a final volume of 10 μl and incubated at 37°C for an additional 10 min. The template strand carries 3 terminal phosphorothioate modifications that protects its 3′ end from digestion by exonuclease III (ExoIII) thus forcing digestion by ExoIII to occur only on the non-template strand in a 3′ to 5′ direction (Figure S1A). The same assembly protocol was employed for upstream boundary mapping construct except that 5′ FAM labeled RNA was annealed to 5′ Cy3-labeled template strand (CBD629) along with its complementary phosphorothioate-bearing non-template strand (CBD628). Adding rATP (10 μM, f.c.) to RNAP-U9 yielded RNAP at P1 (RNAP-A10). Unassembled oligonucleotides, rATP and RNAP were removed in 5 washes, each using 20 μl of polymix buffer before the start of ExoIII digestion. Boundary mapping of RNAP was conducted in polymix buffer (5 mM Mg(OAc)_2_, f.c.) initiated with 80 units of ExoIII in 20 μl reaction at 37°C. After initiating the digestion reaction, 2 μl of the reaction was quenched at various timepoints in 8 μl of quenched mix (98% deionized formamide, 10 mM EDTA, pH 8.0). Quenched samples were heated at 95°C for 3 min before loading onto a 15% denaturing urea gel that had been pre-run for at least 30 min at 10W. On a separate lane, quenched mix with dye (0.025% (w/v) xylene cyanol and 0.025% (w/v) bromophenol blue) was loaded as dye front references given that the dyes were left out in the sample lanes as they interfere with the fluorescent signals. Gels were scanned on Typhoon FLA 9500 where the FAM-labeled RNA was detected using 473 nm laser coupled with the LPB(510LP) filter, the Cy5-labeled non-template strand was perceived using 635 nm laser coupled with the LPR(655 LP) filter and the Cy3-labeled template strand was imaged using 532 nm laser coupled with the LPG(575LP) filter. Scans were performed at 50 μm resolution with PMT settings of 800 V for Cy3 and Cy5 and 700 V for FAM.

##### Mapping the translocation register of RNAP at P1

We want to first highlight that P1 could have emerged due a single A-T base pair insertion into the endogenous sequence at a site close to P1 to maintain the reading frame of the *PyrL* gene. Thus, we make no inference about the physiological relevance of P1 except to use it as a readout to investigate how the ribosome affects RNAP pausing.

A post-translocated RNAP has an opening in the insertion site (also known as the *i+1* or the A site) for the incoming ribonucleotide, which during catalysis will be added to the 3′ end of the RNA. The insertion site which was previously empty, is now occupied by the newly acquired ribonucleotide and the polymerase is now said to be in a pre-translocated state (Figure S1A). Upon the downstream translocation of RNAP by 1 base pair, the insertion site is once again vacated, and the polymerase reassumes its ribonucleotide-receptive post-translocated state primed for the next ribonucleotide addition cycle (Figure S1A). Instead, if RNAP forward translocate by 2 base pair, it goes into a hyper-translocated state^85^ (Figure S1A). Occasionally, RNAP can also slide backwards into a backtracked state.^147^ Much like in a pre-translocated state, RNAP that resides in a backtracked or in a hyper-translocated state is incapable of binding to incoming ribonucleotide.

We mapped the position of P1 using a sequencing urea gel and found that it corresponds to a 96 nt RNA, that results from the pausing of RNAP prior to the addition of an rUTP (Figure 1D). The low concentration of rUTP in our assay (5 μM), however, does not entirely explain the strong pause at P1. For instance, positions 57 and 58 on the DNA template specifying tandem adenines resulted in much weaker band intensities and hence pause strengths. Therefore, the sequence context at P1 must have contributed to the strong transcriptional pausing. Indeed, sequences at P1 partially resemble a previously identified pause consensus element G_−11_G_−10_Y_−1_G_+1_, commonly found near the Shine-Dalgarno motif, in which −1 corresponds to the 3′ terminal sequence of the RNA, which often is a pyrimidine (Y)^148,149^ (Figure S1B). This pause element traps RNAP in its pre-translocated (ribonucleotide-nonreceptive) state.^148,149^ By contrast, the RNA at P1 bears an adenine at –1. Nonetheless, P1 conforms to the G_−11_ and G_−10_ consensus (Figure S1B), whereby these two strong upstream rG-dC hybrid base pairs may be sufficient to hinder the transition of the enzyme into the post-translocated (ribonucleotide-receptive) state^150^ (Figure S1B).

To understand the mechanism of transcriptional pausing associated with P1 in relation to the translocation register of RNAP, we mapped both the upstream and the downstream boundaries of the polymerase on DNA by ExoIII digestion (Figure S1C). Concurrently, we tracked transcription with RNA labeled at its 5′ end with a FAM dye. Digestion of the Cy3-labeled template strand by ExoIII defines the upstream boundary of RNAP, whereas digestion of the Cy5-labeled non-template strand determines the downstream boundary of RNAP (Figure S1A). By comparing these complementary boundaries information of RNAP at P1 (RNAP-A10) to RNAP that is one base pair upstream of P1 (RNAP-U9) as our reference, we showed that RNAP-A10 assumes preferentially a hyper-translocated register in contrast to RNAP-U9, which adopts predominantly a post-translocated register.

##### RNAP-U9 is preferentially post-translocated

We began our mapping analysis with RNAP that sits one base pair upstream of P1 (RNAP-U9) with a 17 nt RNA (Figures S1A and S1D). In upstream border mapping of RNAP-U9, ExoIII digestion of the Cy3-labeled template strand from the 3′ to 5′ direction produces DNA fragment that is predicted to be 47 nt (for pre-translocated RNAP), 46 nt (for post-translocated RNAP) and 45 nt (for hyper-translocated RNAP) (Figure S1A). By contrast, downstream border mapping by ExoIII digestion of the Cy5-labeled non-template strand will generate the expected fragment size of 48 nt (for pre-translocated RNAP), 49 nt (for post-translocated RNAP) and 50 nt (for hyper-translocated RNAP) (Figure S1A). Our results from upstream border mapping showed that by two minutes, the bulk of the 46T fragment indicative of a post-translocated polymerase was digested by ExoIII into the 45C fragment that corresponds to a hyper-translocated polymerase (Figure S1D, first panel). A result indicating that RNAP-U9 can enter the hyper-translocated state as was previously reported.^85^ It should be noted, however, that the persistence of the band at 45C does not necessarily indicate that RNAP-U9 remains hyper-translocated. The highly dynamic RNAP is likely to oscillate between the pre-, post- and hyper-translocated states,^151^ and its return from the hyper-translocated to the post- or pre-translocated state will not be observed by DNA that was irreversibly digested by ExoIII during upstream border mapping. This is a drawback of the ExoIII mapping technique, which marks the furthest excursion of RNAP to either the most upstream (backtracked) or downstream (hyper-translocated) register. Fortunately, downstream border mapping under the same condition, provides the complementary information and demonstrates that RNAP adopts preferentially a post-translocated register. This can be seen by the major and persistent band at 49A (indicative of a post-translocated register) with a minor band at 48G, which suggests a slight excursion of the polymerase into the pre-translocated register by the end of the time course (Figure S1D, first panel).

##### RNAP-A10 is preferentially hyper-translocated

The addition of rATP moves RNAP-U9 to P1 to form RNAP-A10 with a corresponding extension of the RNA from 17 nt to 18 nt (Figure S1D, second panel). At P1, the predicted Cy3-labeled DNA fragments are 46 nt (for pre-translocated RNAP), 45 nt (for post-translocated RNAP) and 44 nt (for hyper-translocated RNAP) in upstream border mapping; the predicted Cy5-labeled DNA fragments are 49 nt (for pre-translocated RNAP), 50 nt (for post-translocated RNAP) and 51 nt (for hyper-translocated RNAP) for downstream border mapping (Figure S1A). We observed that RNAP-A10 acquired a hyper-translocated register much quicker than RNAP-U9, ascertained by the faster appearance of the hyper-translocated boundary at 44G during upstream border mapping (Figure S1D, second panel). In line with the notion that RNAP-A10 is preferentially hyper-translocated, downstream border mapping shows a slower transition from 51A (hyper-translocated RNAP) to 50C (post-translocated RNAP): an observation that can be explained by the persistence of RNAP-A10 in its hyper-translocated register before its underlying DNA gets irreversibly chewed up by the opportunistic ExoIII the moment the polymerase ventures into its post-translocated register (Figure S1D, second panel).

Consistent with a preference for the hyper-translocated register, we found that RNAP-A10 was much less susceptible to pyrophosphorolysis than RNAP-U9 (Figure S1E). That is because a hyper-translocated RNAP-A10 needs to take two 1 nt steps backward while a post-translocated RNAP-U9 needs to take only one step to be in the pyrophosphorolysis-prone pre-translocated register.^152^ Consequently, the initial velocity of pyrophosphorolysis for RNAP-A10 of 0.28 ± 0.01 s^−1^ is significantly lower than that for RNAP-U9 of 1.52 ± 0.6 s^−1^ (*p* = 4.7 × 10^−6^; Figure S1E). Taken together, our results demonstrate that the positional equilibrium of RNAP-A10 is biased towards the hyper-translocated register at P1 wherein the 3′ end of the RNA is shifted 1 nt upstream and away from the *i* site within the catalytic center. A prediction follows that the subsequent addition of the incoming cognate ribonucleotide would be impaired for a hyper-translocated RNAP-A10. Indeed, we observed that upon the addition of rUTP, not all RNAP-A10 transition into RNAP-U11 as evidenced by the incomplete conversion of the 18 nt RNA into the 19 nt RNA (Figure S1D, third panel). Finally, even though most RNAP-A10 has transitioned into RNAP-U11 with the addition of rUTP (Figure S1D, third panel), RNAP-U11 has very similar downstream border signature as RNAP-A10 suggesting that RNAP-U11 is unlikely to hyper-translocate and readily visits its post- and pre-translocated register. Indeed, RNAP-U11 sees a rapid transition of its downstream border at 51A for a post-translocated register to 50C for a pre-translocated register (Figure S1D, third panel). This is further supported by the complementary upstream border mapping of RNAP-U11 where there is incomplete conversion of 44G to 43G that corresponds to a transition of RNAP-U11 into the hyper-translocated register: a conversion that is often complete by the end of the time course during upstream border mapping as seen for RNAP-U9 and RNAP-A10 (Figure S1D, first, second and third panels).

Previous studies demonstrated that the binding of an incoming cognate ribonucleotide can induce the shift of a pre-translocated RNAP into its post-translocated register.^30,95,153^ By contrast, we showed that a non-hydrolysable cognate rUTP (UpNHpp) was unable to alter the preferred hyper-translocated register of RNAP at P1 into its post-translocated register (Figure S1D, fourth panel). In upstream boundary mapping, the rate of appearance of the 44G fragment is the same with or without UpNHpp (Figure S1D, compare second and fourth panel). Likewise, in downstream border mapping, we observed that the transition from 51A (hyper-translocated RNAP) to 50C (post-translocated RNAP) follows the same kinetics in the absence or presence of UpNHpp (Figure S1D, compare second and fourth panel). We reasoned that a cognate incoming ribonucleotide aids in the forward translocation of RNAP but is ineffective against hyper-translocated RNAP at P1.

#### Pyrophosphorolysis assay

We subjected the elongating RNAP (RNAP-U9 and RNAP-A10) assembled for ExoIII boundaries mapping to pyrophosphorolysis.^152^ Given that RNAP is prone to pyrophosphorolysis only in its pre-translocated register, a polymerase in a post-translocated register will be less prone to pyrophosphorolysis because it would have to take a step back before the reaction can occur. RNAP-A10, which adopt preferentially a hyper-translocated register, is presumably the most resistant towards pyrophosphorolysis because the polymerase would have to translocate two steps backwards before the reaction can occur. To test this, we initiated pyrophosphorolysis in a 20 μl reaction for either RNAP-U9 or RNAP-A10 with 500 μM potassium pyrophosphate (KPPi) in 1X polymix buffer supplemented with 5 mM CalCl_2_ and 5 mU/μl of apyrase. Reactions were conducted at 37°C and quenched at various timepoints in formamide loading dye. Quenched samples were ran in 15% denaturing urea gel, the resolved RNA were imaged on Typhoon FLA 9500 and the bands were quantified with ImageQuant TL 8.2.

#### Dinucleotide cleavage assay

Elongating RNAP and TTC were assembled using the same step-wise assembly protocol on streptavidin magnetic beads except with a different set of RNA-DNA construct. The RNA was CBR27/630, the DNA template strand was CBD635 and the non-template strand was CBD634. Following the assembly, we translocated the elongating RNAP by two steps with rGTP (10 μM, f.c.) and subsequently with 3.3 pmol of ^32^P-α-ATP (PerkinElmer) to end-label the RNA. The final 20 μl reaction contained 40 μM total amino acid, 30 μM pre-charged total tRNA, translation factor mix (Tables S1D and S1E), 2 U/μl RNaseOUT^™^ and 1 mM rGTP in 1X polymix buffer with 5 mM free magnesium. For the initial test of ribonucleotide misincorporation, we used a short 9 nt RNA (CBR32) to resolve and differentiate RNA with or without misincorporated rNTP using high percentage denaturing urea gel (Figure S5A). Adding pre-charged total tRNAs and saturating rGTP initiated the reactions forcing RNAP to preferentially misincorporate at 37°C (Figures S5B and S5C). Reactions from −coupling and +coupling reactions were quenched in formamide loading dye at multiple time points and the radiolabeled RNAs and dinucleotide fragments resolved in 20% denaturing urea polyacrylamide gel.

#### Optical tweezers experiments

##### Functionalize polystyrene beads.

We coated carboxyl polystyrene beads (Bangs Laboratories, Inc.) with DNA duplexes (oligo-beads) carrying 4 nt overhangs (5′-CGAT-3′) that are complementary to the sticky ends (5′-ATCG-3′) of DNA handles resulting from restriction digest with BsaI-HF^®^ (NEB). To prepare the DNA duplex, 1000 pmol of a 5′ amino-modified oligonucleotide (CBD139; 21 nt) was annealed to an equimolar of its complementary 5′ phosphorylated oligonucleotide (CBD140; 25 nt) in 20 μl of 100 mM MES buffer, pH 4.5 and 100 mM KCl on a thermocycler with temperature ramp from 95°C to 4°C at a rate of 0.1°Cs^−1^. Separately, 5 μl of 10% (w/v) 1 μm (diameter) carboxyl-functionalized polystyrene beads (Bangs Laboratories, Inc.) was rinsed twice with 88 μl distilled water and three times with 100 mM MES buffer, pH 4.5. Following each rinse, we pelleted the beads by centrifugation at 5000 *g* for 1–2 min at 4°C and aspirated the wash buffer. After the final wash, 20 μl of the annealed duplex (50 μM) was added to the beads and 3 μl of freshly prepared 2 M 1-ethyl-3-(3-dimethylaminopropyl)carbodiimide hydrochloride (EDC, Thermo Fisher Scientific) was introduced to initiate carboxyl-to-amine crosslinking reaction between the DNA duplexes and the beads for 2–3 hr at 20°C with constant mixing in a thermomixer (Eppendorf). After which, we supplemented the reaction with 5 μl of 2 M EDC and crosslinking was allowed to continue overnight at 20°C. The following day, we pelleted the oligo-beads, removed unreacted reagents, and quenched the reaction with 88 μl 50 mM glycine, 0.02% Tween 20. The oligo-beads were then rinsed twice with the quenching buffer followed by two successive washes with storage buffer (30 mM HEPES, pH 7.5, 100 mM KOAc, 0.02% Tween 20). Finally, we suspended the oligo-beads in 20 μl of storage buffer (~2.5% (w/v)), aliquoted as 2.5 μl fractions and stored at −80°C.

#### DNA handles

DNA handles (~1.5 kb) that link RNAP or TTC to polystyrene beads held in optical traps were prepared by PCR using either lambda phage DNA or pUC19-*pyrL*-TC4 as template (NEB) with Phusion^®^ High-Fidelity DNA Polymerase (NEB). Embedded in the sequences of primers (CBD141, CBD171, CBD335 and CBD357) are BsaI restriction sites that will generate the corresponding overhangs for ligation either to oligo-beads or to transcription bubble when cut. The primer (CBD202) can also introduce a 5′ biotin to DNA handle that will capture biotinylated RNAP through a biotin-neutravidin-biotin bridge. For the transcription termination study, the primer (CBD360) carries sequences complementary to the nascent RNA of the transcription bubble. All DNA handles are cleaned up using Econospin columns (Epoch Life Science) after PCR. For DNA handles that were digested by BsaI-HF^®^, they were gel and column purified.

#### Assemble RNAP and TTC

We monitored transcription by RNAP or TTC using an optical tweezers via a decrease (opposing force) or an increase (assisting force) in the contour length of the DNA handles attached to polystyrene beads held in optical traps under constant force (Figures 3A and 5C). The stepwise assembly of an elongating RNAP requires 0.85 pmol of biotinylated RNAP, 0.75 pmol each of non-template DNA (CBD355) and RNA (CBR27/152)-template DNA (CBD356) hybrid in 5 μl reaction with 1X polymix buffer (4 mM Mg(OAc)_2_, f.c.) for a total duration of 20 min at 37°C. The assembly of TTC involves an additional step using ~13-fold excess of ribosome (10 pmol) over RNA, 5 pmol of fMet-tRNA^fMet^-JF549, 10 pmol each of initiation factors 1, 2 and 3 and 200 pmol of rGTP in a final volume of 10 μl (include the 5 μl assembly reaction for elongating RNAP) and incubated for 10 min at 37°C. Once annealed, the non-template and template DNA duplex of the bubble complex (elongating RNAP or TTC) will carry at its upstream end a 5′-CAGC-3′ overhang and at its downstream end a 5′-TGTC-3′ overhang whereby each can be ligated to additional pieces of DNA depending on the tweezing geometry. For the opposing force geometry, the downstream end of the bubble duplex is ligated to DNA handle, which links the elongating RNAP or TTC to oligo-beads and serves as the transcription template. The 10 μl ligation reaction (30 min at 25°C) consisted of 0.025% 1 μm oligo-beads, 3.75 nM bubble complex or TTC, 0.5 mM rATP, 5 nM DNA handle (CBD171/335; 1788 bp) and 2 U/μl T4 DNA ligase (NEB) in 1X polymix buffer with 4 mM Mg(OAc)_2_. In the case of the assisting force geometry, both the upstream and the downstream ends of the bubble duplex were ligated to 3.75 nM of CBD141/357 (1515 bp) and 5 nM of CBD171/358 (1500 bp) respectively. Here, the upstream DNA handle attaches the elongating RNAP or TTC to oligo-beads while the downstream DNA contains the transcription template. Separately, we assembled a 10 μl ligation reaction (30 min at 25°C) that composed of 0.025% 1 μm oligo-bead, 2.5 nM CBD141/202 (1479 bp biotinylated DNA handle), 0.5 mM rATP, 2 U/μl T4 DNA ligase (NEB) and 150 nM neutravidin (Thermo Fisher Scientific) in 1X polymix buffer with 4 mM Mg(OAc)_2_. This bead-bound DNA handle capped by neutravidin will be used to fish for the biotinylated RNAP and form a tether in the tweezing chamber. All ligation reactions were diluted into 1 ml polymix tweezing buffer (polymix buffer containing 5 mM Mg(OAc)_2_ supplemented with 12.5 mM ascorbic acid to scavenge free radicals, 2 mM (±)-6-Hydroxy-2,5,7,8tetramethylchromane-2-carboxylic acid (Trolox) to eliminate blinking of fluorophore and 0.8% (w/v) glucose) and kept on ice.^154,155^ We prepared Trolox as a 4 mM stock (50 ml) with distilled water and neutralized its pH with 250 μl of 1 M KOH. The Trolox stock was kept in the dark at 4°C. On the other hand, ascorbic acid was prepared fresh for every experiment as a 100X stock (1.25 M in 1.25 M KOH). The tweezing chamber was rinsed with polymix tweezing buffer before beads with the fishing handle were introduced into the top channel whereas beads with the elongating RNAP or TTC were loaded into the bottom channel of the chamber (Figures 3A and 5C). These sample beads will make their way into the center channel of the trapping chamber via distinct dispenser tubes and were immobilized sequentially within two separate optical traps under reduced power (20% of experimental power intensity). Following which, we moved both beads stably held by the optical traps to the experimental position in the center channel to form a tether. To this end, the bead with the fishing handle was steered towards the bead with RNAP or TTC to allow the neutravidin-capped fishing handle to capture the biotinylated RNAP. Once the tether is formed, the elongating RNAP or TTC will be positioned in the middle of the two optical traps separated by ~3000 bp of DNA handles. Then, we initiate translation and/or transcription by flowing in the coupling mix (Tables S1G–S1K) via a shunt into the main experimental channel. The polymix tweezing buffer, rNTP mix and coupling mix were filter sterilized with PVDF syringe filter (Genesee Scientific), Steriflip (Thermo Fisher Scientific) or Millex-GV Filter (Millipore) to remove particulates that will crash into our optically trapped samples.

#### Instrumentation

Data were collected on a dual trap time-shared optical tweezer that combines force spectroscopy and fluorescence measurement as was previously described.^156^ In brief, the traps were generated from Nd:YAG 1064 nm lasers focused through high-numerical aperture objectives that can stably capture polystyrene beads and measure their positions and the force applied. The two traps originate from the same laser source and are interlaced with a green laser at 200 kHz by an acousto-optic modulator, which in turn is controlled by a custom-made radio frequency board. Since the two traps and green laser are separated in time—each on for 5 μs alternating and hence timeshared—the detection of the bead positions by the trapping lasers can be determined using the same quadrant photodiode whereas the florescence signal that arises from the green laser excitation is detected using an avalanche photodiode. A LabVIEW custom interface was used to collect trap data at an acquisition rate of 1333 Hz for saving, and fluorescence counts with 10 ms binning. For each bead pair, a power spectrum analysis was used to determine the trap stiffness (average 0.2 pNnm^−1^) and an offset correction accounted for bead-to-bead interference when they are in proximity. The measured distance between the two trapped beads (nm) can be converted to the length of the DNA (bp) using the extensible worm-like chain model, and changes in this contour length over time correspond to transcription of the RNAP. Data were analyzed using custom code written in Matlab R2016a (doi:10.5281/zenodo.6534021).

#### Probing transcription termination

We reasoned that if terminator hairpin cannot fold, then transcription termination cannot occur. To test this, we assembled an elongating RNAP with attachment points for DNA handles at the biotinylated C-terminus of the ${\text{β}}^{\prime }$ subunit and at the emerging RNA of RNAP. This enables us to apply mechanical force with an optical tweezers to keep the RNA unfolded. To this end, we performed the same stepwise assembly of an elongating RNAP with RNA (CBR27/152), non-template DNA (CBD355) and template DNA (CBD356). Likewise, we extended the downstream bubble sequence in a 10 μl ligation reaction with 15 nM of the DNA CBD171/358, which will complete the *pyrL* leader sequence. Additionally, we included in the same reaction 15 nM of DNA handle (CBD141/360) that contained at one end a 30 nt 5′ overhang, which will anneal to the complementary 5′ region of the RNA extending out from RNAP and at the other end the 5′-ATCG-3′ overhang, which will ligate to the oligo-beads. The same fishing handle (CBD141/202) was used to capture RNAP to form tether between the two optical traps. All channels of the tweezing chamber were pre-rinsed and equilibrated with 1X polymix buffer (5 mM Mg^2+^) supplemented with 10 mM sodium azide to scavenge radicals.^157^ Transcription was initiated with rNTP mix (0.5 mM (f.c.) each of rATP, rGTP, rCTP and rUTP in 1X polymix buffer with 7 mM Mg(OAc)_2_ and 10 mM sodium azide) under high force (15 pN) to prevent the folding of the terminator hairpin. In the force drop experiment, transcription began at a force of 15 pN, which was then rapidly dropped to 5 pN to permit refolding of the terminator hairpin.

#### Quantifying transcription restart time

The goal is to assess the duration of time it takes for RNAP, harboring a terminal rU-dG mismatch, to resume transcription as a function of force. First, we assembled the mismatched-containing RNAP with 0.85 pmol of biotinylated RNAP, 0.75 pmol of non-template DNA (CBD612) and 0.75 pmol of RNA (CBR27/605)-template DNA (CBD613) hybrid in 5 μl reaction with 1X transcription buffer without magnesium and incubated for 20 min at 37°C. To simulate the force exerted by the ribosome on RNAP, we conducted the experiment under assisting force. For this setup, we ligated 25 fmol of the bubble complex, at its upstream end, to equimolar amount of DNA handle (CBD141/357) in the presence of 5 nmol rATP and 20 U T4 DNA ligase (NEB) in 1X transcription buffer (20 mM Tris-HCl, pH 7.5, 95 mM KCl and 1 mM DTT) in a final volume of 10 μl. We initiated the ligation reaction with MgCl_2_ (4 mM, f.c.) and restricted the reaction to 10 min at 25°C to minimize hydrolysis of the offending rUTP by RNAP (Figures S5D, S5E and S5H). Following which, we promptly diluted the reaction in 1 ml of 1X transcription buffer and stored on ice prior to its loading into the tweezing chamber. Concurrently, we prepared the oligo-beads with fishing handle capped by neutravidin in a ligation reaction that span 1 hr at 25°C in 1X transcription buffer containing 4 mM MgCl_2_. After tether formation and holding RNAP at various constant force ranging from 5–15 pN, we started the recording, opened the shunt to introduce the rNTP mix (1 mM (f.c.) each of rATP, rGTP, rCTP and rUTP in 1X transcription buffer, 14 mM MgCl_2_ and 10 mM sodium azide) and monitored the resumption of transcription.

#### Determine pause free velocity and pause density

To determine the pause-free velocity (PFV) of RNAP from single-molecule optical tweezers experiment, each transcription trajectory was first divided into ‘translocating’ and ‘paused’ sections by fitting a 1 bp monotonic staircase to the data using a hidden Markov model. Any step that lasted longer than 0.5 s was classified as a pause. This was done to separate the long sections of pausing from the short sections of translocation. The pauses were counted to determine the pause density (number of pauses per bp). The sections classified as translocating were filtered using a Savitsky-Golay differentiating filter of rank 1, width 150 ms to determine the velocity at each point. These velocities were binned to a histogram, which was fitted to the sum of two Gaussians: one centered at zero to represent the remaining pauses (ones shorter than the 0.5s cutoff but longer than the width of the filter) and one at a positive velocity, to represent the PFV of moving RNAP. It should be noted that, in this analysis, we cannot distinguish between very short pauses (off-pathway states) with timescales that are comparable to the longer dwells caused by low rUTP concentrations, but assume that such ‘micro-pausing’ is rare.

#### Determining pause durations

To calculate the pause durations for P1 and the HP site, the single-molecule traces are first aligned to each other by the transcriptional stall site, where the RNAP is paused before the experiment starts. The pause durations are measured as crossing times, e.g. for P1 it is taken as the time taken to cross from 31.5 bp transcribed to 36.5 bp transcribed (a 5bp window around the location of P1). This was done because the resolution of the traces does not allow us to assign the dwell times for individual nucleotide additions. Since the exact location of the pauses is subject to systematic errors introduced by the optical tweezers setup, the pauses were located not by their theoretical position on the transcript but rather by finding peaks in the combined residence time histogram of each condition. Pause HP was found to be at 90 bp transcribed in this assay. Statistics on the termination hairpin were not included since the opposing force traces rarely make it that far in the transcript.

To analyze pausing at P1, the pause durations were fit to a sum of two exponentials ($\text{P}DF=a_{1}k_{1}\exp\left(-k_{1}t\right)+a_{2}k_{2}\exp\left(-k_{2}t\right),k_{1}>k_{2}$) by maximum likelihood estimation. From the fit parameters $a_{1},a_{2},k_{1}$, and $k_{2}$, we can extract the pause efficiency $E=\frac{a_{2}}{a_{1}+a_{2}}$ and the pause duration $\tau =\frac{1}{k_{2}}$.

#### Quantifying restart times and fitting to the Arrhenius Equation

The restart times for an RNAP at a mismatch were extracted from the single-moelcule traces by identifying by eye when the rNTPs were introduced (signaled by a change in force due to the fluid flow) to the time transcription restarted (signaled by an increase in tether length). The restart time is then the duration between these events. The force dependence of the restart times were modeled with the below equation, a piecewise continuous function consisting of an Arrhenius relation at low forces, which saturates to a constant time after a maximum force.


$$t_{\text{restart}}(F)=\{\begin{array}{cc} t_{\text{restart}}^{0\;\text{pN}}\exp(-F•dx/kT) & F<F_{\text{max}}\\ t_{\text{restart}}^{0\;\text{pN}}\exp\left(-F_{\max}•dx/kT\right) & F\geq F_{\max} \end{array}$$

The three free parameters are the zero-force restart time $t_{\text{restart}}^{0\;\text{pN}}$ distance to transition state *dx*, and the saturation force $F_{\max}$, which were found to be 105 ± 42 s, 1.0 ± 0.2 nm, and 12 ± 2 pN, respectively (90% CIs, Figure 5F). The fitting was performed as a bilinear fit to the logarithm of the restart times. The distance to transition state is interpreted as the extent of backtracking by RNAP bearing a terminal mismatch at zero force. This corresponds to 3.0 bp, which is corroborated by Gre factors cleavage of RNA in backtracked RNAP (Figures S5D and S5F). Experimentally, the restart time was taken as the start of flow, indicated by the change in X-force of the beads, to the start of transcription, signified by the increase in tether length. Both were determined by eye, with estimated errors of 10 ms for the start point and of 0.1 s for the end point. Traces that did not restart or had ambiguous restart signatures were not considered.

#### High-throughput sequencing experiments

##### Library preparation from transcription of PyrL

RNAs extracted and purified from transcription reactions without (−coupling) or with (+coupling) ribosomes were reverse transcribed into cDNAs using a reverse primer that bears three discrete sequence elements. The first element has sequence complementary to the 3′ region of the RNA for reverse transcription (RT), the second element is a unique eight nucleotides *i7* index to barcode a library and the third element contains sequences that will anneal to P7 primer for library amplification. For RT, 2 pmol of the reverse primer was pre-annealed to 14.1 fmol of purified RNA with heating at 65°C for 5 min followed by cooling on ice for 1 min. RT was carried out in 20 μl reaction volume with 10 U/μl Superscript^™^ IV (Invitrogen), 2 U/μl RNaseOUT^™^ (Invitrogen), 0.5 mM dNTPs and 5 mM DTT in 1X Superscript IV buffer at 55°C for 10 min. Then, RT was inactivated following incubation at 80°C for 10 min. Next, during second strand DNA synthesis on cDNAs, we introduced 10 random nucleotides as unique molecular identifier (UMI) tag for each resulting duplex DNA with UMI-assigning primer (CBD604). With 14 random nucleotides that afforded at least ~2.7 × 10^8^ distinct tags, we ensured that the number of distinct tags greatly exceeds (~360-fold) the targeted number of distinct cDNA species (equivalent to the distinct number of starting RNA species) to reduce the probability that two or more species would acquire the same UMI.^158^ In addition to the UMI tag, the 3′ end of the UMI-assigning primer contained sequences that base-paired specifically to the 3′ end of the cDNA (corresponding to sequences located close to the 5′ end of the RNA) and carries a 5′ sequence extension upstream of the UMI tag that serves as the binding site for forward primer in library amplification. Second strand synthesis reaction was performed in a final volume of 45 μl with 1.3 × 10^−3^ fmol cDNA, 25.5 pmol UMI-assigning primer, 1 U Phusion HotStart DNA Polymerase (Thermo Fisher Scientific) and 9 nmol dNTPs in 1X Phusion high fidelity buffer. The protocol for second strand synthesis included 1) denaturation at 98°C for 40 sec, 2) annealing at 60°C for 30 sec, 3) synthesis of the second strand at 72°C for 10 sec and 4) cooling at 4°C. After which, 3 μl of 20 U/μl exonuclease I (Thermo Fisher Scientific) were added to remove excess RT and UMI-assigning primers at 37°C for 1 hr. Then, exonuclease I was inactivated at 98°C for 5 min. At this point, we obtained the starting duplex that can now be amplified by adding to the reaction 1.0 μl of 25 μM forward and reverse primer each (0.5 μM in a final volume of 50 μl). Both primers were modified with phosphorothioate bonds at the final and penultimate positions as a precaution against unintended digestion by residual exonuclease I. The forward primer has the P5 primer sequence, 8 random nucleotides as i5 index followed by the same 5′ extension sequences of the UMI assigning primer. The reverse primer will anneal to the P7 sequence of the starting DNA duplex. The cycling protocol for PCR amplification comprised of 1) denaturation at 98°C for 10 sec, 2) annealing at 67°C for 15 sec, 3) extension of DNA at 72°C for 10 sec and whole process repeated for a total of 28 cycles. The protocol ends with a final incubation at 72°C for 8 min and cooling and storage at 4°C. The final library is purified twice using Select-a-Size DNA Clean & Concentrator^™^ MagBead Kit (Zymo Research). Briefly, 40 μl of reconstituted MagBead was added to 50 μl of PCR reaction for the clean-up and left sided size selection to remove primers and to isolate the amplified library (307 bp). A total of two libraries (RNAs isolated from minus coupling and plus coupling reactions) were prepared separately and each library with its unique *i5* and *i7* index sequences. The concentrations and purities of the amplified libraries were determined by fragment analysis.

#### Pre-adenylation of DNA adaptor

Pre-adenylated DNA (IDT) functions as the 3′ adaptor in the construction of high-throughput sequencing libraries using RNAs extracted from transcription reaction with (+coupling) and without (−coupling) the ribosome (Figure 4E). A 36 nucleotide DNA (CBD610) that is 5′ phosphorylated and modified at the 3′ end with dideoxy cytosine serves as the substrate for pre-adenylation. In a 20 μl reaction, 6 μM of CBD610 was pre-adenylated with 5 μM of thermostable *Methanobacterium thermoautotrophicum* RNA ligase (NEB) in the presence of 100 μM rATP at 55°C for 1 hr. Then, incubation at 80°C for 5 min inactivates the pre-adenylation reaction.^159^ Finally, the 20 μl reaction was topped up to 50 μl with distilled water and the adenylated adaptor purified using Oligo Clean and Concentrator kit (Zymo Research) as per manufacture’s recommendation and was eluted using 6 μl of distilled water. In our hands, the recovery is ~90% with a final concentration of pre-adenylated (App-CBD610) adaptor of 18 μM.

#### Library preparation from fidelity experiments

To characterize and quantify the extent of misincorporation, we isolated close to full-length RNAs from both −coupling and +coupling reactions and prepared pooled libraries for high-throughput sequencing (Figure 4E). Each library was constructed to contain a pair of *i5* and *i7* index sequences for downstream demultiplexing. Within each library, we implemented Unique Molecular Identifier (UMI) barcoding of individual RNA molecules, which provides a handle to group, align, and retrieve consensus sequences from reads carrying the same UMI^158^ (Figure 4E; see STAR Methods). In the first step of library preparation from the fidelity experiment (bubble assembly using RNA CBR27/384 with DNA template strand CBD451 and non-template strand CBD450), we ligated RNAs to pre-adenylated DNA adaptor (App-CBD610), which bears complementary sequence to a primer for reverse transcription (RT). In a 10 μl reaction, 2 ng/μl (~0.8 pmol) of RNA in the size range between 80–90 nt was incubated with 10 pmol of App-CBD610, 10 U/μl of truncated T4 RNA ligase 2, K227Q (NEB), 2 U/μl RNaseOUT^™^ (Invitrogen), 17.5% PEG8000 (w/v) in 1X T4 RNA ligase buffer at 22°C for 3 hr. Excess App-CBD610 were digested using a combination of 2.3 U/μl of yeast 5′ deadenylase (NEB) and 1.4 U/μl of RecJ_f_ (NEB) at 37°C for 1 hr followed by the inactivation of the enzymes at 70°C for 20 min. Yeast deadenylase removes the 5′ App moiety while RecJf degrades the unprotected monophosphorylated adaptors. We achieved complete removal of the excess adaptor while leaving the ligated product intact. Next, ligated product was purified using Oligo Clean and Concentrator kit (Zymo Research) and eluted with 6 μl of distilled water. Using a similar sized control RNA, we found that the ligation efficiency was ~40% and that the recovery from column purification was ~90% giving rise to an estimated purified ligated product of ~1.2 ng/μl. RT was carried out using 1 ng of ligated product with 10 U/μl Superscript^™^ IV (Invitrogen), 2 U/μl RNaseOUT^™^ (Invitrogen), 0.5 mM dNTPs, 5 mM DTT, 1X Superscript IV buffer in a final volume of 20 μl at 55°C for 10 min. Then, the RT reaction was inactivated at 80°C for 10 min. Likewise, second strand synthesis on cDNAs was carried out as described above using excess UMI assigning primer except that the annealing of the UMI assigning primer to the cDNA was lowered to 55°C. Similarly, following exonuclease I treatment to remove the RT and UMI assigning primers, the starting duplex was amplified using forward and reverse primer for 27 cycles. The amplified library was purified twice using Select-a-Size DNA Clean & Concentrator^™^ MagBead Kit (Zymo Research) but with 50 μl of MagBead given that the expected library size is smaller than the earlier library (~211 bp). We obtained 2 libraries that correspond to reactions without (−coupling) and with (+coupling) concomitant translation. Finally, the concentrations and purities were determined by qRT-PCR and by fragment analysis respectively prior to Mi-Seq sequencing.

#### Data analysis

We have archived the raw dataset (doi:10.17632/ysc6r3dz2m.1) and the analysis pipeline (doi:10.5281/zenodo.6534021). First, we used deML,^160^ an algorithm that uses maximum likelihood principle to demultiplex raw sequencing reads into their respective libraries (−coupling and +coupling reactions from fidelity experiments and transcription of *pyrL*), each with its pair of unique *i5* and *i7* indexes. Unlike a paired-end library where each read is composed of 2 mates, our single-end library has only a single mate, which for convenience, I will henceforth refer to as a read. Each read begins with the 14 nt UMI tag and is followed by the sequence of interest. The average read length for all our libraries is 151 nt. Approximately 88.2% of our total reads were confidently assigned to a particular library and were subjected to further downstream analysis. The remaining 11.8% of problematic reads (classified as unknown, conflict and wrong reads) that cannot be unambiguously assigned were excluded from the analysis. Sequences of the library indices (*i5* and *i7*) and primers that introduce these indices are provided in Table S4. Demultiplexed libraries are individually pre-processed using HTStream to screen out PhiX reads, to remove adaptor sequences and to discard sequences below an average Phred quality score of 20 using a 10 nt moving window. UMI sequences located at the 5′ end of the reads were not trimmed. Next, we employed Calib, which is an alignment-free algorithm that accounts for sequencing errors to cluster reads by their UMI.^161^ Calib, however, takes in paired-end reads as input. To satisfy this requirement, we generate the corresponding mate pair from our single-end reads for each library by removing the beginning 14 nt UMI sequences using the “UMIextract.awk” script (doi:10.5281/zenodo.6534021). Our mate-pairs for each library are then fed into Calib for clustering. Calib allocates identical reads (very closely matched sequence of interest and UMI tag) into a common node. Reads that differ slightly in sequences but are below the error threshold are considered connected nodes that belongs to the same cluster. Ultimately, reads that are within the same clusters are assumed to originate from a common ancestor molecule. For our UMI of 14 nt, they are deemed the same if they fall within *e* = 2 Hamming distance. As a measure of similarities among sequences of reads, Calib performs MinHashing with the following default settings: *k*-mer of size 8 extracted from each of the *m* = 7 non-overlapping segments (minimizers) of each read and a minimizer error threshold of *t* = 2.^161,162^ The consensus sequence for each cluster is obtained by column-wise majority voting of multiple sequence alignment built using single instruction multiple data (SIMD) implementation of the partial order alignment (POA) algorithm.^163,164^ Finally, all consensus reads for each library is aligned against a reference sequence to obtain the counts of bases occurring at each position of the reference sequence using Mafft.^165^ When running Mafft, we included the 6merpair argument to speed up the alignment process, kept the numbering of sites in accordance to the reference sequence and removed reads that contained more than 5% of ambiguous letters.

#### Single particle Cryo-EM

##### Preparation of RNA for TTC assembly

The TTC for cryo-EM was assembled on an 81 nt RNA (CBR27/479–21) that forms an rU-dG terminal mismatch. We introduced three consecutive phosphorothioate bonds at the 3′ end of the RNA to inhibit endonucleolytic cleavage of the terminal mismatch by RNAP during assembly in the presence of divalent magnesium. To obtain the RNA, we performed splint ligation of a longer 62 nt RNA (CBR27/479) to a shorter 19 nt RNA (CBR21) using a DNA splint (CBD582). We produced CBR27/479 by *in vitro* transcription on annealed synthetic DNA templates between CBD27 and CBD479. CBR21 carrying a 5′ phosphate and phosphorothioate modifications was purchased from IDT. In the first annealing step, 60 pmole of each RNA fragment was added to 42 pmole of DNA splint in 1X ligation buffer (50 mM Tris-HCl, 10 mM MgCl_2_, 10 mM DTT, pH 7.5), heated to 80°C for 2 min, cooled to 25°C and incubated for 5 min in 15 μl volume. Consequently, the two RNA fragments were bridged by the 24 nt DNA splint—12 base pair complementarities to each RNA fragment—for ligation. Ligation was initiated with 4000 units of T4 DNA ligase (NEB), 1 mM (f.c) ATP in a final volume of 20 μl at 30°C for 5 hr.^166^ Then, after a single round of phenol/chloroform extraction, we purified the ligated RNA with 8% denaturing urea gel.

#### TTC assembly

We obtained the TTC by first assembling the transcription elongation complex. Through the same sequential bubble assembly, 42 pmol of core RNAP was added to 48 pmol of RNA (CBR27/479–21)-DNA template (CBD573) hybrid in 1X Cryo-EM buffer (CB: 20 mM HEPES, 95 mM KCl, 50 mM NH_4_Cl, 5 mM MgCl_2_ and 1 mM DTT, pH 7.5) with 50 μl Dynabeads^®^ M-280 streptavidin magnetic beads (Thermo Fisher Scientific) and incubated at 37°C for 10 min in an initial assembly volume of 172 μl. To complete the elongation complex, 21 pmole of non-template strand (CBD572) was added and incubated at 37°C for an additional 10 min in a final assembly volume of 200 μl. The elongation complex was immobilized on magnetic beads via a desthiobiotin tag at the 5′ end of the DNA template strand. The transcription elongation complex was washed with 3X, each with 200 μl 1X CB buffer to remove unincorporated components. Then, we assembled the ribosome on RNA attached to the transcription elongation complex with 400 pmol of 70S ribosome, 400 pmol of fMet-tRNA^fMet^ (tRNAprobe), 400 pmol each of initiation factor 1, 2 and 3 and 4 nmol of rGTP and incubated at 37°C for 15 min in 200 μl. The assembled TTC was then washed 3X each with 200 μl 1X CB buffer before translocating the ribosome towards RNAP. In a 200 μl translocation reaction that was allowed to proceed at 37°C for 15 min, we included 65 μM of uncharged total tRNA from MRE600 (Roche), 2 mM rATP, 2 mM rGTP, 120 μM total amino acid and 1X Factor Mix in 1X CB buffer with 5 mM free Mg^2+^ concentration (Tables S1B–S1F). Following the reaction, we washed the sample once with 200 μl 1X CB buffer, transferred the sample to a new tube and conducted two more washes each with 100 μl 1X CB buffer. Finally, we harvested the TTC from the magnetic beads in two steps. In the main elution step, 15 μl of elution buffer (5 mM D-biotin (Invitrogen), 3% trehalose (w/v), in 1X CB buffer) was used to elute TTC from the magnetic beads at 37°C for 15 min. In the second step, we rinsed the beads with 5 μl of elution buffer, which was then pooled with the first eluate to yield 20 μl of TTC.

#### Sample deposition and data collection

Cryo-EM specimens were prepared on carbon-coated C-flat-1.2/1.3 400 mesh copper grids (Protochips) that were glow-discharged using a Tergeo-EM plasma cleaner (PIE Scientific). Onto these grids, 3 μl of the sample were deposited, blotted for 6 sec with a blot force of 6 at 22°C in 100% humidity and vitrified by plunging into liquid ethane using a Vitrobot Mark IV (Thermo Fisher Scientific).

Electron micrographs were acquired as dose-fractionated movies with a 200 keV Talos Arctica cryo-electron microscope (Thermo Fisher Scientific) using a K3 direct electron detector (Gatan) operated in super-resolution counting mode. The microscope was set to 28,000× magnification (super-resolution pixel size of 0.7235 Å/pixel) with a total exposure dose of 50 electrons per Å^2^ fractionated across 50 frames. A total of 5,761 movies were recorded with defocus values ranging from approximately –1 μm to –2.5 μm and data collection was automatically controlled using SerialEM.^167^ Data collection parameters are summarized in Table S3.

#### Data Processing

The processing of the cryo-EM data was performed using RELION 3.1^168,169^ and cryoSPARC v3.1.0^170,171^ as detailed in Data S1. Movie frames were aligned using MotionCor2^172^ within RELION and binned 2× (to 1.447 Å/pixel). Defocus estimation and contrast transfer function (CTF) fitting were performed using the Gctf package^173^ in RELION. In the corrected micrographs, we can readily observe particles that correspond either to the free RNAP harboring the single mismatch (${\text{RNAP}}_{\text{Free}}$) or to the RNAP associated with the ribosome in the TTC (see Data S1). Data processing was conducted separately for the ${\text{RNAP}}_{\text{Free}}$ and for TTC. A preliminary round of data processing (data not shown) was performed with part of the dataset whereby particles were picked using the Laplacian-of-Gaussian (LoG) algorithm in RELION 3.1. For the ${\text{RNAP}}_{\text{Free}}$ multiple rounds of 2D-classification/particle-selection were performed to get 2D classes that served as templates for auto-picking. In the case of the TTC, after several rounds of 2D/3D classification, an initial 3D reconstruction was obtained serving as the 3D template for auto-picking as outlined in the processing workflow in Data S1.

For ${\text{RNAP}}_{\text{Free}}$, initially 1,941,400 particles were picked and extracted 2× binned at 2.894 Å/pixel in 120-pixel boxes for 2D classification. This step was followed by an initial 3D reconstruction, and several rounds of 3D classification and 3D refinement. 224,236 of those particles were extracted with 240-pixel boxes at 1.447 Å/pixel, then exported to cryoSPARC for a new 2D classification round leading to selecting 216,710 particles used to carry out *ab-initio* 3D reconstruction (N=3). Two of the three initial reconstructions, constituting 169,685 particles, were selected, and subjected to heterogeneous refinement. We then performed a non-uniform refinement with 118,450 particles resulting in a cryo-EM density map at 4.4 Å overall resolution (FSC = 0.143). These particles were exported back to RELION to perform CTF refinement and particle polishing, followed by 3D auto-refinement, resulting in a final cryo-EM reconstruction of the ${\text{RNAP}}_{\text{Free}}$ at 3.9 Å overall resolution (FSC = 0.143) (see Data S1).

For the TTC, as mentioned before, particles were picked using the 3D template-based autopicking in RELION 3.1 (Data S1). Initially, 1,250,640 particles were picked and extracted 2× binned at 2.894 Å/pixel in 220-pixel boxes and then subjected to three consecutive rounds of 2D classification, resulting in 103,961 selected particles. After two rounds of 3D classification, 52,615 particles from two 3D classes were selected and extracted in 440-pixel boxes at 1.447 Å/pixel, to yield a 3D reconstruction that refined to 5.1 Å overall resolution (Data S1). In this reconstruction, the density map of the 70S ribosome and RNAP were immediately discernible. Nonetheless, the density corresponding to the RNAP appeared fuzzy (Data S1), indicating some degree of heterogeneity. A first round of multi-body refinement confirmed that RNAP has a continuous orientational heterogeneity relative to the ribosome (see Data S1). According to the uni-modal distributions for the amplitudes (eigenvalues) of the particles along the first three principal components (eigenvectors), we observed that particles on the extreme eigenvalues generated TTC structures with inter-molecular clashes (Movie S1). To overcome this problem, a subset of 31,191 particles with eigenvalues from −10 to 10 (a range close to the central region of the distribution) along the first principal component, were selected as indicated in Data S1. These particles were then subjected to a new 3D refinement step, followed by a second round of multi-body refinement, but some degree of heterogeneity still remained. Therefore, after visual inspection of the movements along the first three principal components, a total of 18,629 particles, with a narrower range of eigenvalues, from −6 to 6 along the third principal component (see Data S1), were selected and subjected to a new 3D refinement and multi-body refinement round (Data S1). After this last step, little heterogeneity appeared to remain (Movie S1). Then, in order to improve the resolution in both, the RNAP (${\text{RNAP}}_{\text{TTC}}$) and 70S ribosome regions of the TTC, signal subtraction and focused 3D refinement were performed separately to generate independent 3D reconstructions for the 70S ribosome and ${\text{RNAP}}_{\text{TTC}}$ regions, yielding reconstructions at 3.8 Å (FSC = 0.143) and 7.3 Å (FSC = 0.143) nominal resolutions, respectively (see Data S1).

All of the cryo-EM maps obtained were then sharpened using the post-processing program in RELION 3.1, applying global B-factors as detailed in Table S3, and atomic coordinates were refined against them as detailed in the next section.

To investigate the ${\text{β}}^{\prime }\text{SI3}$ mobility in both the ${\text{RNAP}}_{\text{Free}}$ and ${\text{RNAP}}_{\text{TTC}}$ conformations, we performed a multibody analysis in both structures by defining one body as the density region corresponding to the ${\text{β}}^{\prime }\text{SI3}$ domain, and the other body as the remaining density region of the RNAP, named as RNAP ‘core’ body (Figure S6B). The multibody analysis showed that along principal directions, the ${\text{β}}^{\prime }\text{SI3}$ domain displays limited mobility in the ${\text{RNAP}}_{\text{Free}}$, while exhibits a large range of motions in the ${\text{RNAP}}_{\text{TTC}}$ (Figures S6C, S6D and Movie S2).

#### Model Building and Refinement

For the ${\text{RNAP}}_{\text{Free}}$ and ${\text{RNAP}}_{\text{TTC}}$ structures, initial models were obtained by rigid-body fitting the atomic coordinates of the backtracked EC (PDB 6RI9) into the corresponding post-processed maps,^42^ using UCSF Chimera.^174^ For each complex, the models were then iteratively rebuilt in COOT^175^ and refined using the real space refinement program in PHENIX.^176^ For the ${\text{RNAP}}_{\text{Free}}$, its final refined model was then used to perform the local sharpening of its corresponding 3D map using LocScale.^177^

The initial model of the 70S ribosome in the TTC was obtained by placing the crystal structure of the empty ribosome (PDB 5IT8)^178^ into the cryo-EM map using the rigid-body fitting tool in Chimera and the *dock in map* program in PHENIX. The coordinates of the L31 subunit, initially missing in the starting crystal template, were modeled using the SWISS-MODEL online server^179^ and rigid-body fitted using Chimera. Coordinates for the mRNA and t-RNA were taken from the collided expressome complex (PDB 6ZTL),^69^ and rebuilt in COOT. The full model of the 70S ribosome was iteratively refined with the real space refinement program in PHENIX. The final refined model of the Ribosome 70S region was used to perform the local sharpening of its corresponding 3D map using LocScale. Finally, the *combine_focused_maps* program in Phenix^176^ was used to get the full TTC density map as well as its full coordinate model. All validation and refinement statistics are shown in Table S3.

#### Models Comparison

The ${\text{RNAP}}_{\text{Free}}$ (PDB 8FIX) and ${\text{RNAP}}_{\text{TTC}}$ (PDB 8FIY) final coordinates were aligned by superimposing their RNAP core module regions (residues 1071–1235), using the *superpose* program in ccp4.^180^ The resulting transformation matricies were then used to align the corresponding ${\text{RNAP}}_{\text{Free}}$ (EMD-29212) to the ${\text{RNAP}}_{\text{TTC}}$ (EMD-29213) cryo-EM maps using the *coord_transform_to_star* program (https://github.com/dominikaherbst/cryo-em_scripts), and used the *volume resample* tool in ChimeraX to generate the aligned version of the ${\text{RNAP}}_{\text{Free}}$ cryo-EM map. Finally, all the rotations and root mean square (RMS) deviations measurements were performed in PyMOL v1.6 (Shrodinger, 2015, https://pymol.org/2/) and are reported in Table S3.

#### Cell growth assay

The bacterial strains used in the cell growth assay are described in the Key Resources Table. Cells were grown overnight at 37 °C in Luria Bertani (LB) broth. The overnight cultures were diluted 200-fold in fresh LB and were cultured at 37°C to log phase (${\text{OD}}_{600}$ ~ 0.4–0.5). Cells were diluted to prepare two-fold serial dilutions starting from ${\text{OD}}_{600}$ = 6.25 × 10^−4^. Subsequently, 1.5 μl of the diluted cultures were spotted on three separate Mueller Hinton Agar plates containing 5% DMSO and: 1) no additional additive (control), 2) omeprazole (250 μg/ml), 3) omeprazole (250 μg/ml) and pseudouridimycin (250 μg/ml) or 4) omeprazole (250 μg/ml) and fidaxomicin (75 μg/ml). Plates were then grown at room temperature for 3 days.

#### Stalled RNAPs cause ribosome collisions

A ribosome can stall on damaged or suboptimal mRNA, which can lead to a built-up of collided ribosomes.^181–184^ To pre-empt ribosome collisions, the stalled ribosome is targeted for recycling (ribosomal rescue), in which incompletely synthesized proteins and presumably faulty mRNA are destroyed.^185^ One notable conserved ribosomal rescue mechanism is the SsrA/tmRNA system that frees ribosomes that are stalled on the 3′ end of mRNAs and marks the nascent peptide with a degron tag for proteolysis.^186^ Another involved the SmrB protein that recognizes the unique interface between collided ribosomes and cleaves the underlying mRNA. The trailing ribosome that is freed can translate to the end of the cleaved mRNA and be recycled by the SsrA/tmRNA system.^116^

Can a stalled RNAP cause a translational ‘traffic jam’ that will activate the ribosomal rescue pathway? We have seen that a coupled ribosome can activate a paused RNAP; we wondered what would be the effects on the cell if the ribosome is unable to rescue a stationary RNAP. To do this, we performed a series of cell viability assays in which we stalled RNAP with pseudouridimycin (PUM)^117^ in cells that have deletions in genes important for ribosomal rescue (*ssrA*, *smrB*, and the *smrB* paralog *smrA*). We also treated the cells with omeprazole, an efflux pump inhibitor that prevents PUM from being expelled.^187^ If stalled RNAP do cause ribosomes to collide, we expect a reduced viability in cells with ribosomal rescue genes knocked out.

In DMSO- and omeprazole-treated controls, WT and mutant strains exhibited no difference in growth rate, suggesting that under non-stress conditions ribosomal rescue pathways are not significantly invoked (Figure S7A). We stressed the cells by inhibiting transcription elongation with PUM and observed changes in the cell viability for the knockout strains. The ΔssrA strain showed severe growth defects compared to the WT strain, suggesting that *ssrA* is a key player for ribosomal rescue induced by a stalled RNAP. By contrast, ΔsmrA and ΔsmrB strains grew better than the WT and ΔssrA strains. We attribute this observation to the fact that less mRNA is cleaved and degraded in ΔsmrA and ΔsmrB cells that have fewer mRNAs to begin with due to the PUM treatment. Likewise, in the ΔssrAΔsmrB double mutant, we also observed an increase in the fitness when compared to ΔssrA alone. We hypothesize that prolonged stalling of the RNAP by PUM can cause ribosome-ribosome collisions that leads to a ribosomal “traffic jam”. To resolve these collisions, incomplete mRNA and nascent proteins must be degraded, which is energetically costly for the cells already under stressful conditions.

By comparison, inhibiting transcription initiation with fidaxomicin allows us to control for the effect of the reduction in RNA synthesis and eliminate ribosomes collisions caused by a stalled RNAP. Unlike the trends seen in the PUM-treated cells, we see only minor effects on cell viability in the context of ribosomal rescue genes knockouts compared to wild-type with fidaxomicin (Figure S7A). Taken together, we see that long pauses of an RNAP can be very costly to the cell because of the mechanism by which ribosomal collisions are resolved. We hypothesize that coupling can confer an fitness advantage by preventing ribosome-ribosome collisions by reducing the duration of pausing in RNAPs, even at the expense of fidelity (Figure S7B).

#### Derivation and Kinetics

##### Extracting pause sites kinetics

We quantified the pause bands (P) for every lane—corresponding to various time points—in the denaturing urea gel with ImageQuant TL 8.2. Each band’s intensity was normalized to the total (T) band intensity for that lane (time point) to correct for variations in sample loading. The decay rate for the normalized pause band (P/T) gives the pause half-life when fitted either to a single exponential (for HP site) or a sum of two exponential (for P1) function. In a log(P/T) versus time plot, a single line fit describes the decay of HP pause while a piecewise regression with 1 breakpoint (2 linear segments) characterizes the two decay rates of P1 pause.

To obtain the apparent pause efficiency of HP pause, we extrapolated the linear fit to the y-axis at *t* = 0. For P1 pause, extrapolating the 2 linear segments give the apparent pause efficiencies (*E*) for the slow ($E_{\text{slow}}$) and the sluggish ($E_{\text{sluggish}}$) population of RNAP. To obtain the actual pause efficiencies at P1 and the HP site, we first calculated the sum of band intensity from the pause band and farther (A) normalized to the total (T) band intensity for every timepoint. Fitting this cumulative plot of A/T against time yielded the actual percent of RNAP (F) that arrived at the pause sites observed in the experiment. Through a second normalization of the normalized pause band intensity (P/T) by the percent of RNAP (F) that made it to the pause site, we acquired an estimate of the inherent pause efficiency (*E*) if 100% of RNAP were to arrive at the pause site. After accounting for the percent of RNAP that paused, the remaining (add up to 100%) corresponded to RNAP that bypass pausing.

We apply the methods of Landick and colleagues to extract the associated kinetic parameters.^188^ If a pause duration follows a pseudo-first order kinetic, the rate of change in the proportion of paused RNAP (P) is given by

$$\frac{d\text{P}}{dt}=-k_{-\text{p}}{\text{P}}_{0}$$

where $k_{-\text{p}}$ is the intrinsic pause escape rate and ${\text{P}}_{0}$ is initial proportion of paused RNAP. By integration, the proportion of paused RNAP at any given time P(t) can be expressed as

$$\text{P}(t)={\text{P}}_{0}{\text{e}}^{-k_{-\text{p}}t}$$

Accordingly, the average pause duration (T) is given by the inverse of $k_{-\text{p}}$,

$$T=\frac{1}{k_{-\text{p}}}$$

and the pause half-life $\left(t_{1/2}\right)$, defined by the time it takes for the proportion of paused RNAP to decrease by half will be

$$t_{1/2}=\frac{\ln(2)}{k_{-\text{p}}}$$

We plotted logP(t) against time t, which if it follows a single exponential decay would be a straight line, which is apparent from the following derivation.


$$\text{logP}(t)=\log{\text{P}}_{0}{\text{e}}^{-k_{-\text{p}}t}$$

$$\log\text{P}(t)=\log e^{-k_{-\text{p}}t}+\log{\text{P}}_{0}$$

$$\log\text{P}(t)=\frac{\ln e^{-k_{-\text{p}}t}}{\ln(10)}+\log{\text{P}}_{0}$$

$$\log\text{P}(t)=\frac{-k_{-\text{p}}}{\ln(10)}t+\log{\text{P}}_{0}$$

From the plot, the slope (*s*) of the line is given by

$$s=\frac{-k_{-\text{p}}}{\ln(10)}$$

and the y-intercept is given by

$$\text{y-intercept}=\log{\text{P}}_{0}$$

Therefore, we can determine $k_{-\text{p}}$ from the slope of the line

$$k_{-\text{p}}=-\sin(10)$$

And we can calculate ${\text{P}}_{0}$ from the y-intercept obtained through back extrapolating the line to time zero. Here ${\text{P}}_{0}$ is equivalent to the pause efficiency E.


$$\log({\text{P}}_{0})=\text{log}(E)=\text{y-intercept}$$

$$E=10^{\text{y-intercept}}$$

Finally, the final reported E was obtained by normalizing to the total population of RNAP that made it to the pause site at infinite time to account for those that did not within the experimental time frame.

#### Association of pause efficiency and apparent pause duration in the pause re-entry scenario

The relationship between pause efficiency (E) and the apparent pause duration ($T^{\text{app}}$) had been worked out by Herbert and colleague.^29^ Briefly, at any given pause site, the competition between the rate of entry into an off-pathway pause ($k_{\text{p}}$) and the rate of on-pathway elongation ($k_{\text{n}}$) governs E. On the assumption that most RNAP ended up as a slow elongation complex and the conversion to a slower elongation complex is rare (Figure 2A), E can be simplified to

$$E=\frac{k_{\text{p}}}{k_{\text{p}}+k_{\text{n}}}$$

If E is considerable at a strong pause site, RNAP that escape pausing will be more likely to re-enter the pause state. The re-entry into the pause state results in $T^{\text{app}}$ that is longer than the intrinsic pause duration (T) which is implicit in our gel-based assay that measures only the resumption of transcription activity. The effective rate for a failed pause escape (RNAP revisits the pause state) is therefore given by the product of $k_{-\text{p}}$ and E. By contrast, the effective rate for a successful pause escape (RNAP does not revisit the pause state) is given by the product of $k_{-\text{p}}$ and (1−E). Hence, we have

$$T^{\text{app}}=\frac{1}{k_{-\text{p}}(1-E)}$$

$$=\frac{T}{\left(1-E\right)}$$

$$=\frac{k_{\text{p}}+k_{\text{n}}}{k_{\text{n}}k_{-\text{p}}}$$

### QUANTIFICATION AND STATISTICAL ANALYSIS

All statistics were determined in Excel. Quantification details are indicated in the figure legends.

## Supplementary Material

1 Data S1. Cryo-EM data processing, related to Figure 6.

2 Figure S1. Characterization of Pause 1 (P1) through border mapping by ExoIII digestion, related to Figure 1 and STAR Methods
(A) Schematics of upstream border mapping (top panels) and downstream border mapping (bottom panels) of RNAP. Each panel indicates border mappings for the reference polymerase (RNAP-U9) and after the enzyme has translocated by one base pair (RNAP-A10). The outlines in blue, green and purple denote the various footprints for RNAP in the pre-, post-, and hyper-translocated states respectively. Lines at the queried border denote the expected labeled DNA fragment lengths after ExoIII digestion, which correspond to the three states.
(B) Diagram showing the RNA-DNA hybrid base pairing of a pre-translocated RNAP at P1. The G_−11_G_−10_Y_−1_G_+1_ elemental pause motif is shown above the sequences.
(C) Experimental workflow of the ExoIII border mapping experiment. Step-wise bubble assembly of an elongating RNAP gives the reference enzyme (RNAP-U9), which can be sequentially stepped into RNAP-A10 and RNAP-U11. Then, ExoIII is added to query either the upstream or the downstream borders over time.
(D) DNA template borders as mapped by ExoIII digestion at U9, A10, and U11 (first three panels). Right panel, nonhydrolyzable rUTP analog UpNHpp fails to return a hyper-translocated RNAP-A10 to a post-translocated A10.
(E) The hyper-translocated RNAP-A10 is less prone to pyrophosphorolysis than the post-translocated RNAP-U9, which shows a higher rate of RNA shortening in the presence of potassium pyrophosphate (KPPi).

3 Figure S2. Active translation speeds up coupled transcription, related to Figure 2
(A) RelE binds a ribosome with an empty A-site and cleaves the underlying RNA between the second and the third nucleotide of the codon (left panel). The loading efficiency of the ribosome on the RNA, determined by RelE cleavage, is ~72.0 ± 2.6% (right panel). The lane for −ribosome control and the lanes for +ribosome incubated with increasing concentrations of rGTP were spliced together from the same piece of gel. The splicing interface is denoted by the dotted line.
(B) The application of a high (15 pN) force by optical tweezers unfolds RNA hairpins and *vice versa* (left panel). RNAP avoids transcription termination when the terminator hairpin unwinds (right panel). Representative traces are shown in different colors.
(C) A rapid force drop from 15 pN to 5 pN allows the terminator hairpin to refold, which will terminate transcription.
(D–K) Translation elongation inhibitors such as tetracycline (blue) and fusidic acid (green) suppressed the effects of the ribosome on the pause efficiencies, overall velocity, termination and runoff probabilities of RNAP during coupling. Data are mean ± SD for four independent experiments.

4 Figure S3. Coupling reduces transcription pause density and duration, related to Figure 3
(A) Fluorescent lifetime of fMet-tRNA^fMet^-JF549 in the ribosome is an order longer in a stationary ribosome than of a translocating ribosome. This indicates that the measurement of its lifetime (dwell time of fMet-tRNA^fMet^-JF549 in the ribosome before dissociating) in translocating ribosome is not limited by its photobleaching time.
(B) Transcription pause densities (number of pauses > 0.5 s per bp) in opposing and in assisting force setup under −coupling (grey) and +coupling (black) conditions.
(C) Crossing times for RNAP at P1 are shown for each condition, along with a biexponential fit. The fit parameters are in Table S2C.
(D) Plots of the pause durations in each condition taken from the fits in (C).

5 Figure S4. RNAP tends to misincorporate when coupled to the ribosome, related to Figure 4
(A) The percentage of RNAs that contain 0, 1, 2, 3 and >3 mismatches (MM) for −coupling and +coupling reactions (left and right pie charts) for the fidelity assay (top pie charts) and for transcription on *pyrL* with limiting rUTP (bottom pie charts).
(B) Along *pyrL* and regardless of coupling, RNAP tends to misincorporate at uridine sites that are immediately preceded by cytidine sites. At these U sites, cytidine is preferentially incorporated.
(C) RNAP shows a higher misincorporation rate along *pyrL* when coupled to the ribosome given that more sites occupy the right region of the volcano plot that corresponds to positive relative percent error when comparing between the +coupling and the −coupling reactions. Of the 114 positions surveyed, 20 contained more mistakes in the +coupling reaction than in the −coupling reaction, which are statistically significant (Fisher exact test, *p-value* < 0.05). These 20 positions are highlighted in red in the volcano plot. At these positions, the misincorporated ribonucleotides are mostly uridine or cytidine.

6 Figure S5. The ribosome prevents error rectification by RNAP during coupling, related to Figure 5
(A) A schematic depicting the bubble construct to test dinucleotide cleavage (error rectification) by RNAP after misincorporation of rGTP at the −1 position.
(B) The experimental workflow to measure error rectification by RNAP.
(C) Reaction at 37°C drives more rGTP misincorporation (12G) by RNAP than at 25°C.
(D) Time-dependent intrinsic hydrolysis of RNA by RNAP bearing a terminal mismatch (*p-mt; simulating ${\text{ES}}_{56}$) using the bubble construct shown in Figure 4A. The transcription elongation complex (RNAP is 10-fold in excess of RNA and DNA templates) was assembled in the absence of Mg^2+^, and the hydrolysis of *p-mt was monitored when 4 mM Mg^2+^ was added. Di- and tri-nucleotide cleavage from the terminal end of *p-mt predominates, resulting in 79 nt and 78 nt RNA species respectively.
(E) Quantification of (D) showing the percentage of *p-mt hydrolyzed with time in the presence of 4 mM Mg^2+^. A ligation time of 10 min for attaching DNA handles to RNAP was selected, which correspond to ~21% of RNAP with its RNA cleaved.
(F) RNAP carrying RNA with a terminal rG-dA mismatch was incubated with saturating rNTPs (500 μM each) for 3 min to determine the percent active enzyme (the percentage of RNAP that had extended its RNA beyond 81 nt), which was increased in the presence of Gre factors. There is limited hydrolysis of RNA carrying the terminal mismatch after 10 min of incubation in 4 mM Mg^2+^ (lane 3). Lane 4 shows RNAP that was subjected to an additional 3 min incubation with 500 μM rNTPs after 10 min of preincubation (10>3) in 4 mM Mg^2+^ that was increased to 10 mM (4>10) at the same time when rNTPs were introduced. The cleavage of *p-mt to remove the offending ribonucleotide through intrinsic hydrolysis by RNAP is inefficient (lane 3) and can be improved with the assistance of GreA and GreB (lanes 5 and 7). GreA cleaves *p-mt to produce mainly dinucleotide fragments (lane 5) whereas GreB yields equivalent amount of di- and tri-nucleotide products (lane 7). All incubations were performed at 25°C.
(G) The percent of active RNAP during 3 min incubation in 10 mM Mg^2+^ with 500 μM rNTPs as shown in (F) was similar to RNAP that was preincubated for 10 min in 4 mM Mg^2+^ (less than 4.5% difference in active polymerase). In the presence of Gre factors, the percentage of active RNAP increased by as much as 13%.
(H) In line with a higher percent of active RNAP in the presence of Gre factors than in their absence as quantified in (G), there was a corresponding increase in the cleavage of *p-mt to remove the terminal rG-dA mismatch that reset the polymerase to its active state. Yet, a considerable percentage of RNAP remained stalled at the start site (81 nt band) after intrinsic hydrolysis (41%), GreA (33%) or GreB (31%) cleavage.

7 Figure S6. Extent of swiveling found in ${\text{RNAP}}_{\text{Free}}$ and ${\text{RNAP}}_{\text{TTC}}$, and focused ${\text{β}}^{\prime }\text{SI3}$ multibody analysis, related to Figure 6
(A) Left: ${\text{RNAP}}_{\text{Free}}$ versus ${\text{RNAP}}_{\text{6ALH}}$ models. Coordinates of both structures were aligned relative to the core module of RNAP (gray ribbon) and it was observed that the swivel module of ${\text{RNAP}}_{\text{Free}}$ (‘swiveled’, hot pink) was rotated +1.5° towards a swiveled direction relative to its location in the ${\text{RNAP}}_{\text{6ALH}}$ (‘non-swiveled’, blue). Right: ${\text{RNAP}}_{\text{TTC}}$ versus ${\text{RNAP}}_{\text{6ALH}}$ models. Coordinates of both structures were aligned relative to the core module (gray ribbon), and it was observed that the swivel module of ${\text{RNAP}}_{\text{TTC}}$ (‘anti-swiveled’, green) was rotated −1.7° towards an anti-swiveled direction relative to its location in the ${\text{RNAP}}_{\text{6ALH}}$ (‘non-swiveled’, blue).
(B) Analysis of ${\text{β}}^{\prime }\text{SI3}$ dynamics by multibody refinement in the ${\text{RNAP}}_{\text{Free}}$ (left) and ${\text{RNAP}}_{\text{TTC}}$ (right). The ${\text{β}}^{\prime }\text{SI3}$ domain shows a limited movement in the ${\text{RNAP}}_{\text{Free}}$, whereas it displays a large dynamic in the ${\text{RNAP}}_{\text{TTC}}$. In the ${\text{RNAP}}_{\text{Free}}$, the ${\text{β}}^{\prime }\text{SI3}$ domain has a limited mobility, with a rotation amplitude of up to ~7°. In the ${\text{RNAP}}_{\text{TTC}}$, the ${\text{β}}^{\prime }\text{SI3}$ domain displays a large flexibility with up to ~45° of inward rotation, showing alternate between the ‘in’ (orange) and ‘out’ (green) conformations.
(C) Eigen-values distribution along the first two principal components of the Multibody analysis for ${\text{RNAP}}_{\text{Free}}$ and ${\text{RNAP}}_{\text{TTC}}$.

8 Figure S7. RNAP stalling causes ribosome-ribosome collisions, related to STAR Methods
(A) Sections of agar plates showing growth of two-fold serial dilutions of *E.coli* strains (left label), with or without transcription elongation inhibitor pseudouridimycin (250 μg/ml) or transcription initiation inhibitor fidaxomicin (75 μg/ml). To improve the efficiency of antibiotic incorporation, Mueller Hinton agar was used and the cells were also treated with omeprazole (250 μg/ml).
(B) Model for RNAP stalling causing ribosome-ribosome collisions and their prevention by a coupled ribosome.

9 Movie S1: Related to Figure 6 and Data S1. This movie shows the three rounds of multibody refinement performed on the TTC.

10 Movie S2: Related to Figures 6 and S6. This movie shows the results after multibody refinement performed to analyze the ${\text{β}}^{\prime }\text{SI3}$ dynamics in both the ${\text{RNAP}}_{\text{Free}}$ and ${\text{RNAP}}_{\text{TTC}}$.

11 Table S1: Reaction mix for the −coupling and the +coupling reactions in *pyrL* transcription, fidelity assay and optical tweezers experiments, related to STAR Methods.

12 Table S2: Transcription kinetic parameters from bulk and single molecule optical tweezers experiments, related to Figures 2 and 3.

13 Table S3: Cryo-EM data collection, refinement, and validation statistics, related to Figure 6 and Data S1.

14 Table S4: Oligonucleotides, related to STAR Methods and Key Resources Table.

## Figures and Tables

**Table:** Key resources table

REAGENT or RESOURCE	SOURCE	IDENTIFIER
Antibodies		
Purified anti-*E. coli* RNA polymerase α antibody [4RA2], monoclonal	BioLegend	Cat#663102; RRID:AB_2564409
Bacterial and virus strains		
*E. coli* BL21 λDE3	Lab stock	N/A
*E. coli* BL21 λDE3 pLysS (CamR)	Novogen	Cat#69451
HB101	Coli Genetic Stock Center at Yale	Cat#12554
MRE600	Lab stock	N/A
MG1655	Coli Genetic Stock Center at Yale	Cat#CGSC6300
Rosetta(DE3) pLysS	Sigma-Aldrich	Cat#70956
ΔssrA (MG1655 smpB_ssrA::kanR)	Saito et al.,2022^116^	SKEC114
ΔsmrB (MG1655 smrB::camR)	Saito et al.,2022^116^	SKEC120
ΔssrAΔsmrB (MG1655 smpB_ssrA::kanR smrB::camR)	Saito et al.,2022^116^	SKEC121
ΔsmrA (MG1655 smrA::camR)	Saito et al.,2022^116^	SKEC123
Chemicals, peptides, and recombinant proteins		
2-mercaptoethano	Sigma-Aldrich	Cat#M3148; CAS: 60-24-2
5′ Deadenylase	New England Biolabs	Cat#M0331S
^10^N-Formyltetrahydrofolate	Biosynth^®^ Carbosynth	Cat#FF165438; CAS: 2800-34-2
α-Lactose monohydrate	Sigma-Aldrich	Cat#L3625; CAS: 5989-81-1
Ammonium acetate, CH_3_CO_2_NH4	Thermo Fisher Scientific	Cat#BP3261; CAS: 631-61-8
Ammonium chloride, NH_4_Cl	Sigma-Aldrich	Cat#09718; CAS: 12125-02-9
Ammonium sulfate, (NH_4_)_2_SO_4_	Sigma-Aldrich	Cat#A4418; CAS: 7783-20-2
Ampicillin	Sigma-Aldrich	Cat#A0166; CAS: 69-53-4
Apyrase	New England Biolabs	Cat#M0393S
Ascorbic acid	Sigma-Aldrich	Cat#A5960; CAS: 50-81-7
ATP, [α−^32^P]	Perkin Elmer	Cat#BLU003H250U C
ATP, [γ−^32^P]	Perkin Elmer	Cat#NEG035C005M C
Azino-bis(3-Ethylbenzthiazoline-6-Sulfonic Acid), ABTS	Pierce	Cat#34026; CAS: 30931-67-0
Benzonase	Millipore	Cat#70746; CAS: 9025-65-4
BsaI-HF^®^	New England Biolabs	Cat#R3535L
Calcium chloride dihydrate	Sigma-Aldrich	Vat#C5080; CAS: 10035-04-8
Chloramphenicol	Sigma-Aldrich	Cat#C0378; CAS: 56-75-7
Chloroform	Sigma-Aldrich	Cat#C2432; CAS: 67-66-3
cOmpleteTM, EDTA-free protease inhibitor cocktail	Roche	Cat#11873580001
Creatine phosphate	Roche	Cat# 10621714001; CAS: 71519-72-7
Creatine Phosphokinase from rabbit muscle	Sigma-Aldrich	Cat#C3755
Cycloheximide	Sigma-Aldrich	Cat#C7698; CAS: 66-81-9
D-Biotin	Thermo Fisher Scientific	Cat#B20656; CAS: 58-85-5
Dithiothreitol, C_4_H_10_O_2_S_2_	Sigma-Aldrich	Cat#43815; CAS: 3483-12-3
Dimethyl sulfoxide, anhydrous	Sigma-Aldrich	Cat#276855; CAS: 67-68-5
*E. coli* RNA Polymerase, Core Enzyme	New England Biolabs	Cat#M0550S
1-ethyl-3-(3-dimethylaminopropyl)carbodiimide hydrochloride, EDC	Thermo Fisher Scientific	Cat#22980; CAS: 25952-53-8
Ethanol, 200-proof	Koptec	Cat#V1016; CAS: 64-17-5
Ethylenediaminetetraacetic acid, EDTA	Sigma-Aldrich	Cat#E6758; CAS: 60-00-4
Exonuclease I	Thermo Fisher Scientific	Cat#EN0581
Exonuclease III	Thermo Fisher Scientific	Cat#EN0191
Fidaxomicin	Apexbio Technology LLC.	Cat#B175550; CAS: 873857-62-6
Formamide (Deionized)	Sigma-Aldrich	Cat#F9037; CAS: 75-12-7
Fusidic acid	Sigma-Aldrich	Cat#F0756; CAS: 6990-06-3
GGGGDGDY-Lys(biotin)	Genscript	NA
Glucose, C_6_H_12_O_6_	Sigma-Aldrich	Cat#G8270; CAS: 50-99-7
Glycerol	Sigma-Aldrich	Cat#G5516; CAS: 56-81-5
Glycine	Sigma-Aldrich	Cat#50046; CAS: 56-40-6
Glycogen from mussels	Roche	Cat#10901393001; CAS: 9005-79-2
Guanosine 5′-diphosphate (GDP) sodium salt	Sigma-Aldrich	Cat#G7127; CAS: 43139-22-6
HEPES	Sigma-Aldrich	Cat# H4034; CAS: 7365-45-9
Horseradish peroxidase	Pierce	Cat#31490; CAS: 9003-99-0
Imidazole	Sigma-Aldrich	Cat#I5513; CAS: 288-32-4
Isopropyl β-D-1-thiogalactopyranoside, IPTG	Thermo Fisher Scientific	Cat#50-490-794; CAS: 367-93-1
Kanamycin sulfate	Sigma-Aldrich	Cat#60615; CAS: 70560-51-9
Lambda Phage DNA	New England Biolabs	Cat#N3011S; CAS: 91080-14-7
Magnesium acetate tetrahydrate, (CH3COO)_2_Mg•4H_2_O	Sigma-Aldrich	Cat#M5661; CAS: 16674-78-5
Magnesium chloride, MgCl_2_	Thermo Fisher Scientific	Cat# BP214; CAS: 7786-30-3
Magnesium sulfate, MgSO_4_	Sigma-Aldrich	Cat#M7506; CAS: 7487-88-9
MES hydrate	Sigma-Aldrich	Cat#M8250; CAS: 1266615-59-1
Myokinase from rabbit muscle	Sigma-Aldrich	Cat#M3003; CAS: 9013-02-9
Mueller Hinton Agar	Sigma-Aldrich	Cat#70191-100G
Phenol	Sigma-Aldrich	Cat#P4557; CAS: 108-95-2
Phenol:Chloroform:Isoamyl Alcohol 25:24:1	Sigma-Aldrich	Cat#P2069
Phosphocreatine	Sigma-Aldrich	Cat#P1937; CAS: 108321-17-1
Phusion HotStart II High Fidelity DNA Polymerase	Thermo Fisher Scientific	Cat#F537S
Phusion^®^ High-Fidelity DNA Polymerase	New England Biolabs	Cat#M0530S
Pierce^™^ NeutrAvidin Protein	Thermo Fisher Scientific	Cat#31000
Potassium acetate, KOAc	Thermo Fisher Scientific	Cat#BP364; CAS: 127-08-2
Potassium chloride, KCl	Sigma-Aldrich	Cat#P9541; CAS: 7447-40-7
Potassium hydroxide, KOH	Thermo Fisher Scientific	Cat#P250-1; CAS: 1310-58-3
Potassium phosphate dibasic, K_2_HPO_4_	Sigma-Aldrich	Cat#60353; CAS: 7758-11-4
Potassium phosphate monobasic, KH_2_PO_4_	Sigma-Aldrich	Cat#P9791; CAS: 7778-77-0
Potassium pyrophosphate, K_4_P_2_O_7_	Sigma-Aldrich	Cat#322431; CAS: 7320-34-5
Pseudouridimycin	AdipoGen	Cat# AGCN20316M005; CAS: 1566586-52-4
Putrescine dihydrochloride, NH_2_(CH_2_)_4_NH_2_•2HCl	Sigma-Aldrich	Cat#P5780; CAS: 333-93-7
Pyrophosphatase, Inorganic from baker’s yeast (S. cerevisiae)	Sigma-Aldrich	Cat#I1891; CAS: 9024-82-2
RecJ_f_	New England Biolabs	Cat#M0264S
RNaseOUT^™^ Recombinant Ribonuclease Inhibitor	Thermo Fisher Scientific	Cat#10777
rNTPs	Promega	Cat#E6000
SequaGel UreaGel 29:1 Concentrate	National Diagnostics	Cat#EC-828
Sodium azide	Sigma-Aldrich	Cat#71289; CAS: 26628-22-8
Sodium chloride, NaCl	Sigma-Aldrich	Cat#S9888; CAS: 7647-14-5
Sodium dodecyl sulfate, SDS	Sigma-Aldrich	Cat#L5750; CAS: 151-21-3
Sodium phosphate dibasic, Na_2_HPO_4_	Sigma-Aldrich	Cat#S3264; CAS: 7558-79-4
Spermidine trihydrochloride	Sigma-Aldrich	Cat#85578; CAS: 334-50-9
Sucrose	Sigma-Aldrich	Cat#S5016; CAS: 57-50-1
SuperScript^™^ IV Reverse Transcriptase	Thermo Fisher Scientific	Cat#18090010
SYBR Gold Nucleic Acid Gel Stain	Thermo Fisher Scientific	Cat#S-11494
T4 DNA Ligase (2,000,000 units/ml)	New England Biolabs	Cat#M0202T
T4 DNA Ligase (400,000 units/ml)	New England Biolabs	Cat#M0202S
T4 Polynucleotide Kinase	New England Biolabs	Cat#M0201S
T4 RNA Ligase 2, truncated K227Q	New England Biolabs	Cat#M0351S
Terrific Broth	Thermo Fisher Scientific	Cat#BP2468500
Tetracycline hydrochloride	Sigma-Aldrich	Cat#T7660; CAS: 64-75-5
Total tRNA from MRE600	Roche	Cat#10109541001
Tris(2-carboxyethyl)phosphine hydrochloride, TCEP	Sigma-Aldrich	Cat#646547; CAS: 51805-45-9
Tris Base	Genesee Scientific	Cat#18-146; CAS: 77-86-1
tRNA^fMet^	tRNAprobes	Cat#FM-03
Trolox	Sigma-Aldrich	Cat#238813; CAS: 53188-07-1
Tryptone (Gibco^™^ Bacto^™^)	Thermo Fisher Scientific	Cat#DF0123-17-3; CVAS: 91079-40-2
TWEEN^®^ 20	Sigma-Aldrich	Cat#P9416; CAS: 9005-64-5
Ultra Low Range DNA Marker	Thermo Fisher Scientific	Cat#SM1213
Yeast extract	Sigma-Aldrich	Cat#Y1625; CAS: 8013-01-2
Critical commercial assays		
5′ DNA Adenylation Kit	New England Biolabs	Cat#E2610S
L-Amino acids	Sigma-Aldrich	Cat#LAA21
MEGAscript^®^ T7 Kit	Thermo Fisher Scientific	Cat#AM1334M
Select-a-Size^™^ DNA MagBead Kit	Zymo Research	Cat#D4084
Deposited data		
Mendeley Data: Raw high-throughput sequencing data for determining transcription fidelity by *E. coli* RNAP	This paper	dx.doi.org/10.17632/ ysc6r3dz2m.1
Recombinant DNA		
pBSM	Addgene	67505
pET His6 TEV LIC cloning vector (1B)	Addgene	29653
pET LIC cloning vector (2A-T)	Addgene	29655
pET His6 LIC cloning vector (2B-T)	Addgene	29666
pET His6 LIC cloning vector (2Bc-T)	Addgene	37236
TEV protease, S219V mutant	Addgene	pRK793
Evolved sortase (eSrtA), P94R/D160N/D165A/K190E/K196T. ${\text{His}}_{6}$ at the C-terminus of eSrtA.	Chen et al., 2011^131^	pET29-eSrtA
Wild type *E. coli* RNAP with RRAS (PKA tag) + LPETG (sortag) + ${\text{His}}_{6}$ tag at the C-terminus of ${\text{β}}^{\prime }$ subunit	This paper	pIA1234
Elongation factor G with ${\text{His}}_{6}$ tag at the N-terminus	This paper	pCK-EF-G
Elongation factor Tu with ${\text{His}}_{6}$ tag at the N-terminus	This paper	pCK-EF-Tu
Elongation factor Ts with ${\text{His}}_{6}$ tag at the N-terminus	This paper	pCK-EF-Ts
Initiation factor 1 with ${\text{His}}_{6}$ tag at the C-terminus	This paper	pET24b-IF1
Initiation factor 2 with ${\text{His}}_{6}$ tag at the C-terminus	This paper	pET24b-IF2
Initiation factor 3 with ${\text{His}}_{6}$ tag at the C-terminus	This paper	pET24b-IF3
Methionyl-tRNA formyltransferase with ${\text{His}}_{6}$ tag at the C-terminus	This paper	pET2Bc-T-fmt
Nucleotide diphosphate kinase with ${\text{His}}_{6}$ tag at the N-terminus	This paper	pET2B-T-ndk
GreA with ${\text{His}}_{6}$ tag at the C-terminus	This paper	pET2Bc-T-GreA
GreB with ${\text{His}}_{6}$ tag at the C-terminus	This paper	pET2Bc-T-GreB
RelE with ${\text{His}}_{6}$ tag at the N-terminus	This paper	pET22b-Δ9-His6xRelB:RelE
Alanyl-tRNA synthetase with ${\text{His}}_{6}$ tag at the N-terminus	This paper	pET2B-T-alaS
Arginyl-tRNA synthetase with ${\text{His}}_{6}$ tag at the N-terminus	This paper	pET2B-T-argS
Asparaginyl-tRNA synthetase with ${\text{His}}_{6}$ tag at the N-terminus	This paper	pET2B-T-asnS
Aspartate-tRNA synthetase with ${\text{His}}_{6}$ tag at the C-terminus	This paper	pJL-H6-aspS
Cysteinyl-tRNA synthetase with ${\text{His}}_{6}$ tag at the C-terminus	This paper	pJL-H6-cysS
Glutaminyl-tRNA synthetase with ${\text{His}}_{6}$ tag at the C-terminus	This paper	pJL-H6-glnS
Glutamyl-tRNA synthetase with His6 tag at the C-terminus	This paper	pJL-H6-gltX
Glycyl-tRNA synthetase with ${\text{His}}_{6}$ tag at the C-terminus of β subunit	This paper	pJL-H6-glyQS
Histidyl-tRNA synthetase with ${\text{His}}_{6}$ tag at the C-terminus	This paper	pJL-H6-hisS
Isoleucyl-tRNA synthetase with ${\text{His}}_{6}$ tag at the N-terminus	This paper	pET2B-T-ileS
Leucyl-tRNA synthetase with ${\text{His}}_{6}$ tag at the C-terminus	This paper	pJL-H6-leuS
Lysyl-tRNA synthetase with ${\text{His}}_{6}$ tag at the C-terminus	This paper	pJL-H6-lysS
Methionyl-tRNA synthetase with ${\text{His}}_{6}$ tag at the C-terminus	This paper	pJL-H6-metG
Phenylalanyl-tRNA synthetase with ${\text{His}}_{6}$ tag at the N-terminus of the α subunit	This paper	pET2B-T-pheS
Prolyl-tRNA synthetase with ${\text{His}}_{6}$ tag at the C-terminus	This paper	pJL-H6-proS
Seryl-tRNA synthetase with ${\text{His}}_{6}$ tag at the C-terminus	This paper	pJL-H6-serS
Threonyl-tRNA synthetase with ${\text{His}}_{6}$ tag at the N-terminus	This paper	pET2B-T-thrS
Tryptophanyl-tRNA synthetase with ${\text{His}}_{6}$ tag at the C-terminus	This paper	pJL-H6-trpS
Tyrosyl-tRNA synthetase with ${\text{His}}_{6}$ tag at the C-terminus	This paper	pJL-H6-tyrS
Valyl-tRNA synthetase with ${\text{His}}_{6}$ tag at the C-terminus	This paper	pJL-H6-valS
*pyrL* leader sequence with downstream λ phage sequence	This paper	pUC19-*pyrL*-TC4
tRNA^fMet^ isoform 1 with *lpp* promoter inserted into pBSM between XhoI and PstI sites	This paper	pBSM-lpp-tRNA^fMet^
Pyranose oxidase from *Trametes multicolor* with ${\text{His}}_{6}$ tag at the C-terminus	This paper	p-PO
Software and algorithms		
Igor Pro 7	WaveMetrics	https://www.wavemetrics.com/downloads/current
R	RStudio	https://www.rstudio.com
ImageQuant TL 8.2	Cytiva	https://www.cytivalifesciences.com/en/us/shop/protein-analysis/molecular-imaging-for-proteins/imaging-software/imagequant-tl-8-2-imageanalysis-software-p09518
Jupyter Notebook	Anaconda Navigator	https://docs.anaconda.com/anaconda/navigator/
Microsoft Office	Microsoft	https://www.microsoft.com/enus/microsoft-365/microsoft-office
Adobe Photoshop and Illustrator	Adobe	https://www.adobe.com
Matlab	MathWorks	https://www.mathworks.com/products/matlab.html
deML	Renaud et al., 2015^160^	https://github.com/grenaud/deML
HTStream	Petersen et al., 2015^189^	https://github.com/s4hts/HTStream
Calib	Orabi et al., 2019^161^	https://github.com/vpc-ccg/calib
Mafft	Katoh and Standley, 2013^165^	https://mafft.cbrc.jp/alignment/software/
Biopython 1.19.4	Cock et al., 2009^190^	https://biopython.org
Logomaker	Tareen and Kinney, 2020^191^	https://logomaker.readthedocs.io/en/latest/examples.html#splice-site-probability-logo
COOT v0.8.3	Emsley and Cowtan, 2004^192^	https://www2.mrc-lmb.cam.ac.uk/personal/pemsley/coot/
cryoSPARC v3.1	Punjani et al., 2017^170^	https://cryosparc.co m/
Gctf	Zhang, 2016^173^	https://www2.mrc-lmb.cam.ac.uk/research/locally-developed-software/zhang-software/#gctf
MotionCor2	Zheng et al., 2017^172^	https://emcore.ucsf.edu/ucsf-software
PHENIX	Adams et al., 2010^193^	https://www.phenix-online.org/documentation/
RELION v3.1	Zivanov et al., 2018^169^	https://www2.mrclmb.cam.ac.uk/relion
UCSF Chimera v 1.13.1	Pettersen et al., 2004^174^	https://www.cgl.ucsf.edu/chimera
UCSF ChimeraX v1.0	Goddard et al., 2018^194^	https://www.cgl.ucsfedu/chimerax/
PyMOL v1.6	Schrödinger and DeLano, 2020^195^	https://pymol.org/2/
LocScale	Jakobi et al., 2017^177^	https://git.embl.de/jakobi/LocScale
Single-molecule and high throughput sequencing analysis pipeline	This paper	https://zenodo.org/record/6534021#.YntNty8Rrxg
Other		
10% carboxyl polystyrene 1.0 μm beads (w/w)	Bangs Laboratories, Inc.	Cat#PC04001
1260 Infinity HPLC system	Agilent	https://www.agilent.com/en/product/liquid-chromatography/hplc-systems/analytical-hplc-systems
Amicon Ultra-0.5 Centrifugal Filter Unit, 10K MWCO	Thermo Fisher Scientific	Cat#UFC501024
Amicon Ultra-0.5 Centrifugal Filter Unit, 3K MWCO	Thermo Fisher Scientific	Cat#UFC500324
Amicon Ultra-15 Centrifugal Filter Unit, 10K MWCO	Thermo Fisher Scientific	Cat#UFC901024
Amicon Ultra-15 Centrifugal Filter Unit, 30K MWCO	Thermo Fisher Scientific	Cat#UFC903008
Amicon Ultra-15 Centrifugal Filter Unit, 3K MWCO	Thermo Fisher Scientific	Cat#UFC900308
Avanti JXN-26 centrifuge	Beckman Coulter Life Sciences	Cat#
C-flat CF-1.2/1.3 400 mesh copper grids	Protochips, Inc	Cat#CF-1.2/1.3-4CU-50
Dynabeads^®^ M-280 Streptavidin	Thermo Fisher Scientific	Cat#11205D
Dynabeads^™^ Protein G	Thermo Fisher Scientific	Cat#10003D
Econospin	Epoch Life Science	Cat#1920-050/250
EmulsiFlex-C5	Avestin	https://www.avestin.com/emulsiflexc5.htm
HiPrep^™^ Sephacryl S100 16/60	GE Healthcare	Cat#17-1165-01
HiPrep^™^ Sephacryl S300 16/60	GE Healthcare	Cat#17-1167-01
HisTrap HP^™^	GE Healthcare	Cat#17-5248-02
HiTrap^®^ DEAE Fast Flow	GE Healthcare	Cat#17-5055-01
HiTrap^®^ Desalting	GE Healthcare	Cat#45-000-252
HiTrap^®^ Heparin HP	GE Healthcare	Cat#17-0407-01
HiTrap^®^ Q HP	GE Healthcare	Cat#17-1154-01
HiTrap^®^ SP HP	GE Healthcare	Cat#17-1152-01
JA-20 Fixed-Angle Aluminum Rotor	Beckman Coulter Life Sciences	Cat#334831
Janelia Fluor^®^ 549, Maleimide	Tocris	Cat#6500
J-LITE JLA-8.1000 Fixed-Angle Aluminum Rotor	Beckman Coulter Life Sciences	Cat#363688
Millex-GV Filter (0.22 μm)	Millipore	Cat#SLGV004SL
Mono Q^®^ 5/50 GL column	GE Healthcare	Cat#GE17-5166-01
Nalgene^™^ Oak Ridge	Thermo Fisher Scientific	Cat#3119-0050
Ni-NTA Agarose	Qiagen	Cat#30210
Oligo Clean & Concentrator	Zymo Research	Cat#D4060
Open-Top Thick wall Polycarbonate	Beckman Coulter Life Sciences	Cat#355631
Polycarbonate Bottle with cap assembly	Beckman Coulter Life Sciences	Cat#355655 (bottle) Cat#355623 (cap)
Polypropylene Bottle Assembly	Beckman Coulter Life Sciences	Cat#A98813
S3000 Ultrasonic Liquid Processor	Misonix	Cat#EW-04711-81
SpectraPor^®^ dialysis membrane	Repligen	https://www.repligen.com/technologies/dialysis/spectrapor-biotech-grade-dialysis-tubing-and-membranes/spectrapor-1-5-dry-standard-grade-regenerated-cellulose-rc-dialysis-tubing-trial-kits
Steriflip	Thermo Fisher Scientific	Cat#SCGP00525
Streptavidin Magnetic Beads	Thermo Fisher Scientific	Cat#88816
SW 32 Ti Rotor	Beckman Coulter Life Sciences	Cat#369650
Syringe filter, PVDF, 0.22 μm	Genesee Scientific	Cat#25-243
Type 45 Ti Rotor	Beckman Coulter Life Sciences	Cat#339160
UltraPure^™^ DNase/RNase-Free Distilled Water	Thermo Fisher Scientific	Cat#10977-023
XBridge BEH C18 Column, 130Å pore size, 5 μm particle size, 4.6 mm (inner diameter) X 150 mm (length)	Waters^™^	Cat#186003116

## Highlights

- Translation reduces pausing and termination of transcription during coupling
- The ribosome increases the activity and reduces the fidelity of RNA polymerase
- An increased in misincorporation and error tolerance reduces transcript fidelity
- Mechanical force and allostery explains the effects of coupling on transcription

## INCLUSION AND DIVERSITY

One or more of the authors of this paper self-identifies as an underrepresented ethnic minority in their field of research or within their geographical location.
