# Supplementary material for: A trailing ribosome speeds up RNA polymerase at the expense of transcript fidelity via force and allostery
Source: Cell. Author manuscript; Available in PMC 2023 Apr 27. (PMC10135430; doi:10.1016/j.cell.2023.02.008)
Supplement: 1 — Data S1. Cryo-EM data processing, related to Figure 6. [file NIHMS1881320-supplement-1.pdf]

Data S1

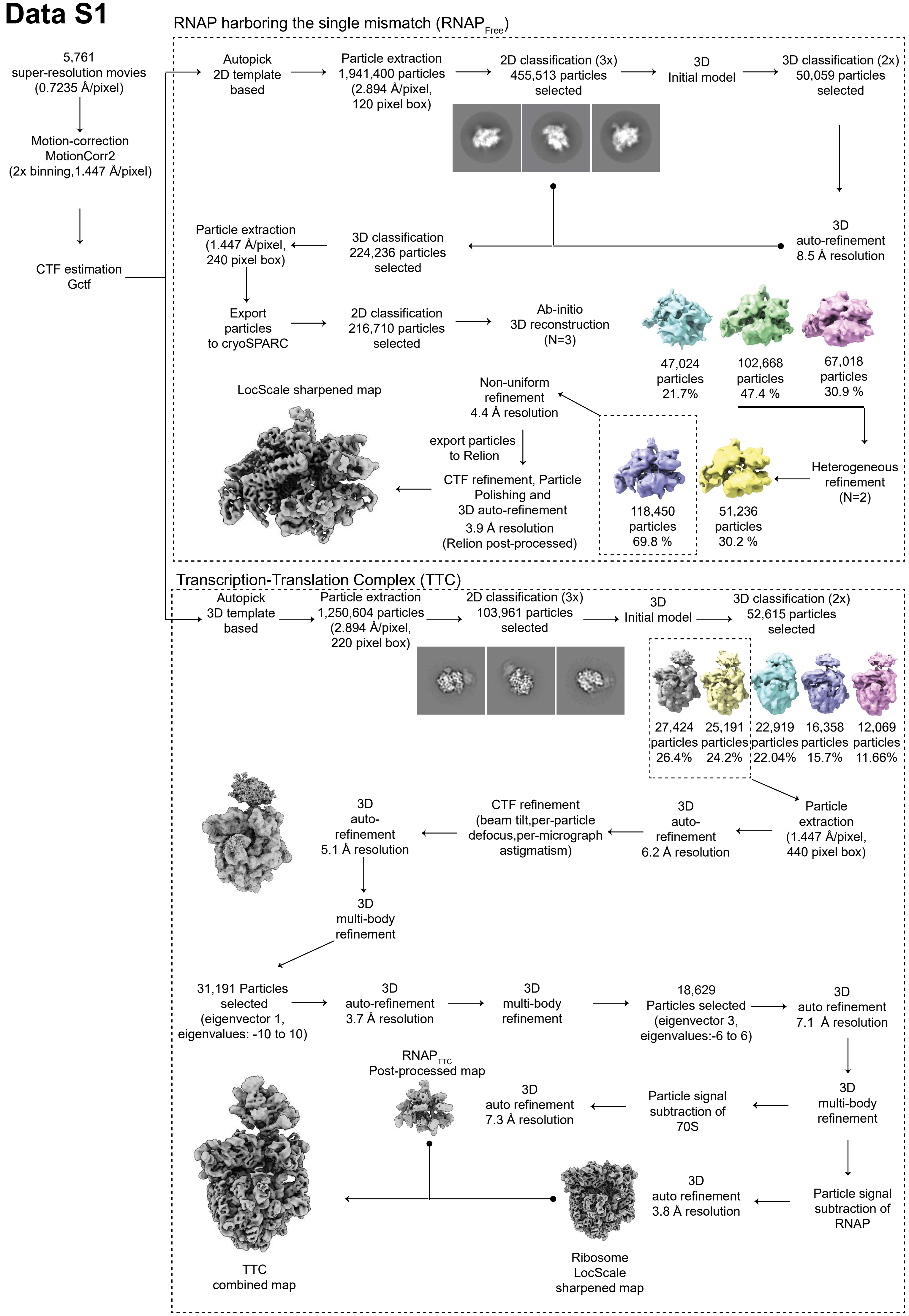

RNAP harboring the single mismatch (RNAP$_{Free}$)
5,761 super-resolution movies (0.7235 Å/pixel)
Motion-correction MotionCorr2 (2x binning,1.447 Å/pixel)
CTF estimation Gctf
Autopick 2D template based
Particle extraction 1,941,400 particles (2.894 Å/pixel, 120 pixel box)
2D classification (3x) 455,513 particles selected
3D Initial model
3D classification (2x) 50,059 particles selected
3D auto-refinement 8.5 Å resolution
3D classification 224,236 particles selected
Particle extraction (1.447 Å/pixel, 240 pixel box)
Export particles to cryoSPARC
2D classification 216,710 particles selected
Ab-initio 3D reconstruction (N=3)
47,024 particles 21.7%
102,668 particles 47.4 %
67,018 particles 30.9 %
Heterogeneous refinement (N=2)
51,236 particles 30.2 %
118,450 particles 69.8 %
Non-uniform refinement 4.4 Å resolution
export particles to Relion
CTF refinement, Particle Polishing and 3D auto-refinement
3.9 Å resolution (Relion post-processed)
LocScale sharpened map
Transcription-Translation Complex (TTC)
Autopick 3D template based
Particle extraction 1,250,604 particles (2.894 Å/pixel, 220 pixel box)
2D classification (3x) 103,961 particles selected
3D Initial model
3D classification (2x) 52,615 particles selected
27,424 particles 26.4%
25,191 particles 24.2%
22,919 particles 22.04%
16,358 particles 15.7%
12,069 particles 11.66%
Particle extraction (1.447 Å/pixel, 440 pixel box)
3D auto-refinement 6.2 Å resolution
CTF refinement (beam tilt,per-particle defocus,per-micrograph astigmatism)
3D auto-refinement 5.1 Å resolution
3D multi-body refinement
31,191 Particles selected (eigenvector 1, eigenvalues: -10 to 10)
3D auto-refinement 3.7 Å resolution
3D multi-body refinement
18,629 Particles selected (eigenvector 3, eigenvalues:-6 to 6)
3D auto refinement 7.1 Å resolution
3D multi-body refinement
Particle signal subtraction of RNAP
3D auto refinement 3.8 Å resolution
Ribosome LocScale sharpened map
Particle signal subtraction of 70S
3D auto refinement 7.3 Å resolution
RNAP$_{TTC}$ Post-processed map
TTC combined map

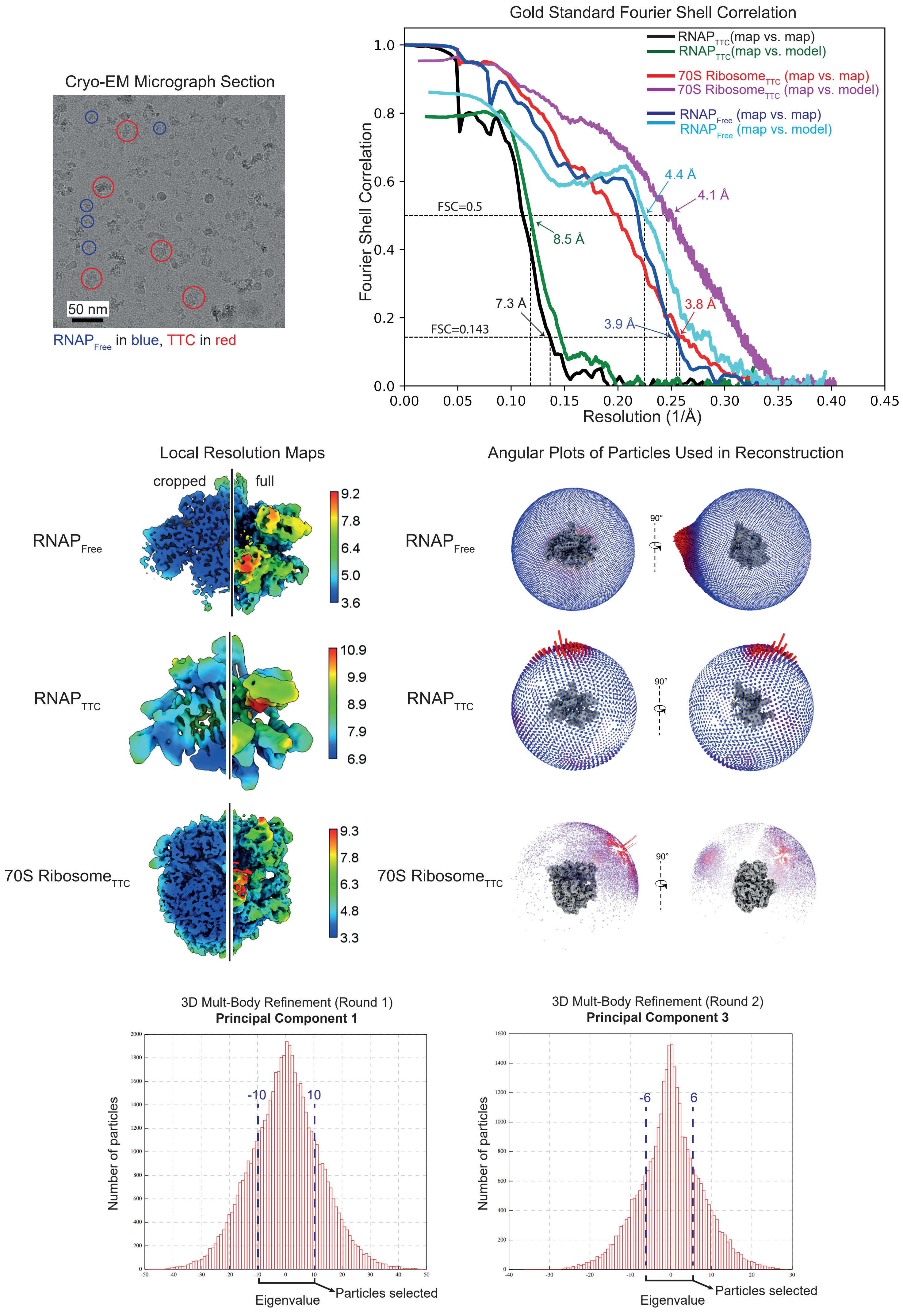

Cryo-EM Micrograph Section
50 nm
RNAP Free in blue, TTC in red
Gold Standard Fourier Shell Correlation
RNAP TTC (map vs. map)
RNAP TTC (map vs. model)
70S Ribosome TTC (map vs. map)
70S Ribosome TTC (map vs. model)
RNAP Free (map vs. map)
RNAP Free (map vs. model)
Fourier Shell Correlation
Resolution (1/Å)
FSC=0.5
FSC=0.143
8.5 Å
7.3 Å
4.4 Å
4.1 Å
3.8 Å
3.9 Å
Local Resolution Maps
cropped
full
RNAP Free
RNAP TTC
70S Ribosome TTC
Angular Plots of Particles Used in Reconstruction
90°
3D Mult-Body Refinement (Round 1)
Principal Component 1
3D Mult-Body Refinement (Round 2)
Principal Component 3
Number of particles
Eigenvalue
Particles selected
-10
10
-6
6
