# Supplementary figures and images for: A trailing ribosome speeds up RNA polymerase at the expense of transcript fidelity via force and allostery
Source: Cell. Author manuscript; Available in PMC 2023 Apr 27. (PMC10135430; doi:10.1016/j.cell.2023.02.008)

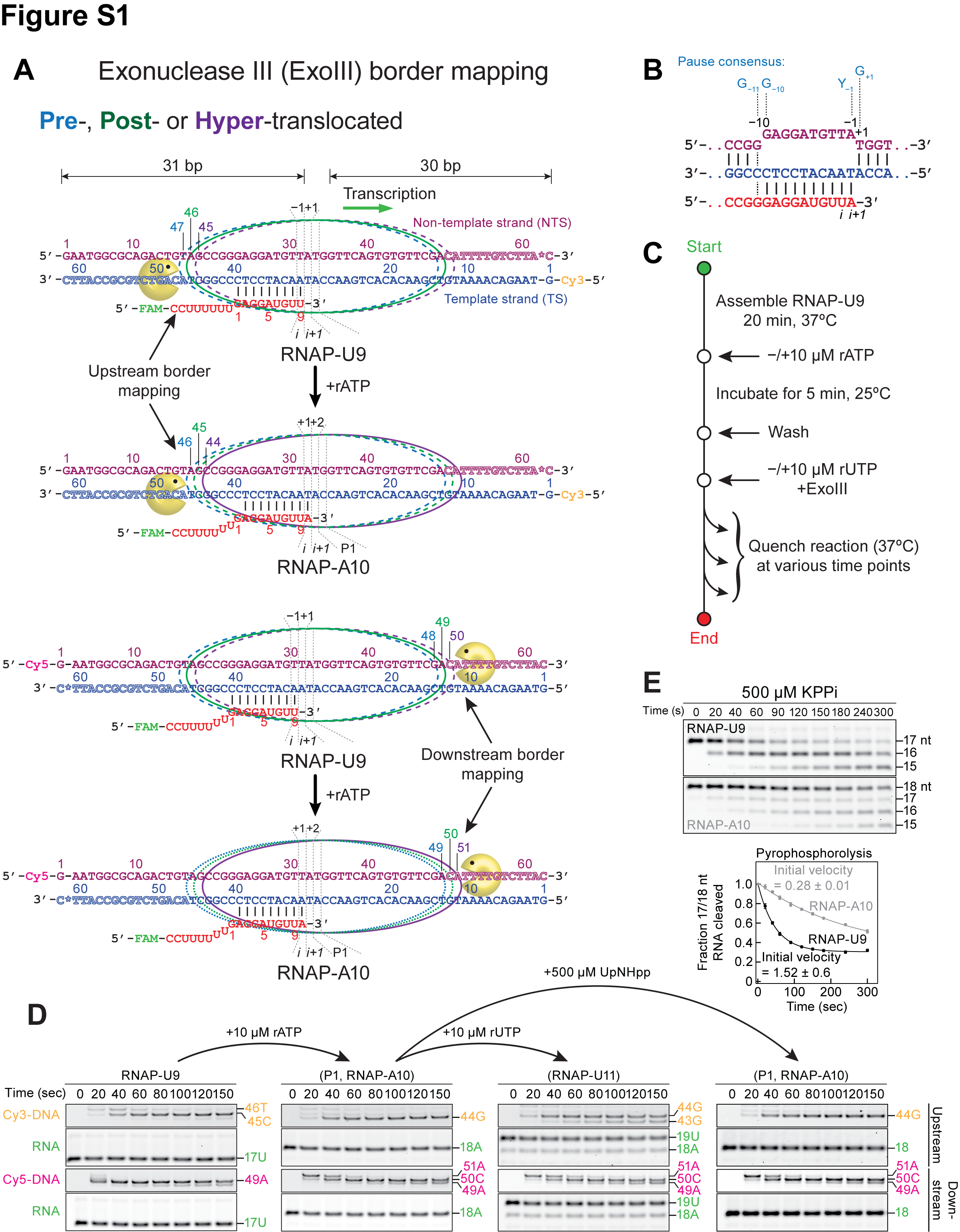

Supplement: 2 — Figure S1. Characterization of Pause 1 (P1) through border mapping by ExoIII digestion, related to Figure 1 and STAR Methods (A) Schematics of upstream border mapping (top panels) and downstream border mapping (bottom panels) of RNAP. Each panel indicates border mappings for the reference polymerase (RNAP-U9) and after the enzyme has translocated by one base pair (RNAP-A10). The outlines in blue, green and purple denote the various footprints for RNAP in the pre-, post-, and hyper-translocated states respectively. Lines at the queried border denote the expected labeled DNA fragment lengths after ExoIII digestion, which correspond to the three states. (B) Diagram showing the RNA-DNA hybrid base pairing of a pre-translocated RNAP at P1. The G−11G−10Y−1G+1 elemental pause motif is shown above the sequences. (C) Experimental workflow of the ExoIII border mapping experiment. Step-wise bubble assembly of an elongating RNAP gives the reference enzyme (RNAP-U9), which can be sequentially stepped into RNAP-A10 and RNAP-U11. Then, ExoIII is added to query either the upstream or the downstream borders over time. (D) DNA template borders as mapped by ExoIII digestion at U9, A10, and U11 (first three panels). Right panel, nonhydrolyzable rUTP analog UpNHpp fails to return a hyper-translocated RNAP-A10 to a post-translocated A10. (E) The hyper-translocated RNAP-A10 is less prone to pyrophosphorolysis than the post-translocated RNAP-U9, which shows a higher rate of RNA shortening in the presence of potassium pyrophosphate (KPPi). [file NIHMS1881320-supplement-2.tif]

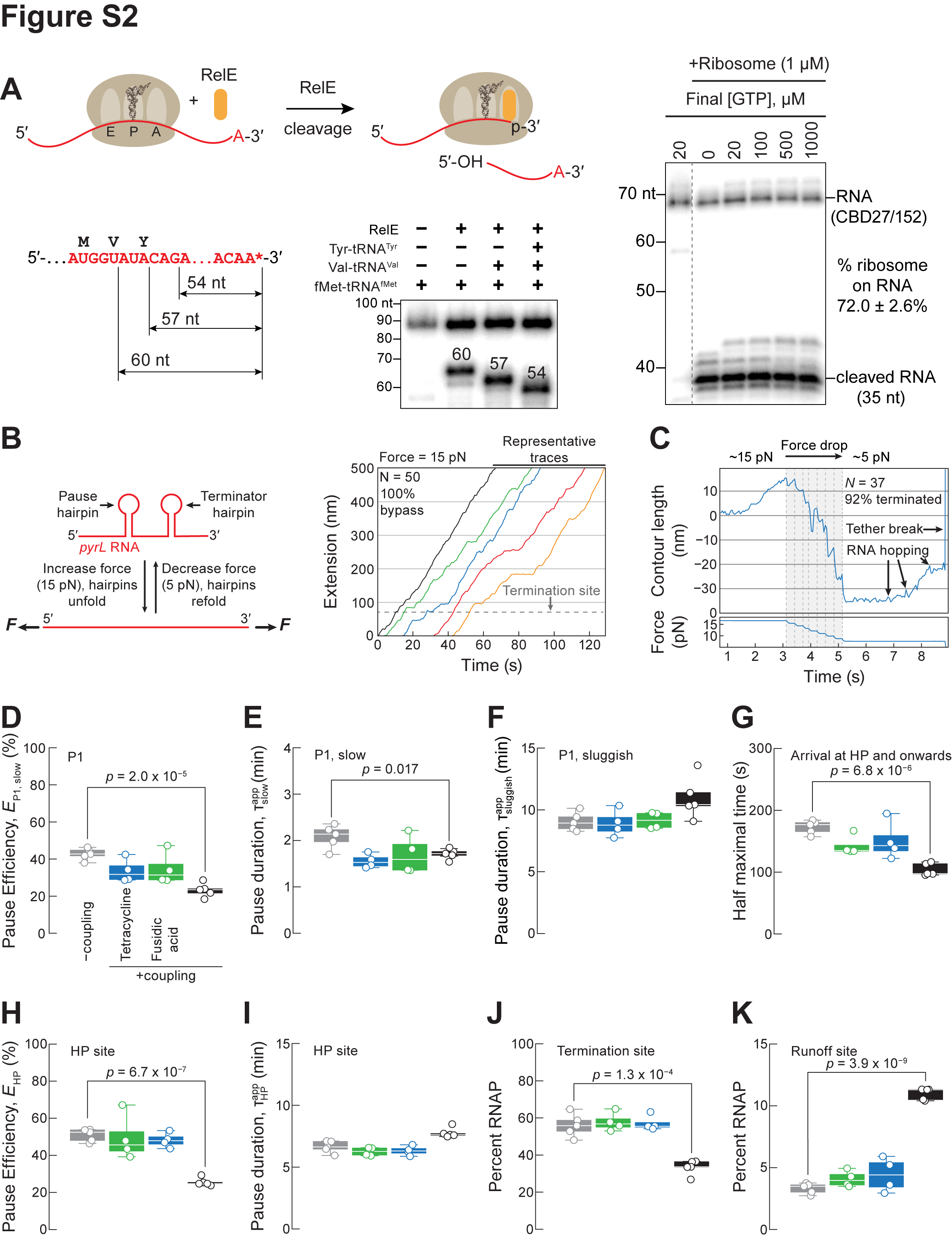

Supplement: 3 — Figure S2. Active translation speeds up coupled transcription, related to Figure 2 (A) RelE binds a ribosome with an empty A-site and cleaves the underlying RNA between the second and the third nucleotide of the codon (left panel). The loading efficiency of the ribosome on the RNA, determined by RelE cleavage, is ~72.0 ± 2.6% (right panel). The lane for −ribosome control and the lanes for +ribosome incubated with increasing concentrations of rGTP were spliced together from the same piece of gel. The splicing interface is denoted by the dotted line. (B) The application of a high (15 pN) force by optical tweezers unfolds RNA hairpins and vice versa (left panel). RNAP avoids transcription termination when the terminator hairpin unwinds (right panel). Representative traces are shown in different colors. (C) A rapid force drop from 15 pN to 5 pN allows the terminator hairpin to refold, which will terminate transcription. (D–K) Translation elongation inhibitors such as tetracycline (blue) and fusidic acid (green) suppressed the effects of the ribosome on the pause efficiencies, overall velocity, termination and runoff probabilities of RNAP during coupling. Data are mean ± SD for four independent experiments. [file NIHMS1881320-supplement-3.tif]

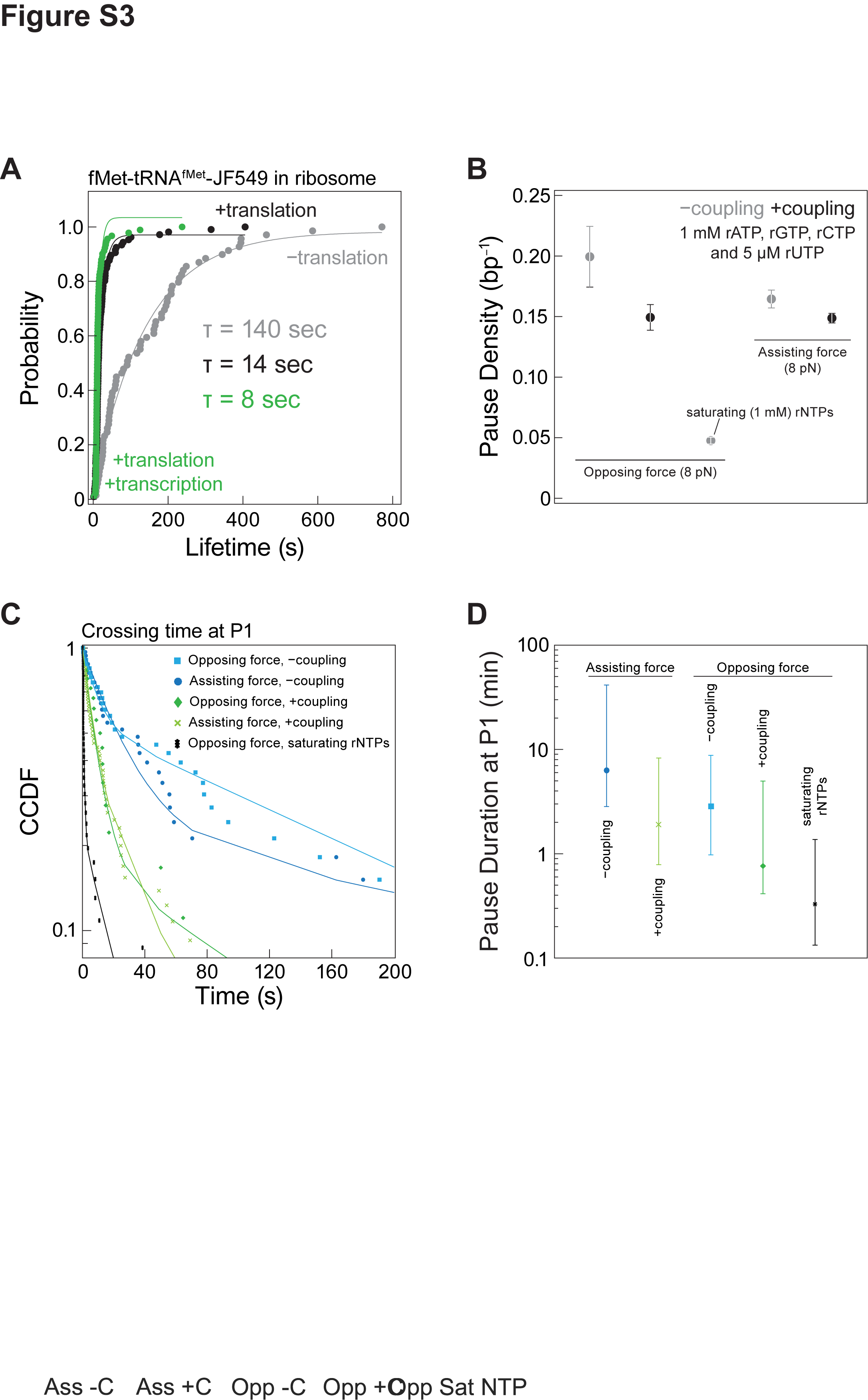

Supplement: 4 — Figure S3. Coupling reduces transcription pause density and duration, related to Figure 3 (A) Fluorescent lifetime of fMet-tRNAfMet-JF549 in the ribosome is an order longer in a stationary ribosome than of a translocating ribosome. This indicates that the measurement of its lifetime (dwell time of fMet-tRNAfMet-JF549 in the ribosome before dissociating) in translocating ribosome is not limited by its photobleaching time. (B) Transcription pause densities (number of pauses > 0.5 s per bp) in opposing and in assisting force setup under −coupling (grey) and +coupling (black) conditions. (C) Crossing times for RNAP at P1 are shown for each condition, along with a biexponential fit. The fit parameters are in Table S2C. (D) Plots of the pause durations in each condition taken from the fits in (C). [file NIHMS1881320-supplement-4.tif]

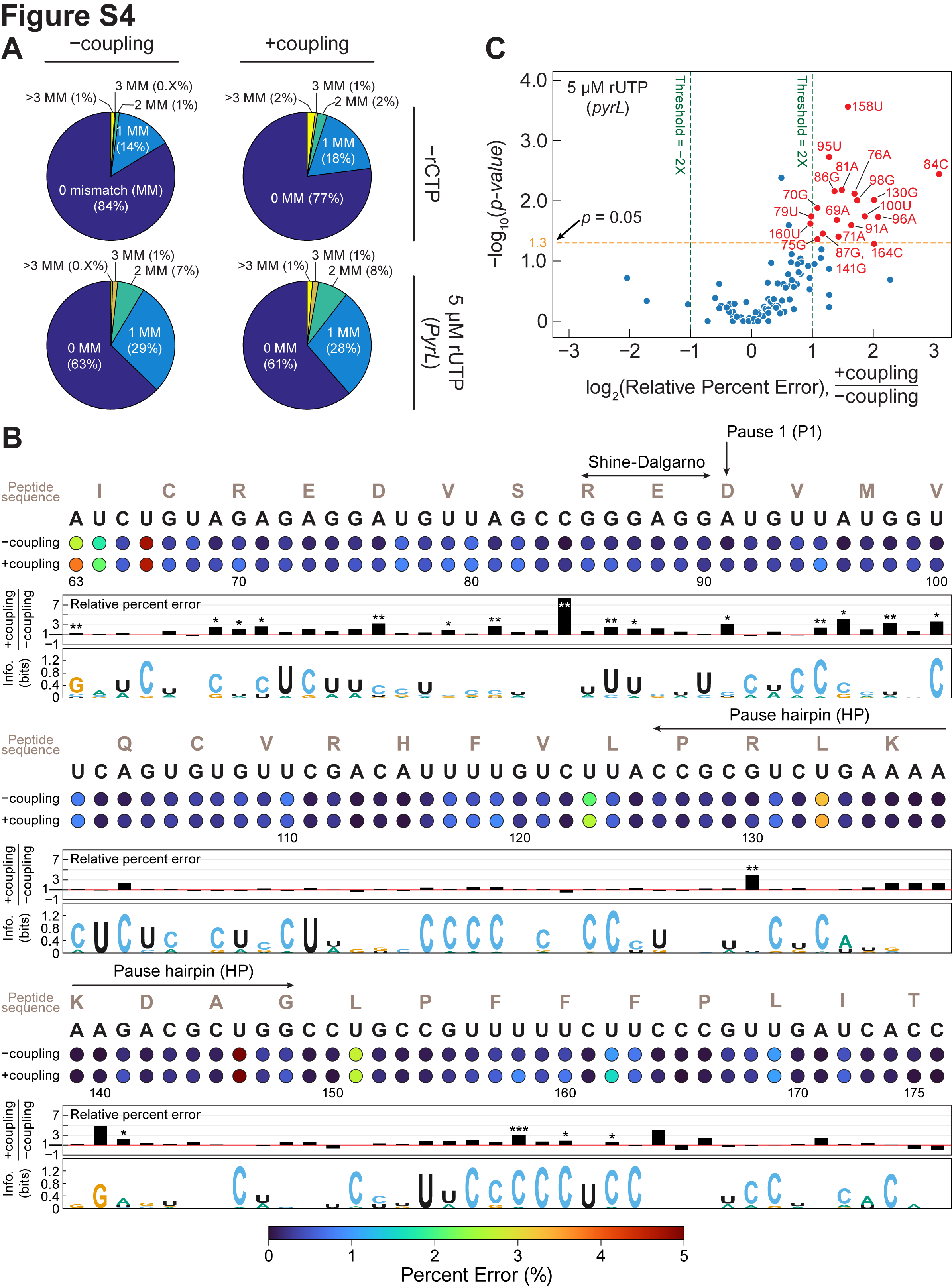

Supplement: 5 — Figure S4. RNAP tends to misincorporate when coupled to the ribosome, related to Figure 4 (A) The percentage of RNAs that contain 0, 1, 2, 3 and >3 mismatches (MM) for −coupling and +coupling reactions (left and right pie charts) for the fidelity assay (top pie charts) and for transcription on pyrL with limiting rUTP (bottom pie charts). (B) Along pyrL and regardless of coupling, RNAP tends to misincorporate at uridine sites that are immediately preceded by cytidine sites. At these U sites, cytidine is preferentially incorporated. (C) RNAP shows a higher misincorporation rate along pyrL when coupled to the ribosome given that more sites occupy the right region of the volcano plot that corresponds to positive relative percent error when comparing between the +coupling and the −coupling reactions. Of the 114 positions surveyed, 20 contained more mistakes in the +coupling reaction than in the −coupling reaction, which are statistically significant (Fisher exact test, p-value < 0.05). These 20 positions are highlighted in red in the volcano plot. At these positions, the misincorporated ribonucleotides are mostly uridine or cytidine. [file NIHMS1881320-supplement-5.tif]

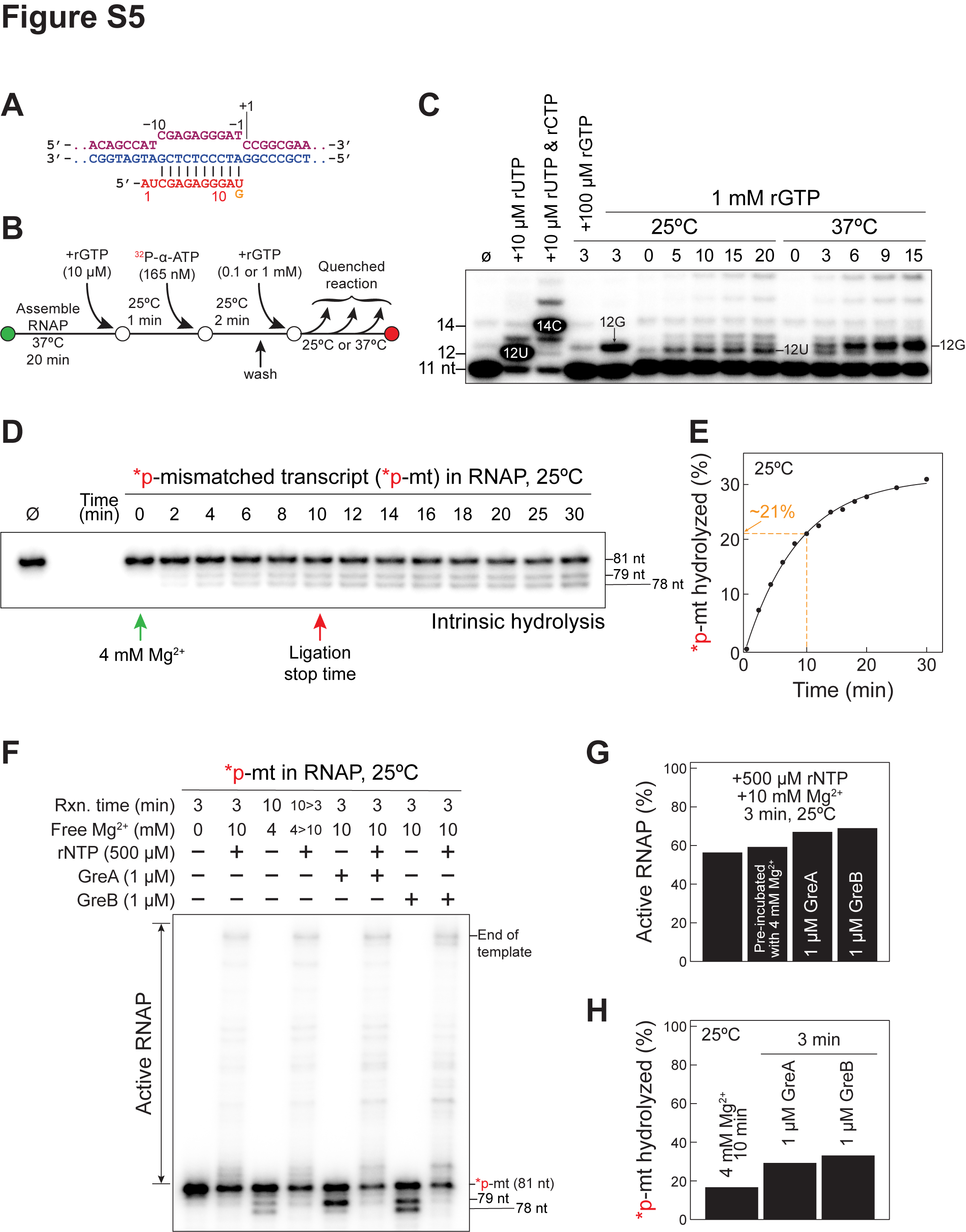

Supplement: 6 — Figure S5. The ribosome prevents error rectification by RNAP during coupling, related to Figure 5 (A) A schematic depicting the bubble construct to test dinucleotide cleavage (error rectification) by RNAP after misincorporation of rGTP at the −1 position. (B) The experimental workflow to measure error rectification by RNAP. (C) Reaction at 37°C drives more rGTP misincorporation (12G) by RNAP than at 25°C. (D) Time-dependent intrinsic hydrolysis of RNA by RNAP bearing a terminal mismatch (*p-mt; simulating ES56) using the bubble construct shown in Figure 4A. The transcription elongation complex (RNAP is 10-fold in excess of RNA and DNA templates) was assembled in the absence of Mg2+, and the hydrolysis of *p-mt was monitored when 4 mM Mg2+ was added. Di- and tri-nucleotide cleavage from the terminal end of *p-mt predominates, resulting in 79 nt and 78 nt RNA species respectively. (E) Quantification of (D) showing the percentage of *p-mt hydrolyzed with time in the presence of 4 mM Mg2+. A ligation time of 10 min for attaching DNA handles to RNAP was selected, which correspond to ~21% of RNAP with its RNA cleaved. (F) RNAP carrying RNA with a terminal rG-dA mismatch was incubated with saturating rNTPs (500 μM each) for 3 min to determine the percent active enzyme (the percentage of RNAP that had extended its RNA beyond 81 nt), which was increased in the presence of Gre factors. There is limited hydrolysis of RNA carrying the terminal mismatch after 10 min of incubation in 4 mM Mg2+ (lane 3). Lane 4 shows RNAP that was subjected to an additional 3 min incubation with 500 μM rNTPs after 10 min of preincubation (10>3) in 4 mM Mg2+ that was increased to 10 mM (4>10) at the same time when rNTPs were introduced. The cleavage of *p-mt to remove the offending ribonucleotide through intrinsic hydrolysis by RNAP is inefficient (lane 3) and can be improved with the assistance of GreA and GreB (lanes 5 and 7). GreA cleaves *p-mt to produce mainly dinucleotide fragments (lane 5) w [file NIHMS1881320-supplement-6.tif]

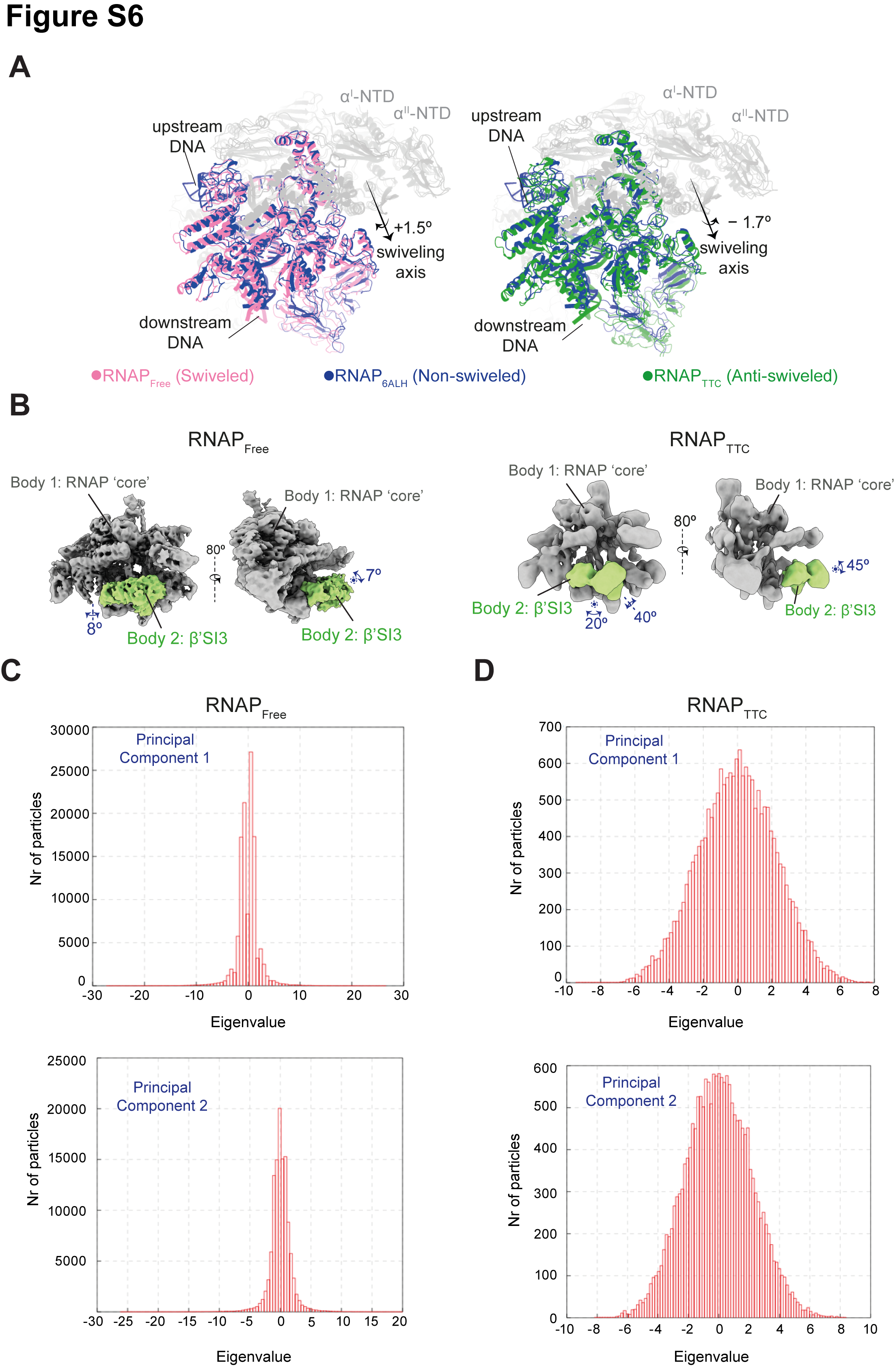

Supplement: 7 — Figure S6. Extent of swiveling found in RNAPFree and RNAPTTC, and focused β′SI3 multibody analysis, related to Figure 6 (A) Left: RNAPFree versus RNAP6ALH models. Coordinates of both structures were aligned relative to the core module of RNAP (gray ribbon) and it was observed that the swivel module of RNAPFree (‘swiveled’, hot pink) was rotated +1.5° towards a swiveled direction relative to its location in the RNAP6ALH (‘non-swiveled’, blue). Right: RNAPTTC versus RNAP6ALH models. Coordinates of both structures were aligned relative to the core module (gray ribbon), and it was observed that the swivel module of RNAPTTC (‘anti-swiveled’, green) was rotated −1.7° towards an anti-swiveled direction relative to its location in the RNAP6ALH (‘non-swiveled’, blue). (B) Analysis of β′SI3 dynamics by multibody refinement in the RNAPFree (left) and RNAPTTC (right). The β′SI3 domain shows a limited movement in the RNAPFree, whereas it displays a large dynamic in the RNAPTTC. In the RNAPFree, the β′SI3 domain has a limited mobility, with a rotation amplitude of up to ~7°. In the RNAPTTC, the β′SI3 domain displays a large flexibility with up to ~45° of inward rotation, showing alternate between the ‘in’ (orange) and ‘out’ (green) conformations. (C) Eigen-values distribution along the first two principal components of the Multibody analysis for RNAPFree and RNAPTTC. [file NIHMS1881320-supplement-7.tif]

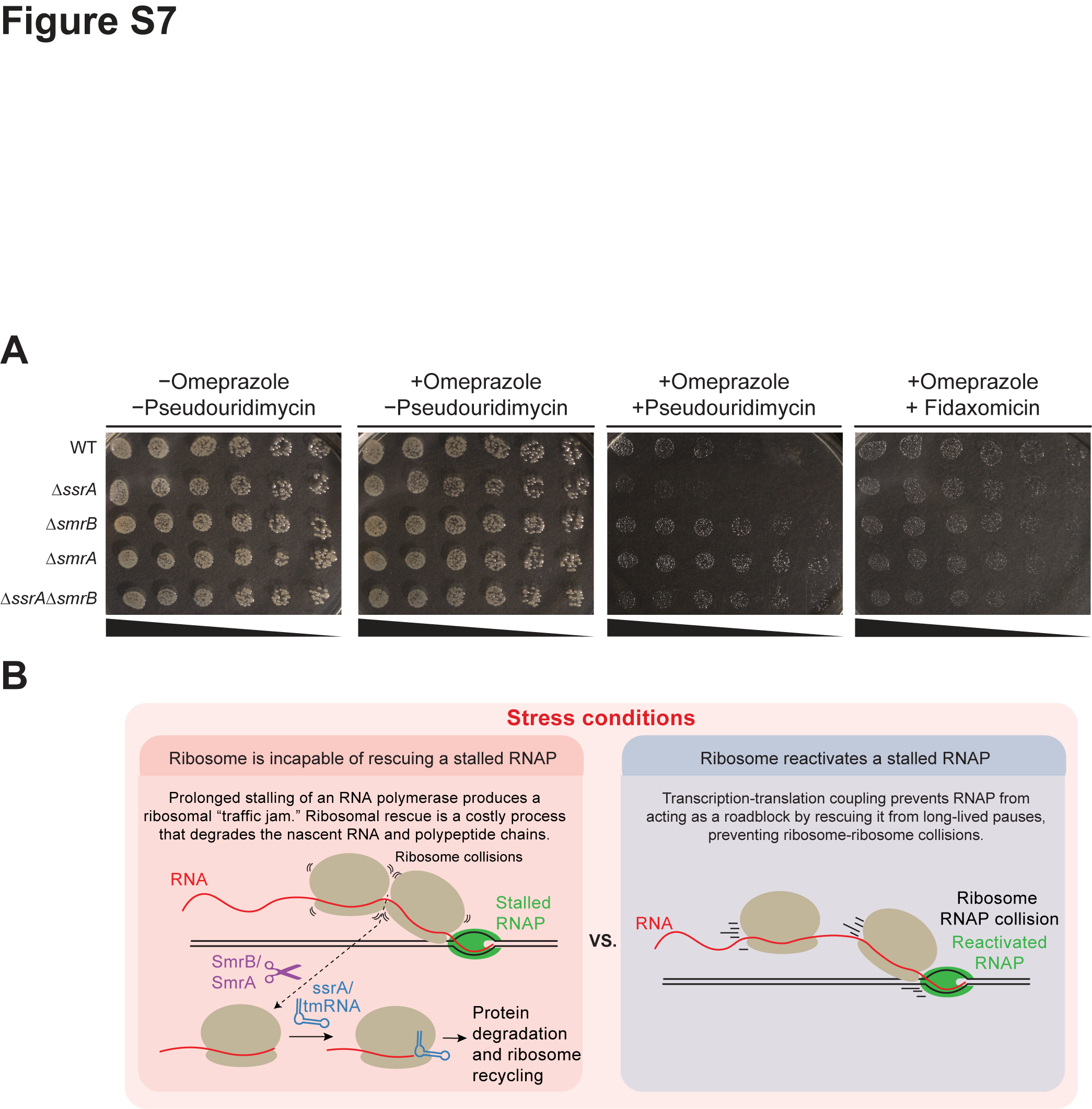

Supplement: 8 — Figure S7. RNAP stalling causes ribosome-ribosome collisions, related to STAR Methods (A) Sections of agar plates showing growth of two-fold serial dilutions of E.coli strains (left label), with or without transcription elongation inhibitor pseudouridimycin (250 μg/ml) or transcription initiation inhibitor fidaxomicin (75 μg/ml). To improve the efficiency of antibiotic incorporation, Mueller Hinton agar was used and the cells were also treated with omeprazole (250 μg/ml). (B) Model for RNAP stalling causing ribosome-ribosome collisions and their prevention by a coupled ribosome. [file NIHMS1881320-supplement-8.tif]
